# Genes to specialized metabolites: accumulation of scopoletin, umbelliferone and their glycosides in natural populations of *Arabidopsis thaliana*

**DOI:** 10.1186/s12870-024-05491-w

**Published:** 2024-08-27

**Authors:** Anna Ihnatowicz, Joanna Siwinska, Izabela Perkowska, Jeremy Grosjean, Alain Hehn, Frederic Bourgaud, Ewa Lojkowska, Alexandre Olry

**Affiliations:** 1 Laboratory of Plant Protection and Biotechnology, Intercollegiate Faculty of Biotechnology of University of Gdansk and Medical University of Gdansk, University of Gdansk, Abrahama 58, Gdansk, 80-307 Poland; 2 Université de Lorraine-INRAE, LAE, Nancy, F-54000 France

**Keywords:** Accessions, Coumarins, Genetic variation, Gene expression, Metabolic profiling, In vitro liquid culture, Scopolin, Skimmin, Soil environment

## Abstract

**Background:**

Scopoletin and umbelliferone belong to coumarins, which are plant specialized metabolites with potent and wide biological activities, the accumulation of which is induced by various environmental stresses. Coumarins have been detected in various plant species, including medicinal plants and the model organism *Arabidopsis thaliana*. In recent years, key role of coumarins in maintaining iron (Fe) homeostasis in plants has been demonstrated, as well as their significant impact on the rhizosphere microbiome through exudates secreted into the soil environment. Several mechanisms underlying these processes require clarification. Previously, we demonstrated that Arabidopsis is an excellent model for studying genetic variation and molecular basis of coumarin accumulation in plants.

**Results:**

Here, through targeted metabolic profiling and gene expression analysis, the gene-metabolite network of scopoletin and umbelliferone accumulation was examined in more detail in selected Arabidopsis accessions (Col-0, Est-1, Tsu-1) undergoing different culture conditions and characterized by variation in coumarin content. The highest accumulation of coumarins was detected in roots grown in vitro liquid culture. The expression of 10 phenylpropanoid genes (*4CL1*,* 4CL2*,* 4CL3*,* CCoAOMT1*,* C3’H*,* HCT*,* F6’H1*,* F6’H2*,*CCR1* and *CCR2*) was assessed by qPCR in three genetic backgrounds, cultured in vitro and in soil, and in two types of tissues (leaves and roots). We not only detected the expected variability in gene expression and coumarin accumulation among Arabidopsis accessions, but also found interesting polymorphisms in the coding sequences of the selected genes through in silico analysis and resequencing.

**Conclusions:**

To the best of our knowledge, this is the first study comparing accumulation of simple coumarins and expression of phenylpropanoid-related genes in Arabidopsis accessions grown in soil and in liquid cultures. The large variations we detected in the content of coumarins and gene expression are genetically determined, but also tissue and culture dependent. It is particularly important considering that growing plants in liquid media is a widely used technology that provides a large amount of root tissue suitable for metabolomics. Research on differential accumulation of coumarins and related gene expression will be useful in future studies aimed at better understanding the physiological role of coumarins in roots and the surrounding environments.

**Supplementary Information:**

The online version contains supplementary material available at 10.1186/s12870-024-05491-w.

## Background

Plants produce a wide range of specialized metabolites, among which phenylpropanoids constitute a large class. Their biosynthesis is a very complex and branched pathway, and their functions are, so far, not fully understood. The most studied phenylpropanoids are lignins, flavonoids, anthocyanins, chalcones and coumarins. Coumarins are widely produced by plants and were described as phytoalexins [1]. Scopoletin (7-hydroxy-6-methoxycoumarin), also reported as hydroxycoumarin is synthesized, among others, by diverse medicinal plants [2], cassava [3], sweet potato [4], sunflower [5], cotton [6], and the model plants tobacco (*Nicotiana tabacum*) and *Arabidopsis thaliana* [7]. In Arabidopsis, scopoletin and its glycosylated form scopolin (β-D-glucoside scopoletin) were firstly reported by Rohde et al. [8] and Bednarek et al. [9]. One year later, Kai et al. [10] showed additionally the presence of trace amounts of skimmin (glycosylated form of 7-hydroxycoumarin also known as umbelliferone) in the roots, as well as esculetin (6,7-dihydroxycoumarin) both in roots and shoots. Scopoletin and its glycoside, scopolin, are the major coumarins accumulating in Arabidopsis roots [10–16]. Our research team has demonstrated as the first one that Arabidopsis is an excellent model for studying the genetic basis of natural variation in coumarin biosynthesis by conducting a quantitative trait locus (QTL) mapping followed by identification of new potential candidate loci [13]. We then extended this work and showed that scopoletin was the most abundant coumarin compound in the roots of each of the 28 Arabidopsis accessions tested, but interestingly it was also detected in leaf extracts [16]. The latter is supported by the results of Robe et al. [17], which confirmed that scopoletin is synthesized in the roots, but can move throughout the plant body within the xylem sap and accumulate in the shoots. In the same study [16], we demonstrated for the first time in Arabidopsis the presence of small amounts of umbelliferone in hydrolyzed extracts prepared from the roots of all 28 tested accessions. Umbelliferone is an important intermediate for the biosynthesis of more complex coumarins – furanocoumarins and pyranocoumarins [18], which are of great importance in the pharmaceutical industry. Even if Arabidopsis does not produce furanocoumarins, our discovery of umbelliferone accumulation in Arabidopsis is a significant step in the study of coumarin biosynthesis using this model plant. However, we still do not know how umbelliferone is synthesized in Arabidopsis and it cannot be excluded to be only an intermediate in the synthesis of skimmin.

Recently, our research team and several other groups, demonstrated that coumarins, namely fraxetin, sideretin and scopoletin, play a crucial role in Fe chelation in Arabidopsis and secretion of coumarins by Arabidopsis roots was shown to be induced under Fe-deficiency [12, 14, 19–22]. Accumulation and secretion of coumarinolignans and other coumarins was also shown to be induced in Arabidopsis roots in response to Fe-deficiency at high pH [23]. Moreover, the excretion of an Fe-mobilizing scopoletin, which is regulated by the root-specific transcription factor MYB72, was revealed to have selective antimicrobial activity that shapes the root-associated microbial community [24]. Various studies have documented the antibacterial and antifungal effects of scopoletin and its derivatives. Scopoletin was described to be involved in the plant immune response in defense reactions to pathogens [25, 26] such as *Fusarium oxysporum*, *Fusarium solani*, *Rhizopus stolonifer*, and *Lasiodiplodia theobromae* [27], tobacco mosaic virus [28]. Scopoletin displays a higher growth inhibition effect on *F. oxysporum* than its β-D- glucoside scopolin [27] suggesting that the aglycon might be responsible for the defense reactions. We also recently investigated the mechanisms underlying the interplay between coumarin accumulation, Fe status, and plant pathogen resistance using the Arabidopsis/*Dickeya* spp. pathosystem. We observed that the response of different Arabidopsis lines (mutants defective in coumarin biosynthesis and transport) was dependent on the *Dickeya* species used and the genotype of plants grown in a Fe-deficient hydroponic culture [29].

Although coumarins are well known for their potent antibacterial and antifungal properties, they have recently received much attention as important factors influencing a number of processes that determine the interaction of plants with the soil environment, both biotic and abiotic factors [14, 15, 24, 28, 30–32]. Scopoletin was discovered as a new signal in the pre-penetration dialogue in plant-mycorrhizal associations that possibly have implications for chemical communication [33]. As shown by Cosme et al. [33], the coumarin scopoletin particularly stimulates pre-penetration development and metabolism in mycorrhizal fungi. The production of both scopoletin and fraxetin [described by 14, 21, 22] impact the root microbiota as shown by Harbort et al. [31]. Their biosynthesis and secretion through PDR9 (plasma membrane-bound transporter described by [34] are determining root microbiota composition in a naturally Fe-limiting calcareous soil [31]. The important role of coumarins in communication on the microbiome-root-shoot axis, alongside strigolactones and flavonoids [32], is currently vigorously discussed.

**Fig. 1:**
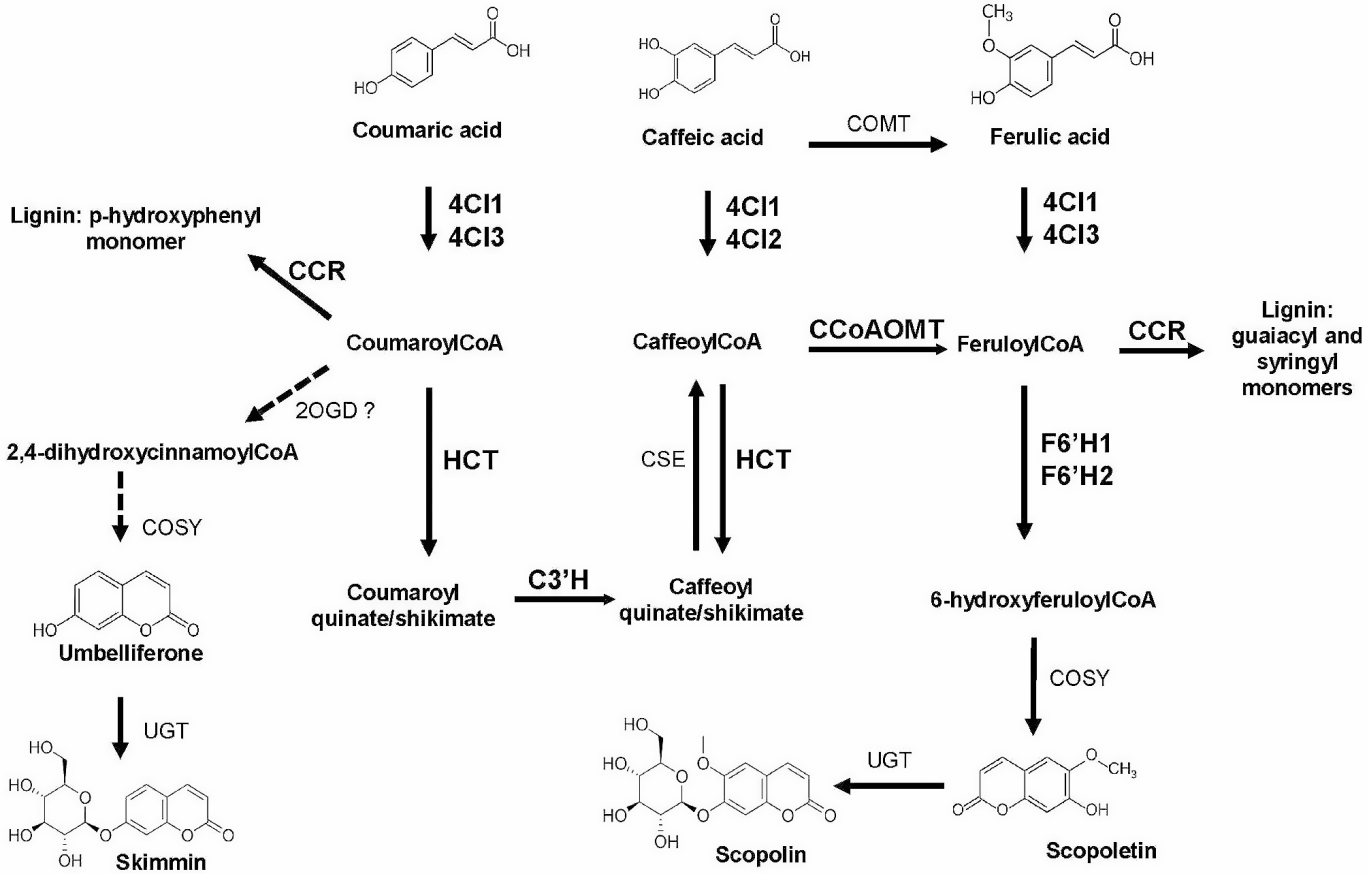
Simplified schematic representation of the biosynthetic pathway of scopoletin, umbelliferone and their corresponding glycosides, scopolin and skimmin respectively, along with monolignols in *Arabidopsis thaliana*. The step of umbelliferone synthesis was hypothesized to occur via the same pathway as scopoletin synthesis. Genes whose expression was studied are highlighted: **HCT**, Hydroxycinnamoyl CoenzymeA shikimate: quinate hydroxycinnamoyltransferase; **C3’H**, Coumarate p-coumaroyl shikimate 3’-hydroxylase; **F6’H1**,** F6’H2**, Feruloyl-CoA 6’-Hydroxylase1 and 2; **4CL1**,** 4CL2**,** 4CL3**, 4-Coumarate CoenzymeA ligase 1–3; **CCoAOMT**, Caffeoyl CoenzymeA 3-o-methyltransferase; **CCR1**,** CCR2**, Cinnamoyl CoA reductase 1 and 2. Other enzymes shown in the diagram are: COSY, coumarin synthesis; putative UGT, UDP-glucosyltransferase. Adapted from [36] and [37]

Here we focused on selected simple coumarins: (1) scopoletin together with its glycoside scopolin, which are the main coumarins of Arabidopsis, (2) and umbelliferone that was recently detected for the first time by our group in this model plant [16] with its glycoside, skimmin. It should be remembered that other coumarin compounds like fraxetin, sideretin [21, 35] and esculetin are accumulated in Arabidopsis, whose biosynthesis and functions in plants also require further elucidation. The biosynthetic pathway leading to esculetin, which is postulated to be a strong Fe chelator due to the catechol functional group [20], is largely unclear in plants and remains a mystery in Arabidopsis. In this work, in addition to targeted metabolic profiling, we focused on the analysis of the expression of genes directly involved in scopoletin biosynthesis, but also of a number of genes encoding enzymes of the phenylpropanoid pathway located upstream and downstream to the biosynthesis of coumarins (Fig. 1). We detected significant variations in the content of coumarins and gene expression levels that were not only genetically determined but also tissue and culture dependent. The latter is particularly important considering that these are two different growing conditions, widely used by other authors and to the best of our knowledge, with no comparison between them in the context of coumarin accumulation until this article. Thus, by analyzing the differential expression patterns of selected genes in leaves and roots of three Arabidopsis genetic backgrounds and two contrasting environments, we can better understand the correlation between genetic variants (coding sequences) and phenotypic variation (coumarin content), especially in the context of the unknown biosynthesis of umbelliferone (Fig. 1).

## Methods

### Growth conditions in vitro culture

Arabidopsis seeds were surface sterilized and sown on 0.5x Murashige and Skoog (MS) medium as described by Siwinska et al. [13]. After stratification at 4 °C for 72 h in the dark, the plates were placed in a phytotron for another 10 days (light intensity 35 µmol m^− 2^ s^− 1^, 20 °C day/18 °C night, photoperiod 16/8). Then, 10-day-old seedlings were transferred and grown in glass culture vessels with liquid medium according to Siwinska et al. [13]. On day 28 of cultivation (and after 17 days of growing Arabidopsis in in vitro liquid cultures on rotary platform shakers), the plants were harvested, leaves and roots weighed separately, frozen in liquid nitrogen and stored at -80 °C (in 2 ml microtubes). For all Arabidopsis accessions, three biological replicates were grown (in three independent glass culture vessels, at least three seedlings in each vessel).

### Growth conditions in soil

Arabidopsis seeds were first stratified (at 4 °C for 4 days on water-soaked Whatman paper) and then sown into a soil mixture (commercially available Compo Sana soil with vermiculite in a 3:1 ratio). The plants were watered as it was required and once a week with the soil fertilizer (Substral). After 3 weeks, plants were collected (leaves and roots separately), weighed and frozen in 2 ml microtubes in liquid nitrogen and stored at -80 °C until further analysis. All Arabidopsis accessions were grown in three biological replicates (in independent pots). HOBO U12 data logger (Onset Computer Corporation, Bourne, MA) was used for monitoring plant growth conditions.

### Preparation of root methanol extracts

The previously harvested plant material was divided into four sets according to the coumarins extraction method. The tissues in the first set (M-H-) were homogenized using steel beads and sonication, after that the homogenate was soaked with 80% methanol supplemented with 0.5 µM 4-methylumbelliferone and kept at 4 °C for 24 h. The methanol extracts were then centrifuged for 20 min at 13,000 x g. The second set (M + H+) was additionally incubated for 5 min in the microwave oven set at 700 W before the homogenization. After centrifugation, extracts were enzymatically hydrolyzed according to modified protocol of Nguyen et al. [38] as described in Siwinska et al. [13]. The third set of extracts was incubated only in a microwave oven (M + H-), while the fourth was only subjected to enzymatic hydrolysis (M-H+).

### Chemicals

Coumarin standards umbelliferone (purity ≥ 99%), esculin (glycosylated esculetin, > 98% purity) were purchased from Sigma-Aldrich (St. Louis, USA), scopoletin (> 95% purity) and esculetin (> 98% purity) from Extrasynthese (Genay, France), skimmin (98% purity, glycosylated umbelliferone) from Biopurify Phytochemicals (Chengdu, China), scopolin (> 98% purity, glycosylated scopoletin) from Aktin Chemicals Inc. (Chengdu, China). Stock solutions of each standard (at 10 mmol/L concentrations) were made by diluting the powder in dimethyl sulfoxide (Fisher scientific, Illkirch, France), which were subsequently kept at -18 °C. The methanol (HPLC-grade) was purchased from CarloErba Reagents (Val de Reuil, France), formic acid was purchased from Fisher Scientific (Illkirch, France). PURELAB Ultra system (Veolia Water S.T.I., Antony, France) was used for water purification.

### Quantification of coumarins by UHPLC-MS targeted metabolite profiling

Targeted metabolite profiling of Arabidopsis methanol extracts prepared from roots and leaves was performed, namely quantification of selected coumarins using ultra-high-performance liquid chromatography combined with mass spectrometry analysis (UHPLC-MS), as described in the work of Perkowska et al. [16].

### Quantitative real-time PCR analysis

Total RNA was isolated from Col-0, Est-1 and Tsu-1 Arabidopsis accessions grown in vitro in liquid culture and in soil according to Ihnatowicz et al. [39]. We conducted the quantitative real-time PCR analysis by using LightCycler^®^ 480 Real-Time PCR System (Roche) with the Luminaris**™** HiGreen qPCR Master Mix (Thermo Scientific) and primer sequences for genes summarized in Supplementary Table S1 (gene-specific) and for *ACTIN2* in [40]. Confirmation of primer specificity and normalization of relative transcript levels of the studied genes were performed as described in Ihnatowicz et al. [39]; the efficiencies of the PCR product amplification by the qPCR primers are provided in Supplementary Table S2.

### Sequencing

PCR reactions were carried out in a 10 µl reaction mixture, which contained cDNA synthetized based on root RNA, 0.5 U of TaKaRa LA Taq^®^ DNA polymerase, 200 µM dNTP, 1 µM primers, and 1 × LA PCR Buffer ll (Mg2 + plus). After denaturation at 94 °C for 1 min, the reaction mixture was used in PCR amplification using 34 cycles of 98 °C for 10 s, 60 °C for 20 s, and 68 °C for 60 s in the Thermal Cycler C1000 Touch (Bio-Rad). Gene-specific primers used for gene amplification of CDS are summarized in Supplementary Table S3. PCR products were cloned into pGEM^®^-T vector. The *Escherichia coli* strain Gene Hogs (F- mcrA Δ(mrr-hsdRMS-mcrBC) φ80lacZΔM15 ΔlacX74 recA1 araD139 Δ(ara-leu)7697 galU galK rpsL (StrR) endA1 nupG fhuA::IS2) was used for plasmid amplification and maintenance. The vector specific primers M13pUCf and M13pUCr and BigDye^®^ Terminator v3.1 (Life Technologies) were used for the sequencing of positive clones. The reaction products were sequenced by 3730xl DNA Analyzer, while sequence alignments were performed using CLUSTALW [41]. Multiple sequence alignment (MSA) indicating similar/different nucleotides/amino acids across the aligned sequences were visualized using BioEdit v5.0.9 software.

### Statistical analysis

We processed data and conducted statistics (pairwise comparisons with Welch’s t-test: two-sample assuming unequal variances) using R programming (https://app.displayr.com) and Microsoft Excel. Three biological replicates were included in all treatments. In the figures, means and error bars for absolute deviations are shown. The data points with significantly different mean values are indicated with asterisk(s), with the significance level of *p* < 0.001 (***), *p* < 0.01 (**) and *p* < 0.05 (*).

## Results

### Methodology for the analysis of coumarin content in Arabidopsis extracts

The vast majority of coumarins present in plant cells are bound to sugars [42]. To obtain a global overview of the concentration of coumarins, we performed methanol/water (80:20) extraction of various tissues cultured in vitro and in soil under selected conditions. Half of the crude extracts were enzymatically hydrolyzed using β-glucosidase to analyze the total free scopoletin and umbelliferone. The quantification of their respective glycosylated counterparts, scopolin and skimmin, was performed on extracts that had not undergone enzymatic treatment. In parallel, as a control mode, all simple coumarins were quantified using the UHPLC method in the methanol extracts, both those subjected to enzymatic hydrolysis and those that were not hydrolyzed. Various data can be found in the literature regarding the effect of microwave oven on the activity of β-glucosidases naturally occurring in plant tissues and its use as a useful tool in the analysis of plant extracts [43, 44]. We decided to test it by using a microwave oven treatment in two sets of our plant extracts, both those subjected to enzymatic hydrolysis and those without it. Based on the results obtained, it cannot be concluded whether the microwave oven inhibited the β-glucosidase activity. However, it turned out that microwave treatment led to a reduction in the levels of all tested coumarins when compared to the untreated plant extracts, either through the direct energy distribution of the coumarin compounds or through the emitted heat (Figure S1, Figure S2). Additionally, the mean deviations were much higher in some microwave-treated samples for umbelliferone quantification (Figure S2). Therefore, we further quantified coumarins only in extracts that were not subjected to microwave oven treatment.

### Quantification of free coumarins by UHPLC

**Fig. 2:**
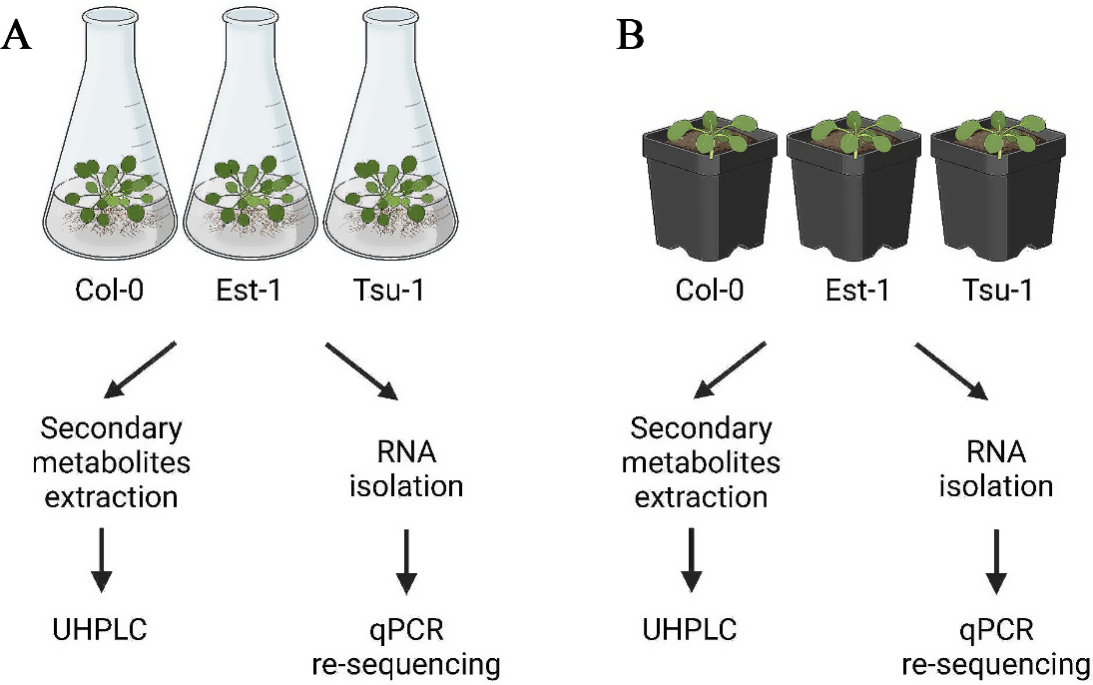
Experimental scheme showing two types of culture used (**A**) in vitro liquid culture and (**B**) soil condition. Plants were grown in soil (optimal soil mix supplemented with fertilizer once per week) and in in vitro liquid cultures (light intensity 35 µmol m^− 2^ s^− 1^, 20 °C day/18 °C night, photoperiod 16/8). Leaves and roots were harvested separately. All samples were divided for secondary metabolites extraction and RNA isolation. Plant extracts were divided in half and subsequent UHPLC analysis were performed in methanol extracts with and without enzymatic treatment. cDNA was synthetized for qPCR analysis. Created with BioRender.com

To investigate the natural variability of scopoletin and umbelliferone, we focused on both roots and leaves of 3 Arabidopsis accessions (Col-0, Est-1, and Tsu-1) grown in different culture types (Fig. 2). We grew plants in vitro in liquid cultures - conditions that induce the growth of roots, which are the main tissue accumulating coumarins. Additionally, in this type of cultures, access to the roots is simple, which makes it easier to collect them for further analysis. This method of growing plants also mimics stressful conditions that might induce coumarin accumulation. In vitro liquid cultures were performed in light, which was reported to specifically increase phenylpropanoid production in roots [45]. In parallel, we cultivated plants in more physiological conditions, in soil enriched with fertilizer once a week, in accordance with the optimal growth conditions for Arabidopsis described in the literature [39].

**Fig. 3:**
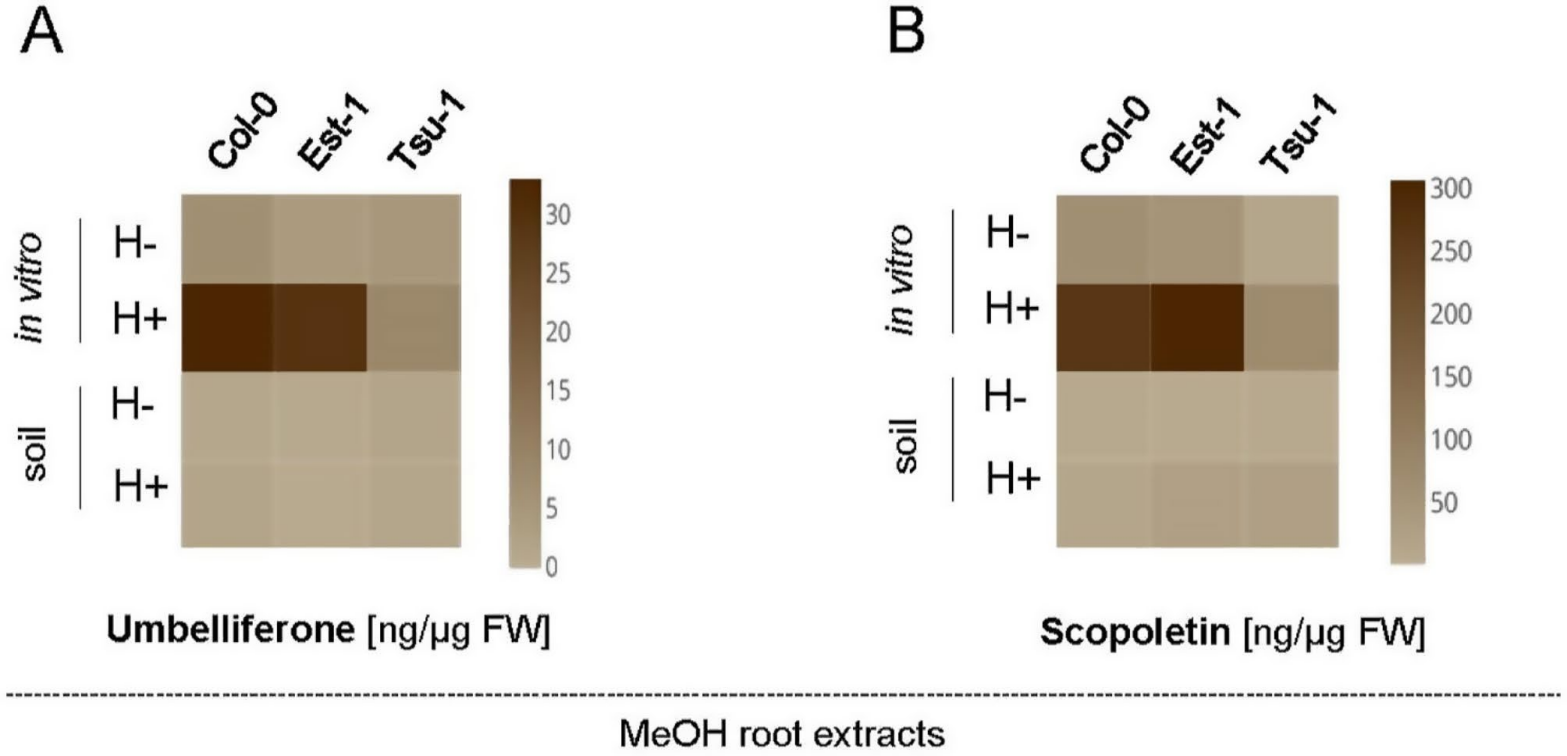
Heat maps showing the quantification of umbelliferone (**A**) and scopoletin (**B**) in the Arabidopsis root extracts before (H-) and after (H+) enzymatic hydrolysis. Methanol extracts were prepared from Col-0, Est-1 and Tsu-1 Arabidopsis accessions grown in in vitro liquid culture and in soil, quantified by UHPLC. The color scale represents the compound concentration given in ng/µg FW (fresh weight); dark brown indicates high concentration and light brown denotes low concentration

Our results clearly showed that coumarin production was significantly higher in roots of Arabidopsis plants grown under stress conditions in in vitro liquid cultures when compared to soil-grown plants (Fig. 3). As expected, free coumarins, namely umbelliferone and scopoletin were mostly accumulated in MeOH roots extracts subjected to enzymatic hydrolysis (panels H + in Fig. 3A and B). These methanol extracts from Col-0 and Est-1 roots grown in vitro contain similar concentrations of scopoletin and umbelliferone, whereas Tsu-1 accumulates visibly lower levels of both molecules (Fig. 3AB). In the roots of all accessions grown in soil we detected significantly lower concentrations of the tested coumarins compared to the plants grown in vitro. The observed differences in coumarin content among plant genotypes grown in soil were minimal (Fig. 3AB).

**Fig. 4:**
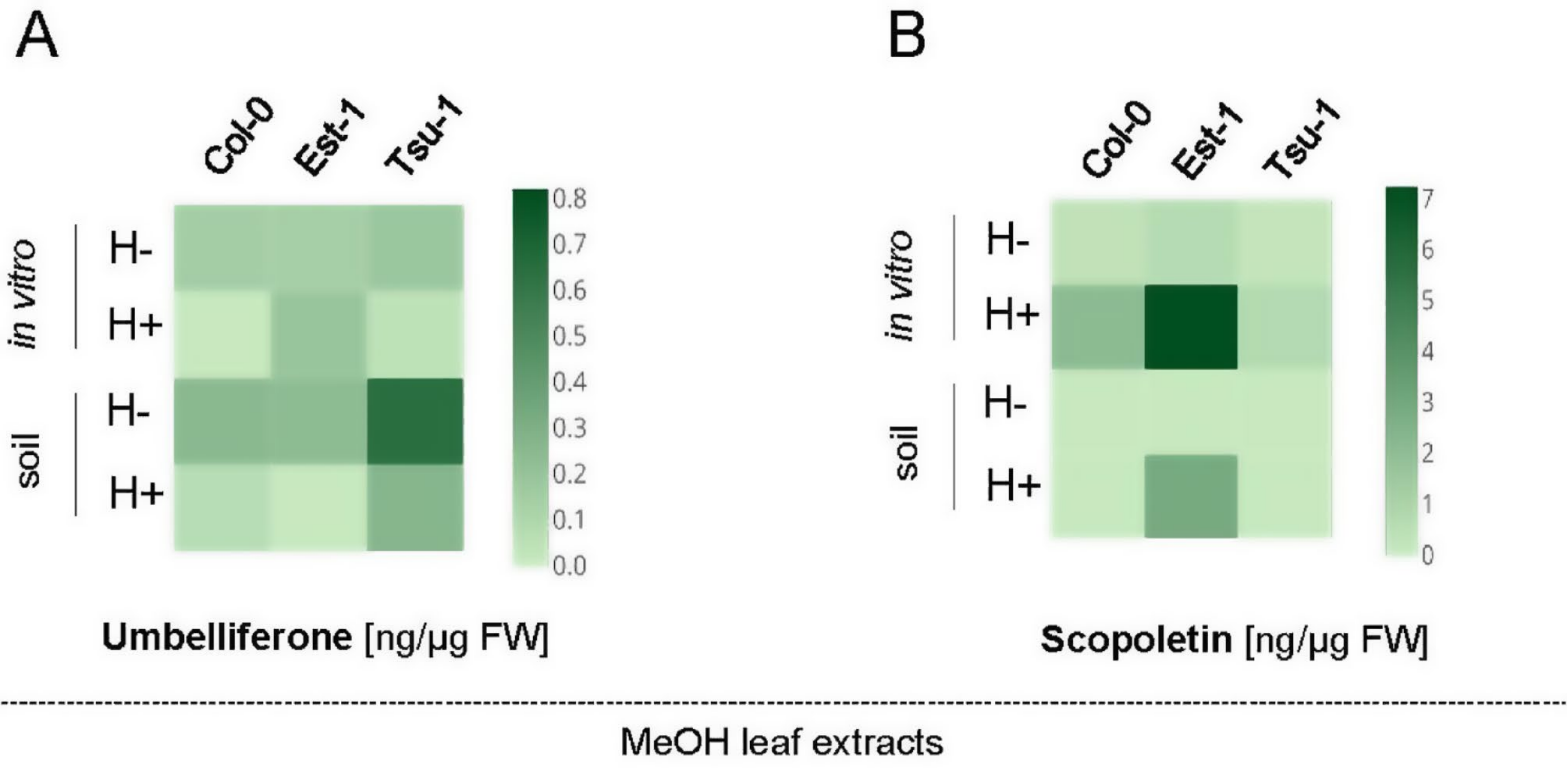
Heat maps showing the quantification of umbelliferone (**A**) and scopoletin (**B**) in the Arabidopsis leaf extracts before (H-) and after (H+) enzymatic hydrolysis. Methanol extracts were prepared from Col-0, Est-1 and Tsu-1 Arabidopsis accessions grown in in vitro liquid culture and in soil, quantified by UHPLC. The color scale represents the compound concentration given in ng/µg FW (fresh weight); dark brown indicates high concentration and light brown denotes low concentration

Quantitative determination of coumarins in MeOH leaf extracts detected relatively high concentration of umbelliferon in the leaves of Tsu-1 genotype (compared to the reference Col-0 line) grown in soil (Fig. 4A). Unexpectedly, umbelliferone was mainly present in the MeOH leaf extracts without enzymatic hydrolysis (panel H- in Fig. 4A). In parallel, we detected relatively high levels of the major free Arabidopsis coumarin, scopoletin, in the MeOH leaf extracts prepared from the tissues of in vitro grown plants after enzymatic hydrolysis, but also in the soil-grown roots (panel H + in Fig. 4B). However, overall, higher levels of scopoletin were accumulated in leaves of plants grown in vitro compared to plants grown on soil (Fig. 4B).

In summary, under all tested conditions, both coumarin compounds were produced in small amounts in plant leaves (Fig. 4) and in larger quantities in roots (Fig. 3), which is consistent with the results of previous studies indicating that coumarins accumulate mainly in underground tissues [9, 16].

### Quantification of glycosylated coumarins by UHPLC

**Fig. 5:**
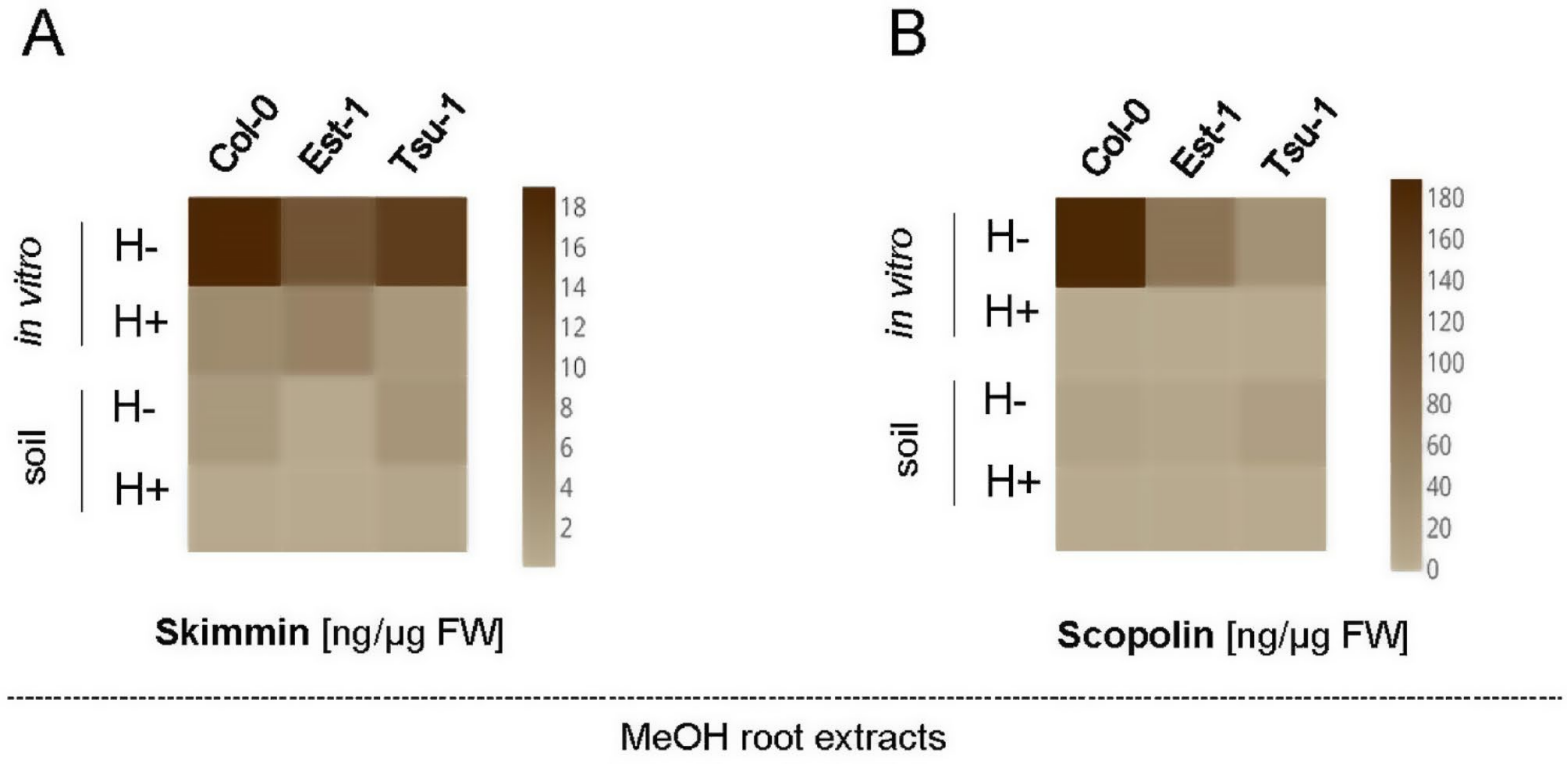
Heat maps showing the quantification of skimmin (**A**) and scopolin (**B**) in the Arabidopsis root extracts before (H-) and after (H+) enzymatic hydrolysis. Methanol extracts were prepared from Col-0, Est-1 and Tsu-1 Arabidopsis accessions grown in in vitro liquid culture and in soil, quantified by UHPLC. The color scale represents the compound concentration given in ng/µg FW (fresh weight); dark brown indicates high concentration and light brown denotes low concentration

As determined in our preliminary experiments, we performed the quantification of the glycosylated compounds in plant extracts without microwave treatment. The majority of glycosylated coumarins were expected to be detected in samples not subjected to enzymatic hydrolysis (H-), but as a control we quantified their levels also in samples treated with β-glucosidase (H+). Scopolin and skimmin were detected mostly in untreated root extracts (H-) in both in vitro and soil-grown plants (in Fig. 5AB), whereas some skimmin quantities were also identified in root samples subjected to enzymatic hydrolysis (H+) (Fig. 5A). Taking into account the scale presented in Fig. 5AB, the concentration of skimmin in the roots was about ten times lower than concentration of scopolin. From a general point of view, it is worth noting that glycosylated coumarins in the roots of plants grown in soil were synthesized in much smaller amounts compared to plants grown in vitro. Regarding the genetic background specificity, scopolin was mostly detected in roots of Col-0 grown in vitro, while under this condition the lowest concentration was detected in Tsu-1 (Fig. 5B).

**Fig. 6:**
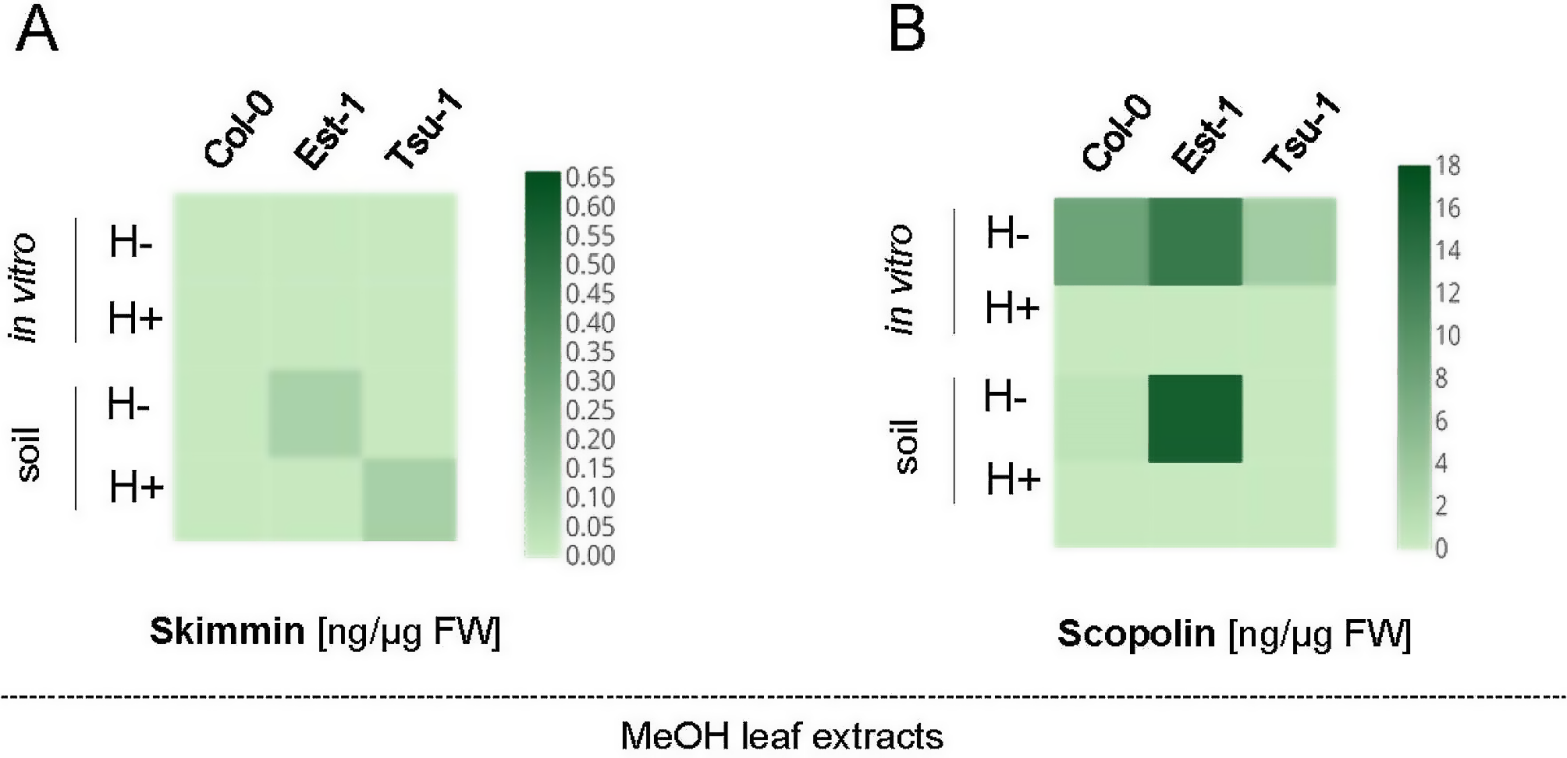
Heat maps showing the quantification of skimmin (**A**) and scopolin (**B**) in the Arabidopsis leaf extracts before (H-) and after (H+) enzymatic hydrolysis. Methanol extracts were prepared from Col-0, Est-1 and Tsu-1 Arabidopsis accessions grown in in vitro liquid culture and in soil, quantified by UHPLC. The color scale represents the compound concentration given in ng/µg FW (fresh weight); dark brown indicates high concentration and light brown denotes low concentration

Concerning the quantification of coumarins in MeOH leaf extracts, the skimmin concentration was similarly low for all the accessions cultivated in vitro but its content was strongly induced in soil-grown Est-1 and Tsu-1 leaves (both H + and H- in Fig. 6A).However, the main coumarin accumulated in the leaves was invariably scopolin, with the highest concentration detected in Est-1 accession both in vitro and in soil conditions (Fig. 6B).

### Quantification of the expression level of genes involved in the synthesis of coumarins

To gain insight into coumarin compounds production at the molecular levels in different Arabidopsis genetic backgrounds, we assessed the expression levels of a number of genes encoding enzymes of the phenylpropanoid pathway located upstream and downstream of coumarin biosynthesis. We conducted real-time quantitative PCR (qPCR) experiments targeting 10 different genes: *4CL1*, *4CL2*, *4CL3*, *CCoAOMT1*, *C3’H*, *HCT*, *F6’H1*, *F6’H2*, *CCR1* and *CCR2* in three Arabidopsis accessions, grown in two conditions (liquid in vitro cultures and soil), and on both kind of tissues (leaves and roots). To be able to make robust comparisons between all samples, the expression was normalized based on the expression of *ACTIN2 (ACT2)* considered as a housekeeping gene [46].

**Fig. 7:**
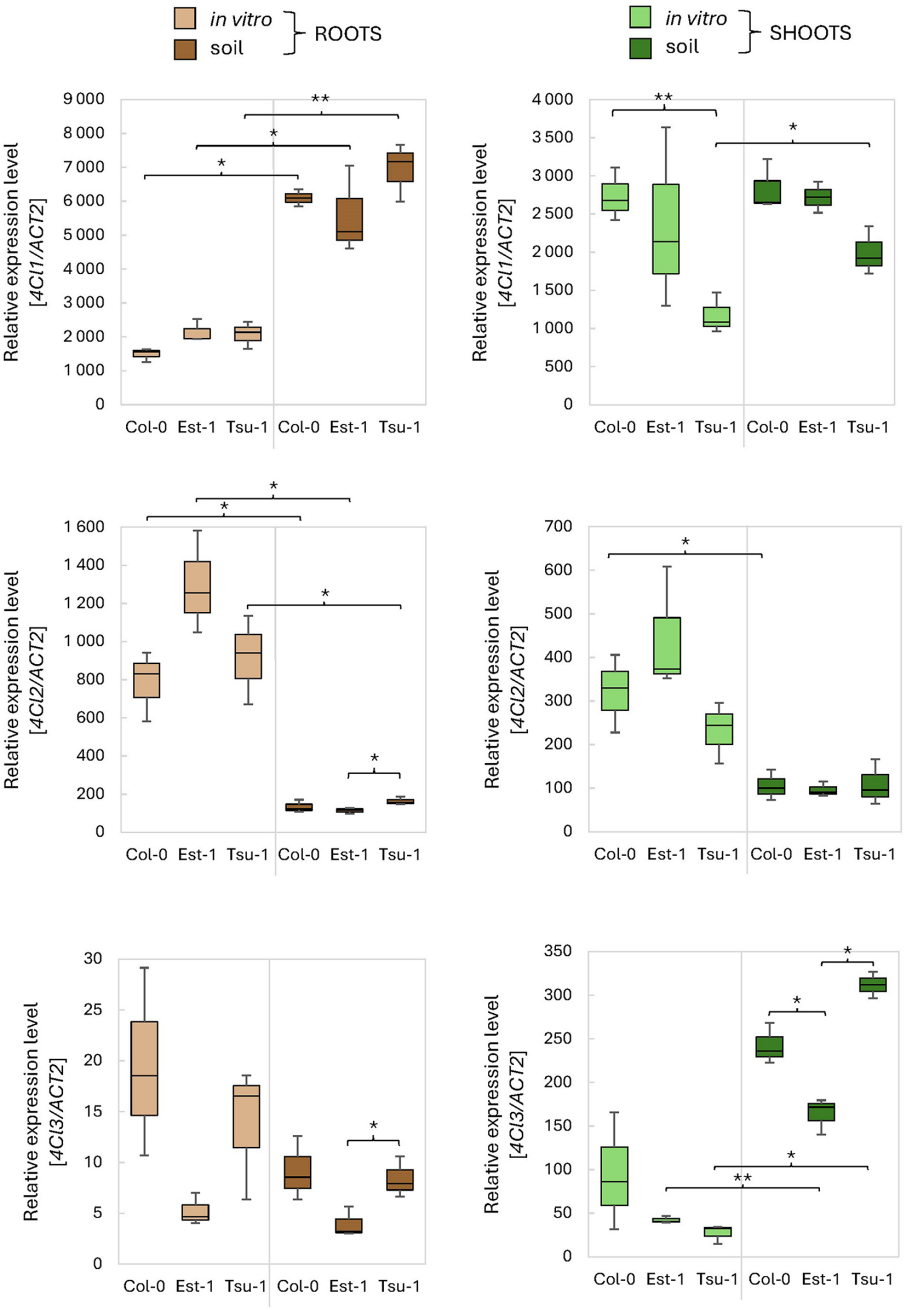
Relative expression levels of the *4CL1-3* genes measured by qPCR. As a reference, the *ACT2* (At3g18780) gene was used. The expression levels were quantified in three Arabidopsis genetic backgrounds (Col-0, Est-1, Tsu-1) grown in in vitro liquid culture and in soil, in two types of tissues (leaves and roots). Means and error bars for absolute deviations are shown. Values: *p* < 0.001 (***), *p* < 0.01 (**) and *p* < 0.05 (*)

Coumaroyl-CoA ligases (4CL) were the first enzymes we focused on. These enzymes are directly related to the synthesis of coumarins. Arabidopsis has four homologous genes encoding 4CLs. Here, we focused of three of them, since *4CL4* has a limited expression profile [37]. The transcription patterns of *4CL1* differ significantly between root samples isolated from soil and in vitro- grown plants for all three accessions (Fig. 7A). However, we did not observe significant differences among genotypes under the same growth conditions. The results were different when we investigated the *4CL1* expression profiles in leaf tissues. The transcription level of *4CL1* was much lower in Tsu-1 accession when compared to Col-0 and Est-1 cultured both in vitro and in soil. The opposite could be observed for the expression profile of *4CL2.* This gene is significantly more expressed in plants grown in vitro compared to soil conditions, in both tissue types - roots and leaves (Fig. 7B). The expression of *4CL3* drops down and is generally much lower in comparison to *4CL1* and *4CL2*, but interestingly it is the most variable between growth condition and among genotypes (Fig. 7C). Moreover, opposite trends in the relative induction of *4CL3* expression between tissues in both types of culture are visible. In leaves it is higher in plants grown in soil compared to in vitro, while in the roots it is the other way around - it is higher in vitro than in plants grown in soil (Fig. 7C).

**Fig. 8:**
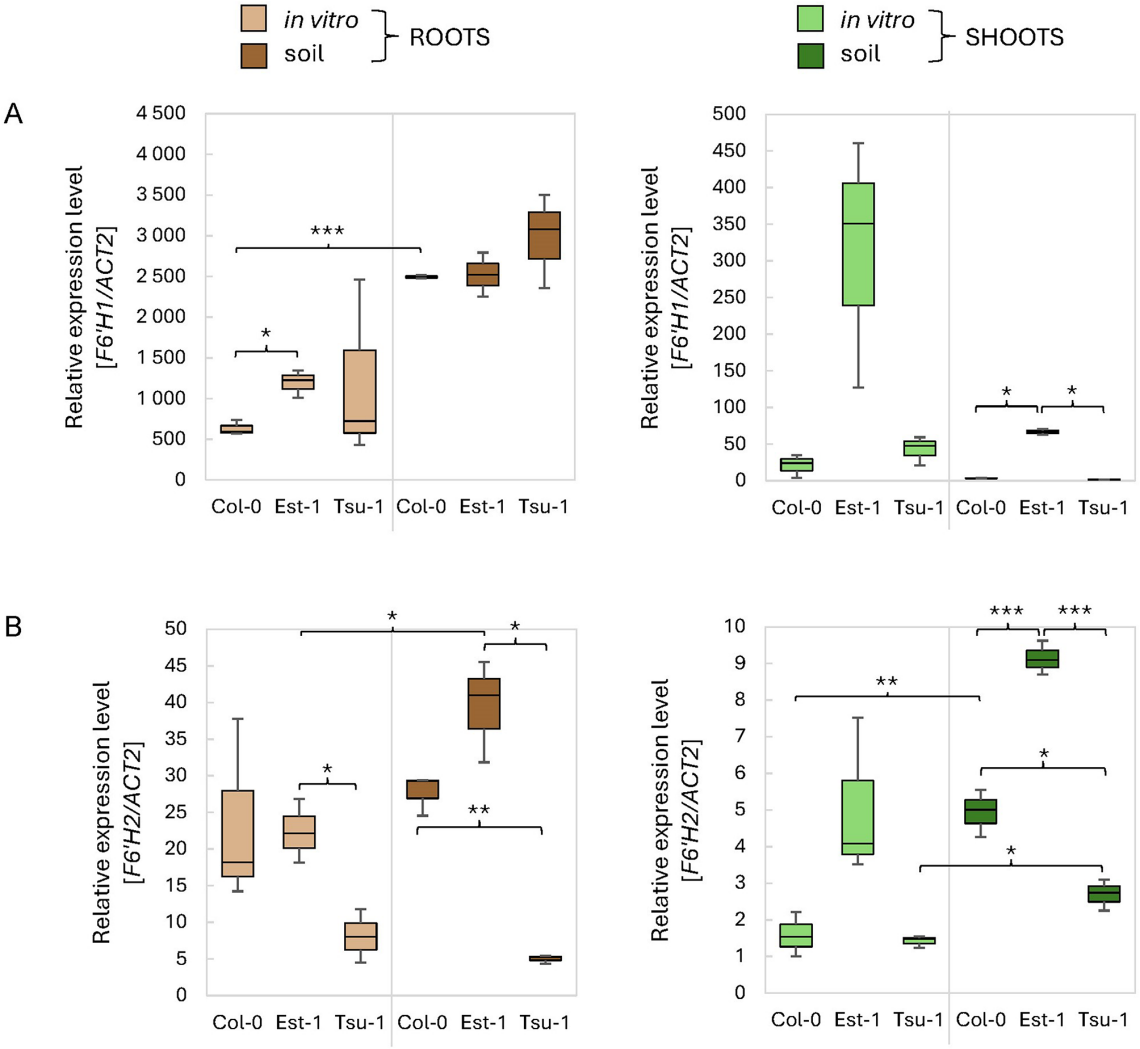
Relative expression levels of (**A**) *F6’H1* and (**B**) *F6’H2* genes involved in the last step of the biosynthesis of the main coumarin (scopoletin) in Arabidopsis measured by qPCR. As a reference, the *ACT2* (At3g18780) gene was used. The expression levels were quantified in three Arabidopsis genetic backgrounds (Col-0, Est-1, Tsu-1) grown in in vitro liquid culture and in soil, in two types of tissues (leaves and roots). Means and error bars for absolute deviations are shown. Values: *p* < 0.001 (***), *p* < 0.01 (**) and *p* < 0.05 (*)

The second gene family we focused on, are the oxoglutarate dependent dioxygenases responsible of the hydroxylation of feruloyl CoA. These enzymes are specific of the biosynthetic pathway of coumarins. As expected, our results confirmed that *F6’H1* and *F6’H2* are particularly more strongly expressed in root tissue, and the *F6’H1* gene is expressed at high level (Fig. 8). When grown in soil, the expression level of *F6’H1* in roots is comparable in all accessions (Fig. 8A), while in leaves it is significantly higher in Est-1 (*p* < 0.01) than in Col-0 or Tsu-1. For plants grown in vitro, the expression of *F6’H1* was more variable. We observed significant differences between different tissues and accessions, but the *F6’H1* transcript was significantly induced in leaves of Est-1 when compared with other genetic backgrounds (Fig. 8A). The expression of *F6’H2* is highly significantly variable among accessions grown in soil. In general, its transcription is more efficient in soil conditions in both leaves and roots. Interestingly, the expression of *F6’H2* is the highest in Est-1 genetic background in all conditions and tissues tested (Fig. 8B). The relatively low expression level of *F6’H2* compared to *F6’H1* indicates its smaller contribution in the biosynthesis of scopoletin.

**Fig. 9:**
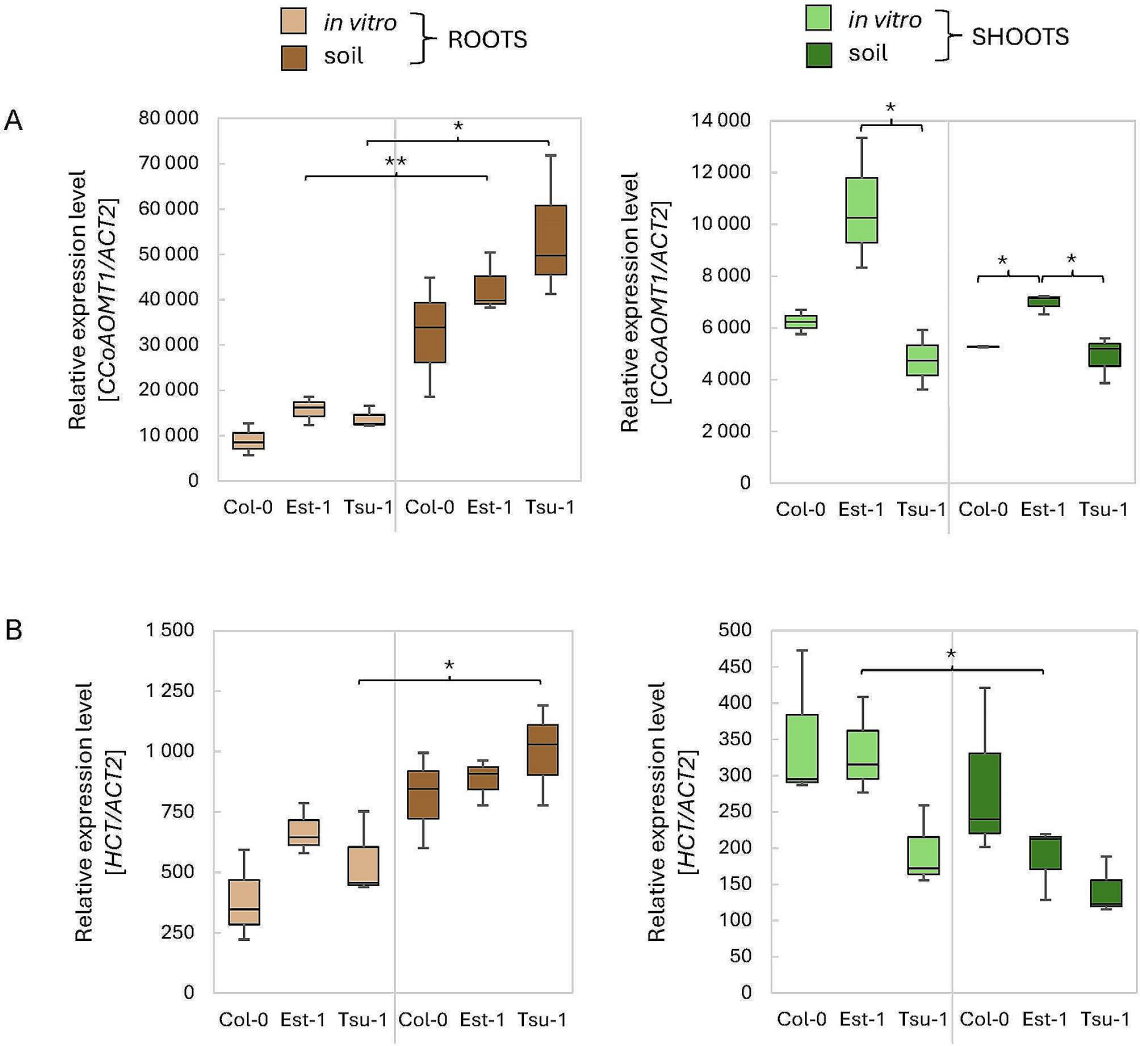
Relative expression levels of the (**A**) *CCoAOMT1* and (**B**) *HCT* genes measured by qPCR. As a reference, the *ACT2* (At3g18780) gene was used. The expression levels were quantified in three Arabidopsis genetic backgrounds (Col-0, Est-1, Tsu-1) grown in in vitro liquid culture and in soil, in two types of tissues (leaves and roots). Means and error bars for absolute deviations are shown. Values: *p* < 0.001 (***), *p* < 0.01 (**) and *p* < 0.05 (*)

Finally, we assessed the expression level of several downstream genes including *CCoAOMT1*,* HCT*,* C3’H*, and *CCRs*. These enzymes are involved in the phenylpropanoid biosynthetic pathway but after the branching point leading the production of coumarins. Our results showed that *CCoAOMT1* and *HCT* have very similar expression patterns in roots, but *CCoAOMT1* is expressed at much higher level (Fig. 9AB). In soil-grown roots, both transcripts have significantly higher values in Tsu-1 compared to other accession (*p* < 0.05).

**Fig. 10:**
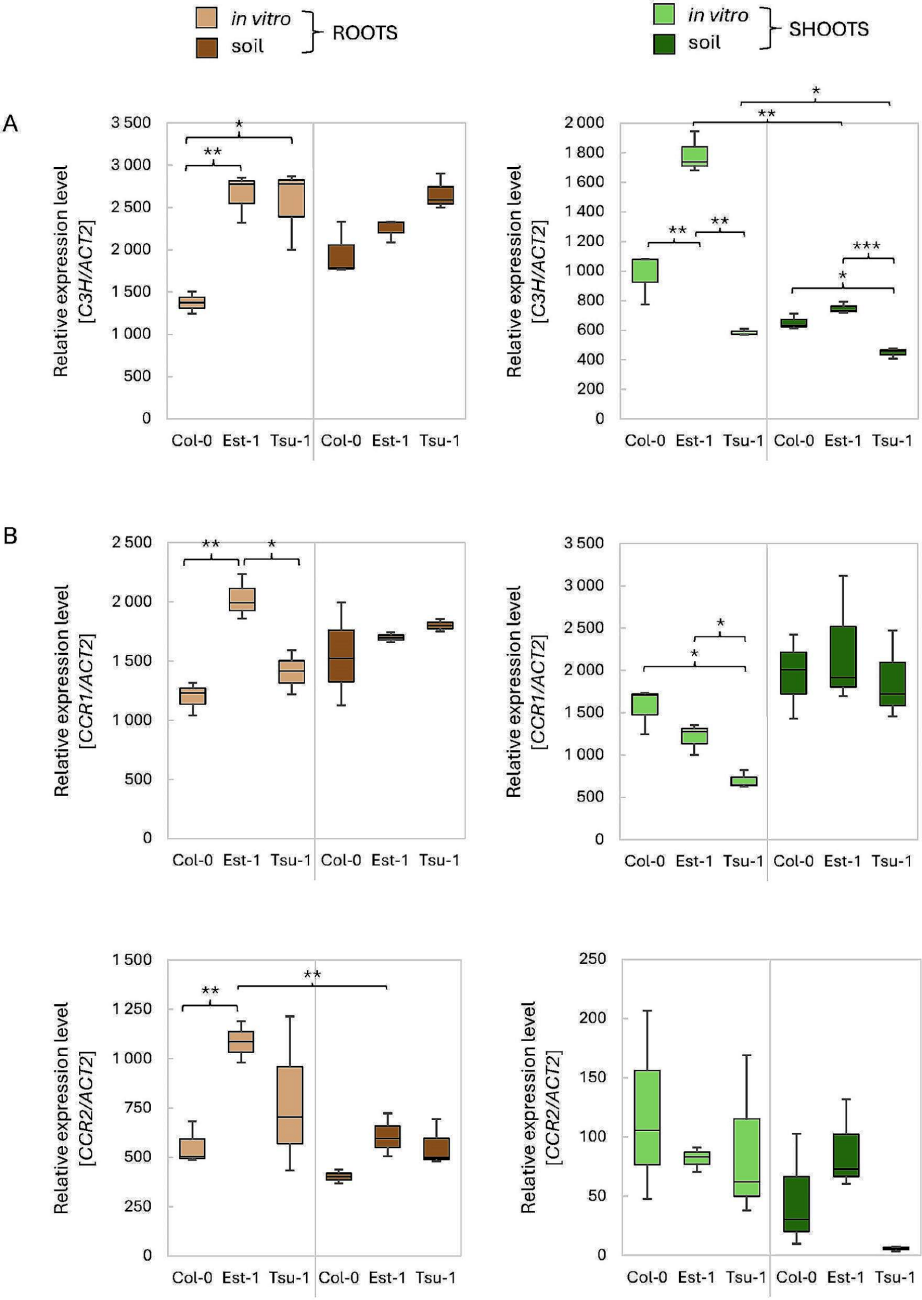
Relative expression levels of the *C3’H* (**A**) and *CCRs* (**B**) genes measured by qPCR. As a reference, the *ACT2* (At3g18780) gene was used. The expression levels were quantified in three Arabidopsis genetic backgrounds (Col-0, Est-1, Tsu-1) grown in in vitro liquid culture and in soil, in two types of tissues (leaves and roots). Means and error bars for absolute deviations are shown. Values: *p* < 0.001 (***), *p* < 0.01 (**) and *p* < 0.05 (*)

C3’H is specifically involved in the transformation of *p-*coumaroyl CoA into caffeoyl CoA (a precursor of feruloyl CoA). The expression level of *C3’H* varies significantly between the both conditions and tissues (Fig. 10A). Interestingly, transcription of this gene becomes significantly high in Est-1 leaves, especially under in vitro conditions (*p* < 0.05). Col-0 is relatively low accumulator of *C3’H* transcript in roots. A next set of genes, *CCRs*, is directly related to lignin synthesis (Fig. 10B). *CCR1* is more highly expressed compared to *CCR2*, especially in leaf tissue. The expression patterns of both *CCR* genes is very similar in in vitro cultured roots, with significantly variable levels of *CC1* and *CC2* transcript accumulation among accessions. However, in roots grown in soil, these differences are small. *CCR1* transcription is higher in roots than in leaves of Est-1 and Tsu-1 accessions grown in vitro, while in Col-0 the ratio is reversed.

### Natural genomic variation

In order to further investigate the observed variations between Arabidopsis accessions and to better understand the possible correlation between the presence of coumarins/glycosylated coumarins and the expression level of various metabolic genes, we cloned and sequenced a set of coding sequences involved in the biosynthesis of phenylpropanoid molecules. For each gene (*F6’H1*,* F6’H2*,* 4CL*,* 4CL2*,* 4CL3*,* CCoAOMT1*,* C3’H* and *HCT*), except both *CCR* genes, two independent PCR products were cloned and sequenced separately. This sequencing task confirmed the presence of several SNPs that could be highlighted based on data available in the 1001 genomes database. Interestingly, we could also reveal some new SNPs, which are described below. Additionally, all detected SNPs of the tested Arabidopsis accessions along with possible resulting changes in amino acid sequences are presented in the Supplementary Information as Multiple sequence alignment (MSA) files from CLUSTALW (Supplementary Figure S3 and Figure S4). *4CL1* nucleotide sequence is highly conserved among the 3 studied accessions, Est-1 does not contain any SNPs, while Tsu-1 contains only one synonymous. *4CL2* does not show any polymorphisms in Tsu-1 whereas many SNPs could be detected in Est-1, especially on 5’ end of the gene although the sequence of the protein resulting from it is well conserved. Only one SNP led to a change in amino acids (Arg26Lys) but exchanged a positively charged side chain amino acid by a similar one. This SNP is popular in accessions from all regions from the POLYMORPH database (https://tools.1001genomes.org/polymorph/). We could not detect any polymorphism for *4CL3* in Tsu-1, but, again, numerous SNPs on the 5’end of the corresponding gene in Est-1. In this case, the SNPs led to amino acids substitutions (Thr15Ser, Asp22Gly, His27Pro, Asp43Asn, Tyr93Cys and Arg170Val). An additional significant insertion at the N-terminus of the protein sequence could also be highlighted leading to His27_Ser28insPro_Pro_Pro. Concerning the dioxygenases, Est-1 does not contain any SNPs in *F6’H1* coding sequence, but Tsu-1 has one which causes the amino acid substitution Pro70Lys, that can affect the secondary structure of the synthetized protein. This SNP is present in 1001 genomes database for Tsu-1 (http://1001genomes.org/data/MPI/MPIOssowski2008/releases/current/strains/Tsu-1/), but also in POLYMORPH database for one accession, named HKT2.4, from Tübingen region. Est-1 *F6’H2* sequencing revealed two SNPs in the coding region, which were not present in 1001 genome project database, leading to an arginine to proline substitution in Est-1. For *F6’H2* in Tsu-1 15 SNPs were found, all of which were described in 1001 genome project database. Interestingly, as many as six SNPs broadly existing in accessions from all regions represented in POLYMORPH database (from Central Asia, Caucasus, Europe and North Africa) changed amino acid sequence of Tsu-1 *F6’H2* (Ile7Met, Lys199Thr, Phe208Leu, Gly299Ser, Ser311Asn and Lys334Arg). Finally, *CCoAOMT1* both in Est-1 and Tsu-1 does not contain any SNPs. *C3’H* coding sequence in Tsu-1 contains one synonymous SNP. *HCT* in Est-1 has three polymorphisms while Tsu-1 has 5, but in both accessions two SNPs cause the same amino acid change from hydrophobic to polar group: Ala125Thr.

## Discussion

Here, additionally to scopolin, scopoletin and skimmin, umbelliferone was detected. We could not detect esculin in the investigated accessions as was detected by Kai et al. [10] and Perkowska et al. [16]. We detected a natural variation in accumulation of coumarins between Tsu-1, Col-0 and Est-1 accessions grown in vitro and in soil, together with variation in the gene expression between tested accessions. We verified SNPs present in the 1001 Genome database for Tsu-1 accession and found new SNPs and insertions in the Est-1 genetic background by re-sequencing.

Under non-stress conditions, coumarins are synthesized to a low extent. We observed only few significant differences in the production of coumarins in roots of tested accessions when grown in optimal soil mix (skimmin in Est-1 versus Tsu-1, *p* < 0.01). Biosynthesis of coumarins is induced by various biotic and abiotic elicitors as well as by stress factors such as in vitro liquid culture. This is consistent with the work of Hemm et al. [45], who have shown that the phenylpropanoid production increases in roots exposed to light, similarly to the liquid culture system we used. This may be caused by changes in gene expression due to activation of photoreceptors that monitor different wavelengths of light [47]. Coumarins act as UV screens, so the overproduction of these compounds in Arabidopsis roots in in vitro liquid culture may constitute a defense mechanism against radiation. Experimental conditions of in vitro liquid culture also promote plant vitrification - hyperhydric malformations that affect the physiological state of plants [48] by reducing the content of chlorophylls, carotenoids and lignin [49]. Vitrification and light exposure may alter the coumarin biosynthetic profile because it is tightly connected to lignin biosynthesis and may be genotype (accession)- dependent. In the roots of plants grown in vitro, we observed a decrease in the expression of the *4CL1* gene, which has the biggest contribution into lignin biosynthesis, and an increase in the *4CL2* expression. These changes may lead to an imbalance in the accumulation of lignin in the cell wall and an increase in the level of coumarins. The Col-0 accessions was characterized by the lowest level of *4CL1* and *4CL2* expression in the roots of in vitro cultured plants compared to Est-1 and Tsu-1. Genes with different expression levels between accessions may have polymorphisms in their promoter regions.

We found some SNPs in the eight coding sequences (CDS) of the investigated accessions by resequencing. In parallel, SNPs in the *CCR1* and *CCR2* genes in Tsu-1 were checked in the 1001 Genomes database. The *CCR1* gene in Tsu-1 genetic background does not contain any SNPs, while *CCR2* has four, of which two at each end of the protein are nonsynonymous - Leu2Pro and Ser332Pro. From the data obtained, it can be concluded that most of the nonsynonymous SNPs that might cause large changes in the enzyme structure occur in the CDS of enzyme homologues, which seem to be less important for homeostasis in non-stress conditions − 4CL3, F6’H2 and CCR2, but are crucial for the survival during stress. The coding sequences for 4CL1, CCoAOMT1 and C3’H are highly conserved. This is consistent with the definition of natural variability, i.e. adaptation to local habitats that may experience different weather or geographic conditions. Evolution is driven by the need of adaptation. Could some genomic sequences evolve faster? An intraspecific race for survival?

Natural variation for various traits among Arabidopsis accessions have been investigated in many studies [13, 50–52]. It has been reported that Arabidopsis display a great natural variation for the accumulation of secondary metabolites [53]. This is logical in the context of the evolutionary history of plants, which had to adapt to various biotic and abiotic stress factors to survive. In addition to developing mechanical barriers such as wood, cuticle, and thorns, plants have developed complex biochemical machinery to produce and release a huge variety of compounds displaying antimicrobial or antifungal properties. As numerous studies carried out on various Arabidopsis accessions collected in areas with different environmental conditions, altitudes, humidity, and salinity have shown, there is an enormous variation at the level of the genome [54, 55], transcriptome [56] and metabolome [13, 16, 57]. Importantly, natural variation of root exudates of 19 Arabidopsis accession was detected and a direct link between metabolic phenotypes and genotypes were shown without using segregating populations [58]. Our study suggests that the observed variability in metabolic phenotypes may be genetically determined, and the integration of genomics and metabolomics data along with the gene expression analysis might be useful in elucidating the biosynthetic pathway.

## Conclusion

Coumarins are secondary metabolites that have a range of important functions and biological activities valuable for both plants and humans. The unique structures of coumarins, make them useful in medicinal chemistry and pharmaceutical industry. In plants, they are involved in vital processes including adaptation to environmental stress factors, interactions with soil microorganisms and nutrient acquisition. Previously, we demonstrated for the first time that Arabidopsis with its extensive genetic variation and numerous publicly accessible web-based databases, is an exceptional model for studying molecular basis of natural variability underlying accumulation of coumarins in plants. Here, through targeted metabolic profiling and expression analysis of a set of phenylpropanoid genes, the gene-metabolite network was examined in more detail in the roots and leaves of selected three Arabidopsis accessions (Col-0, Est-1, Tsu-1) characterized by various levels of coumarin accumulation, which were grown in different types of cultures. We focused on two coumarin compounds, scopoletin and umbelliferone, along with their glycosides. This choice was dictated by the fact that scopoletin and scopolin are the main coumarins in Arabidopsis, and the biosynthesis of umbelliferone, recently discovered in this model plant by our research group, is completely unknown. We not only detected the expected variability in gene expression and coumarin accumulation among Arabidopsis grown in soil and in vitro cultures, but also found interesting polymorphisms in the coding sequences of the studied genes through in silico analysis and resequencing. Studying the natural variation in coumarin content present among Arabidopsis accessions followed by the analysis of various alleles possibly underlying the detected variation, may be useful in the future discovery of the physiological mechanisms of action of different alleles and better understanding the correlation between genetic and metabolic variants.

### Electronic supplementary material

Below is the link to the electronic supplementary material.

Additional file 1_Table S1. The gene-specific primers used for qPCRs

Additional file 2_Table S2. Efficiencies of the PCR product amplification by the qPCR primers used in this study

Additional file 3_Table S3. Primers sequences used for re-sequencing (gene-specific primers used for CDS amplification)

Additional file 4_ Figure S1. Skimmin and scopolin quantification in Arabidopsis roots grown in vitro

Additional file 5_ Figure S2. Umbelliferone and scopoletin quantification in Arabidopsis roots grown in vitro

Additional file 6_Figure S3. Nucleotide sequence variants shown as a Multiple sequence alignment (MSA)

Additional file 7_Figure S4. Amino acid sequence variants shown as a Multiple sequence alignment (MSA)

## Data Availability

All data supporting the findings of this study are available within the paper and its Supplementary Information. Gene-specific primer sequences used for qPCRs are provided in Supplementary Table S1, along with original reference describing the *ACTIN2* primer sequences used in this study. Efficiencies of the PCR product amplification by the qPCR primers are provided in Supplementary Table S2. Primer sequences used for re-sequencing are provided in Supplementary Table S3. Nucleotide sequences of the selected eight genes (*F6’H1, F6’H2, 4CL1, 4CL2, 4CL3, CCoAOMT1, C3’H* and *HCT*) of the studied Arabidopsis accessions are deposited in the GenBank ® repository (NIH genetic sequence database; https://www.ncbi.nlm.nih.gov/genbank/) with the relevant accession numbers from PP507124 to PP507144. Additionally, all sequence variants are provided as Multiple sequence alignment (MSA) files in Supplementary Figure S3 and S4. The effect of microwave treatment on the content of coumarins in MeOH extracts is provided in Supplementary Figures S1 and S2.

## Abbreviations

4CL1: 4-Coumarate: CoA Ligase 1
4CL2: 4-Coumarate: CoA Ligase 2
4CL3: 4-Coumarate: CoA Ligase 3
4CL4: 4-Coumarate: CoA Ligase 4
ACT2: ACTIN 2 *ACTIN 2*
C2’H: p-Coumaroyl CoA 2’-Hydroxylase
C3’H: p-Coumaroyl 3’-Hydroxylase
C4H: Trans-Cinnamate 4-monooxygenase
CCoAOMT1: Caffeoyl Coenzyme A dependent O-Methyltransferase 1
CCR1: Cinnamoyl CoA Reductase 1
CCR2: Cinnamoyl CoA Reductase 2
CoA: Coenzyme A
Col-0: Columbia
Est-1: Estland
F6’H1: Feruloyl-CoA 6’-Hydroxylase 1
F6’H2: Feruloyl-CoA 6’-Hydroxylase 2
HCT: Shikimate O-Hydroxycinnamoyltransferase
MD: Mean Deviation
N0: Initial copy number
PAL1: Phenylalanine Ammonia-Lyase 1
PAL2: Phenylalanine Ammonia-Lyase 2
PDR9: ABC transporter G family member 37
qPCR: Quantitative PCR
SNP: Single Nucloitide Polymorphism
Tsu-1: Tsushima
UHPLC: Ultra High Performance Liquid Chromatography
UV: Ultraviolet
ΔCp: Crossing Point
