# Supplementary material for: Genes to specialized metabolites: accumulation of scopoletin, umbelliferone and their glycosides in natural populations of Arabidopsis thaliana
Source: BMC Plant Biol. 2024 Aug 27;24:806. doi: 10.1186/s12870-024-05491-w (PMC11348552; doi:10.1186/s12870-024-05491-w)
Supplement: Supplementary file 1 — Additional file 1_Table S1. The gene-specific primers used for qPCRs [file 12870_2024_5491_MOESM1_ESM.docx]

Table S1. The gene-specific primers used for qPCRs.

| **Primer name** | **Primer sequence** |
| --- | --- |
| *ACTIN2 (ACT2)*  At3g18780 | 5′-CTT GCA CCA AGC AGC ATG AA-3′ and 3′-CCG ATC CAG ACA CTG TAC TTC CTT-5′ [40] |
| *F6’H1*  At3g13610 | 5′-TGA TGA GGA CAG AGT CGC TGA A-3′ and 5′-CAC TTG AAA GAA CCC CCA TTT C-3′ |
| *F6’H2*  At1g55290 | 5′-TCG GAC GTC ACT CTG ATG TTT C-3′ and 5′-GAG ACC ACC GAT CTC GTC TTG-3′ |
| *CCoAOMT1*  At4g34050 | 5′-GTT AAC GCC AAG AAC ACA ATG G-3′ and 5′-GCG GTG GCG AGA AGA GAG T-3′ |
| *C3’H*  At2g40890 | 5′-CCG CCG TCG TAT CCT ACA A-3′ and 5′-TGG GCC TGG TGG GAA CTT -3′ |
| *4CL1*  At1g51680 | 5′- CAA CCG GCC ACG TGT ACA C -3′ and 5′-GCG ATT TGG CGG GAG AT-3′ |
| *4CL2*  At3g21240 | 5′-CCC ACC GGC GAA GTA TAC AC-3′ and 5′- CGG CGA GTT TCC GAG ATG T-3′ |
| *4CL3*  At1g65060 | 5′-CCT TGG CCA GGG ATA TGG TA-3′ and 5′- ACC CAA GGC TCA TTG ACA ACA-3′ |
| *HCT*  At5g48930 | 5′-AGC TTA TTC CCG AAG TTG ATC ACT-3′ and 5′- CAA AAC GAG AAG CGG GAA AG-3′ |
| *CCR1*  At1g15950 | 5′-GCT CTT AAG GCG GCG ATT G-3′ and 5′- ACA GGA GAA GCC GTG TGA AAG-3′ |
| *CCR2*  At1g80820 | 5′-TCC CGA GAC AAT GTT GGA GC-3′ and 5′- CTT TAG CCG CTG CGT CAA TC-3′ |
