# Supplementary material for: Genes to specialized metabolites: accumulation of scopoletin, umbelliferone and their glycosides in natural populations of Arabidopsis thaliana
Source: BMC Plant Biol. 2024 Aug 27;24:806. doi: 10.1186/s12870-024-05491-w (PMC11348552; doi:10.1186/s12870-024-05491-w)
Supplement: Supplementary file 2 — Additional file 2_Table S2. Efficiencies of the PCR product amplification by the qPCR primers used in this study [file 12870_2024_5491_MOESM2_ESM.docx]

**Table S2.** Efficiencies of the PCR product amplification by the qPCR primers used.

| **Gene name, ID number** | **E values** |
| --- | --- |
| *ACTIN2 (ACT2)* At3g18780 | 1.93* [40] |
| *F6’H1* At3g13610 | 1.98 |
| *F6’H2* At1g55290 | 2.04 |
| *CCoAOMT1* At4g34050 | 1.99 |
| *C3’H* At2g40890 | 1.97 |
| *4Cl1* At1g51680 | 1.95 |
| *4Cl2* At3g21240 | 1.98 |
| *4Cl3* At1g65060 | 1.97 |
| *HCT* At5g48930 | 1.98 |
| *CCR1* At1g15950 | 1.88 |
| *CCR2* At1g80820 | 1.93 |
