# Supplementary material for: Genes to specialized metabolites: accumulation of scopoletin, umbelliferone and their glycosides in natural populations of Arabidopsis thaliana
Source: BMC Plant Biol. 2024 Aug 27;24:806. doi: 10.1186/s12870-024-05491-w (PMC11348552; doi:10.1186/s12870-024-05491-w)
Supplement: Supplementary file 3 — Additional file 3_Table S3. Primers sequences used for re-sequencing (gene-specific primers used for CDS amplification) [file 12870_2024_5491_MOESM3_ESM.docx]

Table S3. Primers sequences used for re-sequencing (gene-specific primers used for CDS amplification).

| **Primer name** | **Primer sequence** |
| --- | --- |
| *F6’H1*  At3g13610 | 5′-CCA TGG CTC CAA CAC TCT TGA CAA CCC A-3′ and 5′-CTC GAG GAT CTT GGC GTA ATC GAC GGT TTT C-3′ |
| *F6’H2*  At1g55290 | 5′-GGA TCC ATG AAT CAA ACA CTC GCT GCC CAA TTC TTA AC-3′ and 5′-AAG CTT TCA GTG GTG GTG GTG GTG GTG AAT GTT GGC AAA ATC GAT GGT TTT CTT CC-3′ |
| *C3’H*  At2g40890 | 5’-GGA TCC ATG TCG TGG TTT CTA ATA GCG GTG GC-3’ and 5’-GAA TTC TCA GTG GTG GTG GTG GTG GTG CAT ATC GTA AGG CAC GCG TTT GTA C-3’ |
| *CCoAOMT1*  At4g34050 | 5’-ATG GCG ACG ACA ACA ACA GAA-3’ and 5’-TCA ACT GAT CCG ACG GCA-3’ |
| *4CL1*  At1g51680 | 5’-ATG GCG CCA CAA GAA CAA GC-3’ and 5’-TCA CAA TCC ATT TGC TAG TTT TGC C-3’ |
| *4CL2*  At3g21240 | 5’-GGG GCG GAT CCA CGA CAC AAG ATG TGA TAG TCA ATG-3’ and 5’-GGG GCC CAT GGC TAG TTC ATT AAT CCA TTT GCT AGT CTT G-3’ |
| *4CL3*  At1g65060 | 5’-GGA TCC ATG ATC ACT GCA GCT CTA CAC GAA CC-3’ and 5’-GGG GCC CAT GGT CAA CAA AGC TTA GCT TTG AGG TCC TTT C-3’ |
| *HCT*  At5g48930 | 5’-ATG AAA ATT AAC ATC AGA GAT TCC A-3’ and 5’- TCA TAT CTC AAA CAA AAA CTT CTC AAA C-3’ |
