# Supplementary material for: Genes to specialized metabolites: accumulation of scopoletin, umbelliferone and their glycosides in natural populations of Arabidopsis thaliana
Source: BMC Plant Biol. 2024 Aug 27;24:806. doi: 10.1186/s12870-024-05491-w (PMC11348552; doi:10.1186/s12870-024-05491-w)
Supplement: Supplementary file 5 — Additional file 5_ Figure S2. Umbelliferone and scopoletin quantification in Arabidopsis roots grown in vitro [file 12870_2024_5491_MOESM5_ESM.docx]

**Figure S2. Umbelliferone and scopoletin quantification in Arabidopsis roots grown *in vitro***. (H+) with enzymatic hydrolysis treatment, (M-) without microwave treatment, (M+) with microwave treatment. Values: means and standard deviations.
