# Supplementary material for: Genes to specialized metabolites: accumulation of scopoletin, umbelliferone and their glycosides in natural populations of Arabidopsis thaliana
Source: BMC Plant Biol. 2024 Aug 27;24:806. doi: 10.1186/s12870-024-05491-w (PMC11348552; doi:10.1186/s12870-024-05491-w)
Supplement: Supplementary file 6 — Additional file 6_Figure S3. Nucleotide sequence variants shown as a Multiple sequence alignment (MSA) [file 12870_2024_5491_MOESM6_ESM.docx]

**Figure S3. Multiple Sequence Alignment (MSA) of tested genes’ coding sequences.** To illustrate conserved and variable regions in selected coding sequences from Col-0, Est-1, and Tsu-1 accessions, nucleotide sequences were aligned using the ClustalW algorithm. Grey highlighting indicates similar nucleotides across the aligned sequences. Black highlighting marks the positions of different nucleotides. MSA was created and visualized using BioEdit v5.0.9 software.

**4-coumarate CoA ligase 1 (4CL1, At1g51680)**

10 20 30 40 50 60 70 80 90 100

....|....|....|....|....|....|....|....|....|....|....|....|....|....|....|....|....|....|....|....|

**4CL1_Col-0** 1 ATGGCGCCACAAGAACAAGCAGTTTCTCAGGTGATGGAGAAACAGAGCAACAACAACAACAGTGACGTCATTTTCCGATCAAAGTTACCGGATATTTACA

**4CL1_Est-1** 1 ATGGCGCCACAAGAACAAGCAGTTTCTCAGGTGATGGAGAAACAGAGCAACAACAACAACAGTGACGTCATTTTCCGATCAAAGTTACCGGATATTTACA

**4CL1_Tsu-1** 1 ATGGCGCCACAAGAACAAGCAGTTTCTCAGGTGATGGAGAAACAGAGCAACAACAACAACAGTGACGTCATTTTCCGATCAAAGTTACCGGATATTTACA

110 120 130 140 150 160 170 180 190 200

....|....|....|....|....|....|....|....|....|....|....|....|....|....|....|....|....|....|....|....|

**4CL1_Col-0** 101 TCCCGAACCACCTATCTCTCCACGACTACATCTTCCAAAACATCTCCGAATTCGCCACTAAGCCTTGCCTAATCAACGGACCAACCGGCCACGTGTACAC

**4CL1_Est-1** 101 TCCCGAACCACCTATCTCTCCACGACTACATCTTCCAAAACATCTCCGAATTCGCCACTAAGCCTTGCCTAATCAACGGACCAACCGGCCACGTGTACAC

**4CL1_Tsu-1** 101 TCCCGAACCACCTATCTCTCCACGACTACATCTTCCAAAACATCTCCGAATTCGCCACTAAGCCTTGCCTAATCAACGGACCAACCGGCCACGTGTACAC

210 220 230 240 250 260 270 280 290 300

....|....|....|....|....|....|....|....|....|....|....|....|....|....|....|....|....|....|....|....|

**4CL1_Col-0** 201 TTACTCCGACGTCCACGTCATCTCCCGCCAAATCGCCGCCAATTTTCACAAACTCGGCGTTAACCAAAACGACGTCGTCATGCTCCTCCTCCCAAACTGT

**4CL1_Est-1** 201 TTACTCCGACGTCCACGTCATCTCCCGCCAAATCGCCGCCAATTTTCACAAACTCGGCGTTAACCAAAACGACGTCGTCATGCTCCTCCTCCCAAACTGT

**4CL1_Tsu-1** 201 TTACTCCGACGTCCACGTCATCTCCCGCCAAATCGCCGCCAATTTTCACAAACTCGGCGTTAACCAAAACGACGTCGTCATGCTCCTCCTCCCAAACTGT

310 320 330 340 350 360 370 380 390 400

....|....|....|....|....|....|....|....|....|....|....|....|....|....|....|....|....|....|....|....|

**4CL1_Col-0** 301 CCCGAATTCGTCCTCTCTTTCCTCGCCGCCTCCTTCCGCGGCGCAACCGCCACCGCCGCAAACCCTTTCTTCACTCCGGCGGAGATAGCTAAACAAGCCA

**4CL1_Est-1** 301 CCCGAATTCGTCCTCTCTTTCCTCGCCGCCTCCTTCCGCGGCGCAACCGCCACCGCCGCAAACCCTTTCTTCACTCCGGCGGAGATAGCTAAACAAGCCA

**4CL1_Tsu-1** 301 CCCGAATTCGTCCTCTCTTTCCTCGCCGCCTCTTTCCGCGGCGCAACCGCCACCGCCGCAAACCCTTTCTTCACTCCGGCGGAGATAGCTAAACAAGCCA

410 420 430 440 450 460 470 480 490 500

....|....|....|....|....|....|....|....|....|....|....|....|....|....|....|....|....|....|....|....|

**4CL1_Col-0** 401 AAGCCTCCAACACCAAACTCATAATCACCGAAGCTCGTTACGTCGACAAAATCAAACCACTTCAAAACGACGACGGAGTAGTCATCGTCTGCATCGACGA

**4CL1_Est-1** 401 AAGCCTCCAACACCAAACTCATAATCACCGAAGCTCGTTACGTCGACAAAATCAAACCACTTCAAAACGACGACGGAGTAGTCATCGTCTGCATCGACGA

**4CL1_Tsu-1** 401 AAGCCTCCAACACCAAACTCATAATCACCGAAGCTCGTTACGTCGACAAAATCAAACCACTTCAAAACGACGACGGAGTAGTCATCGTCTGCATCGACGA

510 520 530 540 550 560 570 580 590 600

....|....|....|....|....|....|....|....|....|....|....|....|....|....|....|....|....|....|....|....|

**4CL1_Col-0** 501 CAACGAATCCGTGCCAATCCCTGAAGGCTGCCTCCGCTTCACCGAGTTGACTCAGTCGACAACCGAGGCATCAGAAGTCATCGACTCGGTGGAGATTTCA

**4CL1_Est-1** 501 CAACGAATCCGTGCCAATCCCTGAAGGCTGCCTCCGCTTCACCGAGTTGACTCAGTCGACAACCGAGGCATCAGAAGTCATCGACTCGGTGGAGATTTCA

**4CL1_Tsu-1** 501 CAACGAATCCGTGCCAATCCCTGAAGGCTGCCTCCGCTTCACCGAGTTGACTCAGTCGACAACCGAGGCATCAGAAGTCATCGACTCGGTGGAGATTTCA

610 620 630 640 650 660 670 680 690 700

....|....|....|....|....|....|....|....|....|....|....|....|....|....|....|....|....|....|....|....|

**4CL1_Col-0** 601 CCGGACGACGTGGTGGCACTACCTTACTCCTCTGGCACGACGGGATTACCAAAAGGAGTGATGCTGACTCACAAGGGACTAGTCACGAGCGTTGCTCAGC

**4CL1_Est-1** 601 CCGGACGACGTGGTGGCACTACCTTACTCCTCTGGCACGACGGGATTACCAAAAGGAGTGATGCTGACTCACAAGGGACTAGTCACGAGCGTTGCTCAGC

**4CL1_Tsu-1** 601 CCGGACGACGTGGTGGCACTACCTTACTCCTCTGGCACGACGGGATTACCAAAAGGAGTGATGCTGACTCACAAGGGACTAGTCACGAGCGTTGCTCAGC

710 720 730 740 750 760 770 780 790 800

....|....|....|....|....|....|....|....|....|....|....|....|....|....|....|....|....|....|....|....|

**4CL1_Col-0** 701 AAGTCGACGGCGAGAACCCGAATCTTTATTTCCACAGCGATGACGTCATACTCTGTGTTTTGCCCATGTTTCATATCTACGCTTTGAACTCGATCATGTT

**4CL1_Est-1** 701 AAGTCGACGGCGAGAACCCGAATCTTTATTTCCACAGCGATGACGTCATACTCTGTGTTTTGCCCATGTTTCATATCTACGCTTTGAACTCGATCATGTT

**4CL1_Tsu-1** 701 AAGTCGACGGCGAGAACCCGAATCTTTATTTCCACAGCGATGACGTCATACTCTGTGTTTTGCCCATGTTTCATATCTACGCTTTGAACTCGATCATGTT

810 820 830 840 850 860 870 880 890 900

....|....|....|....|....|....|....|....|....|....|....|....|....|....|....|....|....|....|....|....|

**4CL1_Col-0** 801 GTGTGGTCTTAGAGTTGGTGCGGCGATTCTGATAATGCCGAAGTTTGAGATCAATCTGCTATTGGAGCTGATCCAGAGGTGTAAAGTGACGGTGGCTCCG

**4CL1_Est-1** 801 GTGTGGTCTTAGAGTTGGTGCGGCGATTCTGATAATGCCGAAGTTTGAGATCAATCTGCTATTGGAGCTGATCCAGAGGTGTAAAGTGACGGTGGCTCCG

**4CL1_Tsu-1** 801 GTGTGGTCTTAGAGTTGGTGCGGCGATTCTGATAATGCCGAAGTTTGAGATCAATCTGCTATTGGAGCTGATCCAGAGGTGTAAAGTGACGGTGGCTCCG

910 920 930 940 950 960 970 980 990 1000

....|....|....|....|....|....|....|....|....|....|....|....|....|....|....|....|....|....|....|....|

**4CL1_Col-0** 901 ATGGTTCCGCCGATTGTGTTGGCCATTGCGAAGTCTTCGGAGACGGAGAAGTATGATTTGAGCTCGATAAGAGTGGTGAAATCTGGTGCTGCTCCTCTTG

**4CL1_Est-1** 901 ATGGTTCCGCCGATTGTGTTGGCCATTGCGAAGTCTTCGGAGACGGAGAAGTATGATTTGAGCTCGATAAGAGTGGTGAAATCTGGTGCTGCTCCTCTTG

**4CL1_Tsu-1** 901 ATGGTTCCGCCGATTGTGTTGGCCATTGCGAAGTCTTCGGAGACGGAGAAGTATGATTTGAGCTCGATAAGAGTGGTGAAATCTGGTGCTGCTCCTCTTG

1010 1020 1030 1040 1050 1060 1070 1080 1090 1100

....|....|....|....|....|....|....|....|....|....|....|....|....|....|....|....|....|....|....|....|

**4CL1_Col-0** 1001 GTAAAGAACTTGAAGATGCCGTTAATGCCAAGTTTCCTAATGCCAAACTCGGTCAGGGATACGGAATGACGGAAGCAGGTCCAGTGCTAGCAATGTCGTT

**4CL1_Est-1** 1001 GTAAAGAACTTGAAGATGCCGTTAATGCCAAGTTTCCTAATGCCAAACTCGGTCAGGGATACGGAATGACGGAAGCAGGTCCAGTGCTAGCAATGTCGTT

**4CL1_Tsu-1** 1001 GTAAAGAACTTGAAGATGCCGTTAATGCCAAGTTTCCTAATGCCAAACTCGGTCAGGGATACGGAATGACGGAAGCAGGTCCAGTGCTAGCAATGTCGTT

1110 1120 1130 1140 1150 1160 1170 1180 1190 1200

....|....|....|....|....|....|....|....|....|....|....|....|....|....|....|....|....|....|....|....|

**4CL1_Col-0** 1101 AGGTTTTGCAAAGGAACCTTTTCCGGTTAAGTCAGGAGCTTGTGGTACTGTTGTAAGAAATGCTGAGATGAAAATAGTTGATCCAGACACCGGAGATTCT

**4CL1_Est-1** 1101 AGGTTTTGCAAAGGAACCTTTTCCGGTTAAGTCAGGAGCTTGTGGTACTGTTGTAAGAAATGCTGAGATGAAAATAGTTGATCCAGACACCGGAGATTCT

**4CL1_Tsu-1** 1101 AGGTTTTGCAAAGGAACCTTTTCCGGTTAAGTCAGGAGCTTGTGGTACTGTTGTAAGAAATGCTGAGATGAAAATAGTTGATCCAGACACCGGAGATTCT

1210 1220 1230 1240 1250 1260 1270 1280 1290 1300

....|....|....|....|....|....|....|....|....|....|....|....|....|....|....|....|....|....|....|....|

**4CL1_Col-0** 1201 CTTTCGAGGAATCAACCCGGTGAGATTTGTATTCGTGGTCACCAGATCATGAAAGGTTACCTCAACAATCCGGCAGCTACAGCAGAGACCATTGATAAAG

**4CL1_Est-1** 1201 CTTTCGAGGAATCAACCCGGTGAGATTTGTATTCGTGGTCACCAGATCATGAAAGGTTACCTCAACAATCCGGCAGCTACAGCAGAGACCATTGATAAAG

**4CL1_Tsu-1** 1201 CTTTCGAGGAATCAACCCGGTGAGATTTGTATTCGTGGTCACCAGATCATGAAAGGTTACCTCAACAATCCGGCAGCTACAGCAGAGACCATTGATAAAG

1310 1320 1330 1340 1350 1360 1370 1380 1390 1400

....|....|....|....|....|....|....|....|....|....|....|....|....|....|....|....|....|....|....|....|

**4CL1_Col-0** 1301 ACGGTTGGCTTCATACTGGAGATATTGGATTGATCGATGACGATGACGAGCTTTTCATCGTTGATCGATTGAAAGAACTTATCAAGTATAAAGGTTTTCA

**4CL1_Est-1** 1301 ACGGTTGGCTTCATACTGGAGATATTGGATTGATCGATGACGATGACGAGCTTTTCATCGTTGATCGATTGAAAGAACTTATCAAGTATAAAGGTTTTCA

**4CL1_Tsu-1** 1301 ACGGTTGGCTTCATACTGGAGATATTGGATTGATCGATGACGATGACGAGCTTTTCATCGTTGATCGATTGAAAGAACTTATCAAGTATAAAGGTTTTCA

1410 1420 1430 1440 1450 1460 1470 1480 1490 1500

....|....|....|....|....|....|....|....|....|....|....|....|....|....|....|....|....|....|....|....|

**4CL1_Col-0** 1401 GGTAGCTCCGGCTGAGCTAGAGGCTTTGCTCATCGGTCATCCTGACATTACTGATGTTGCTGTTGTCGCAATGAAAGAAGAAGCAGCTGGTGAAGTTCCT

**4CL1_Est-1** 1401 GGTAGCTCCGGCTGAGCTAGAGGCTTTGCTCATCGGTCATCCTGACATTACTGATGTTGCTGTTGTCGCAATGAAAGAAGAAGCAGCTGGTGAAGTTCCT

**4CL1_Tsu-1** 1401 GGTAGCTCCGGCTGAGCTAGAGGCTTTGCTCATCGGTCATCCTGACATTACTGATGTTGCTGTTGTCGCAATGAAAGAAGAAGCAGCTGGTGAAGTTCCT

1510 1520 1530 1540 1550 1560 1570 1580 1590 1600

....|....|....|....|....|....|....|....|....|....|....|....|....|....|....|....|....|....|....|....|

**4CL1_Col-0** 1501 GTTGCATTTGTGGTGAAATCGAAGGATTCGGAGTTATCAGAAGATGATGTGAAGCAATTCGTGTCGAAACAGGTTGTGTTTTACAAGAGAATCAACAAAG

**4CL1_Est-1** 1501 GTTGCATTTGTGGTGAAATCGAAGGATTCGGAGTTATCAGAAGATGATGTGAAGCAATTCGTGTCGAAACAGGTTGTGTTTTACAAGAGAATCAACAAAG

**4CL1_Tsu-1** 1501 GTTGCATTTGTGGTGAAATCGAAGGATTCGGAGTTATCAGAAGATGATGTGAAGCAATTCGTGTCGAAACAGGTTGTGTTTTACAAGAGAATCAACAAAG

1610 1620 1630 1640 1650 1660 1670 1680

....|....|....|....|....|....|....|....|....|....|....|....|....|....|....|....|....|.

**4CL1_Col-0** 1601 TGTTCTTCACTGAATCCATTCCTAAAGCTCCATCAGGGAAGATATTGAGGAAAGATCTGAGGGCAAAACTAGCAAATGGATTGTGA

**4CL1_Est-1** 1601 TGTTCTTCACTGAATCCATTCCTAAAGCTCCATCAGGGAAGATATTGAGGAAAGATCTGAGGGCAAAACTAGCAAATGGATTGTGA

**4CL1_Tsu-1** 1601 TGTTCTTCACTGAATCCATTCCTAAAGCTCCATCAGGGAAGATATTGAGGAAAGATCTGAGGGCAAAACTAGCAAATGGATTGTGA

**4-coumarate CoA ligase 2 (4CL2, At3g21240)**

10 20 30 40 50 60 70 80 90 100

....|....|....|....|....|....|....|....|....|....|....|....|....|....|....|....|....|....|....|....|

**4CL2_Col-0** 1 ATGACGACACAAGATGTGATAGTCAATGATCAGAATGATCAGAAACAGTGTAGTAATGACGTCATTTTCCGATCGAGATTGCCTGATATATACATCCCTA

**4CL2_Est-1** 1 ATGACGACACAAGATGTGATAGTCAATGATCAGAATGATCAGAAACAGTGTAGTAATGACGTCATTTTCCGATCTAAATTGCCTGATATTTACATCCCTA

**4CL2_Tsu-1** 1 ATGACGACACAAGATGTGATAGTCAATGATCAGAATGATCAGAAACAGTGTAGTAATGACGTCATTTTCCGATCGAGATTGCCTGATATATACATCCCTA

110 120 130 140 150 160 170 180 190 200

....|....|....|....|....|....|....|....|....|....|....|....|....|....|....|....|....|....|....|....|

**4CL2_Col-0** 101 ACCACCTCCCACTCCACGACTACATCTTCGAAAATATCTCAGAGTTCGCCGCTAAGCCATGCTTGATCAACGGTCCCACCGGCGAAGTATACACCTACGC

**4CL2_Est-1** 101 ACCACCTCCCACTCCACGACTACATCTTCGAAAACATCTCTGAGTTCGCCGCTAAACCATGTTTGATCAACGGTCCCACCGGCGAAGTATACACCTACGC

**4CL2_Tsu-1** 101 ACCACCTCCCACTCCACGACTACATCTTCGAAAATATCTCAGAGTTCGCCGCTAAGCCATGCTTGATCAACGGTCCCACCGGCGAAGTATACACCTACGC

210 220 230 240 250 260 270 280 290 300

....|....|....|....|....|....|....|....|....|....|....|....|....|....|....|....|....|....|....|....|

**4CL2_Col-0** 201 CGATGTCCACGTAACATCTCGGAAACTCGCCGCCGGTCTTCATAACCTCGGCGTGAAGCAACACGACGTTGTAATGATCCTCCTCCCGAACTCTCCTGAA

**4CL2_Est-1** 201 CGATGTCCACGTAACATCTCGGAAACTCGCCGCCGGTCTTCATAACCTCGGCGTGAAGCAACACGACGTTGTAATGATCCTCCTCCCGAACTCTCCTGAA

**4CL2_Tsu-1** 201 CGATGTCCACGTAACATCTCGGAAACTCGCCGCCGGTCTTCATAACCTCGGCGTGAAGCAACACGACGTTGTAATGATCCTCCTCCCGAACTCTCCTGAA

310 320 330 340 350 360 370 380 390 400

....|....|....|....|....|....|....|....|....|....|....|....|....|....|....|....|....|....|....|....|

**4CL2_Col-0** 301 GTAGTCCTCACTTTCCTTGCCGCCTCCTTCATCGGCGCAATCACCACCTCCGCGAACCCGTTCTTCACTCCGGCGGAGATTTCTAAACAAGCCAAAGCCT

**4CL2_Est-1** 301 GTAGTCCTCACTTTCCTTGCCGCCTCCTTCATCGGCGCAATCACCACCTCCGCGAACCCGTTCTTCACTCCGGCGGAGATTTCTAAACAAGCCAAAGCCT

**4CL2_Tsu-1** 301 GTAGTCCTCACTTTCCTTGCCGCCTCCTTCATCGGCGCAATCACCACCTCCGCGAACCCGTTCTTCACTCCGGCGGAGATTTCTAAACAAGCCAAAGCCT

410 420 430 440 450 460 470 480 490 500

....|....|....|....|....|....|....|....|....|....|....|....|....|....|....|....|....|....|....|....|

**4CL2_Col-0** 401 CCGCGGCGAAACTCATCGTCACTCAATCCCGTTACGTCGATAAAATCAAGAACCTCCAAAACGACGGCGTTTTGATCGTCACCACCGACTCCGACGCCAT

**4CL2_Est-1** 401 CCGCGGCGAAACTCATCGTCACTCAATCCCGTTACGTCGATAAAATCAAGAACCTCCAAAACGACGGCGTTTTGATCGTCACCACCGACTCCGACGCCAT

**4CL2_Tsu-1** 401 CCGCGGCGAAACTCATCGTCACTCAATCCCGTTACGTCGATAAAATCAAGAACCTCCAAAACGACGGCGTTTTGATCGTCACCACCGACTCCGACGCCAT

510 520 530 540 550 560 570 580 590 600

....|....|....|....|....|....|....|....|....|....|....|....|....|....|....|....|....|....|....|....|

**4CL2_Col-0** 501 CCCCGAAAACTGCCTCCGTTTCTCCGAGTTAACTCAGTCCGAAGAACCACGAGTGGACTCAATACCGGAGAAGATTTCGCCAGAAGACGTCGTGGCGCTT

**4CL2_Est-1** 501 CCCCGAAAACTGCCTCCGTTTCTCCGAGTTAACTCAGTCCGAAGAACCACGAGTGGACTCAATACCGGAGAAGATTTCGCCAGAAGACGTCGTGGCGCTT

**4CL2_Tsu-1** 501 CCCCGAAAACTGCCTCCGTTTCTCCGAGTTAACTCAGTCCGAAGAACCACGAGTGGACTCAATACCGGAGAAGATTTCGCCAGAAGACGTCGTGGCGCTT

610 620 630 640 650 660 670 680 690 700

....|....|....|....|....|....|....|....|....|....|....|....|....|....|....|....|....|....|....|....|

**4CL2_Col-0** 601 CCTTTCTCATCCGGCACGACGGGTCTCCCCAAAGGAGTGATGCTAACACACAAAGGTCTAGTCACGAGCGTGGCGCAGCAAGTCGACGGCGAGAATCCGA

**4CL2_Est-1** 601 CCTTTCTCATCCGGCACGACGGGTCTCCCCAAAGGAGTGATGCTAACACACAAAGGTCTAGTCACGAGCGTGGCGCAGCAAGTCGACGGCGAGAATCCGA

**4CL2_Tsu-1** 601 CCTTTCTCATCCGGCACGACGGGTCTCCCCAAAGGAGTGATGCTAACACACAAAGGTCTAGTCACGAGCGTGGCGCAGCAAGTCGACGGCGAGAATCCGA

710 720 730 740 750 760 770 780 790 800

....|....|....|....|....|....|....|....|....|....|....|....|....|....|....|....|....|....|....|....|

**4CL2_Col-0** 701 ATCTTTACTTCAACAGAGACGACGTGATCCTCTGTGTCTTGCCTATGTTCCATATATACGCTCTCAACTCCATCATGCTCTGTAGTCTCAGAGTTGGTGC

**4CL2_Est-1** 701 ATCTTTACTTCAACAGAGACGACGTGATCCTCTGTGTCTTGCCTATGTTCCATATATACGCTCTCAACTCCATCATGCTCTGTAGTCTCAGAGTTGGTGC

**4CL2_Tsu-1** 701 ATCTTTACTTCAACAGAGACGACGTGATCCTCTGTGTCTTGCCTATGTTCCATATATACGCTCTCAACTCCATCATGCTCTGTAGTCTCAGAGTTGGTGC

810 820 830 840 850 860 870 880 890 900

....|....|....|....|....|....|....|....|....|....|....|....|....|....|....|....|....|....|....|....|

**4CL2_Col-0** 801 CACGATCTTGATAATGCCTAAGTTCGAAATCACTCTCTTGTTAGAGCAGATACAAAGGTGTAAAGTCACGGTGGCTATGGTCGTGCCACCGATCGTTTTA

**4CL2_Est-1** 801 CACGATCTTGATAATGCCTAAGTTCGAAATCACTCTCTTGTTAGAGCAGATACAAAGGTGTAAAGTCACGGTGGCTATGGTCGTGCCACCGATCGTTTTA

**4CL2_Tsu-1** 801 CACGATCTTGATAATGCCTAAGTTCGAAATCACTCTCTTGTTAGAGCAGATACAAAGGTGTAAAGTCACGGTGGCTATGGTCGTGCCACCGATCGTTTTA

910 920 930 940 950 960 970 980 990 1000

....|....|....|....|....|....|....|....|....|....|....|....|....|....|....|....|....|....|....|....|

**4CL2_Col-0** 901 GCTATCGCGAAGTCGCCGGAGACGGAGAAGTATGATCTGAGCTCGGTTAGGATGGTTAAGTCTGGAGCAGCTCCTCTTGGTAAGGAGCTTGAAGATGCTA

**4CL2_Est-1** 901 GCTATCGCGAAGTCGCCGGAGACGGAGAAGTATGATCTGAGCTCGGTTAGGATGGTTAAGTCTGGAGCAGCTCCTCTTGGTAAGGAGCTAGAAGATGCTA

**4CL2_Tsu-1** 901 GCTATCGCGAAGTCGCCGGAGACGGAGAAGTATGATCTGAGCTCGGTTAGGATGGTTAAGTCTGGAGCAGCTCCTCTTGGTAAGGAGCTTGAAGATGCTA

1010 1020 1030 1040 1050 1060 1070 1080 1090 1100

....|....|....|....|....|....|....|....|....|....|....|....|....|....|....|....|....|....|....|....|

**4CL2_Col-0** 1001 TTAGTGCTAAGTTTCCTAACGCCAAGCTTGGTCAGGGCTATGGGATGACAGAAGCAGGTCCGGTGCTAGCAATGTCGTTAGGGTTTGCTAAAGAGCCGTT

**4CL2_Est-1** 1001 TTAGTGCTAAGTTTCCTAACGCCAAGCTTGGTCAGGGCTATGGGATGACAGAAGCAGGTCCGGTGCTAGCAATGTCGTTAGGGTTTGCTAAAGAGCCGTT

**4CL2_Tsu-1** 1001 TTAGTGCTAAGTTTCCTAACGCCAAGCTTGGTCAGGGCTATGGGATGACAGAAGCAGGTCCGGTGCTAGCAATGTCGTTAGGGTTTGCTAAAGAGCCGTT

1110 1120 1130 1140 1150 1160 1170 1180 1190 1200

....|....|....|....|....|....|....|....|....|....|....|....|....|....|....|....|....|....|....|....|

**4CL2_Col-0** 1101 TCCAGTGAAGTCAGGAGCATGTGGTACGGTGGTGAGGAACGCCGAGATGAAGATACTTGATCCAGACACAGGAGATTCTTTGCCTAGGAACAAACCCGGC

**4CL2_Est-1** 1101 TCCAGTGAAGTCAGGAGCATGTGGTACGGTGGTGAGGAACGCCGAGATGAAGATACTTGATCCAGACACAGGAGATTCTTTGCCTAGGAACAAACCCGGC

**4CL2_Tsu-1** 1101 TCCAGTGAAGTCAGGAGCATGTGGTACGGTGGTGAGGAACGCCGAGATGAAGATACTTGATCCAGACACAGGAGATTCTTTGCCTAGGAACAAACCCGGC

1210 1220 1230 1240 1250 1260 1270 1280 1290 1300

....|....|....|....|....|....|....|....|....|....|....|....|....|....|....|....|....|....|....|....|

**4CL2_Col-0** 1201 GAAATATGCATCCGTGGCAACCAAATCATGAAAGGCTATCTCAATGACCCCTTGGCCACGGCATCGACGATCGATAAAGATGGTTGGCTTCACACTGGAG

**4CL2_Est-1** 1201 GAAATATGCATCCGTGGCAACCAAATCATGAAAGGCTATCTCAATGACCCCTTGGCCACGGCATCGACGATCGATAAAGATGGTTGGCTTCACACTGGAG

**4CL2_Tsu-1** 1201 GAAATATGCATCCGTGGCAACCAAATCATGAAAGGCTATCTCAATGACCCCTTGGCCACGGCATCGACGATCGATAAAGATGGTTGGCTTCACACTGGAG

1310 1320 1330 1340 1350 1360 1370 1380 1390 1400

....|....|....|....|....|....|....|....|....|....|....|....|....|....|....|....|....|....|....|....|

**4CL2_Col-0** 1301 ACGTCGGATTTATCGATGATGACGACGAGCTTTTCATTGTGGATAGATTGAAAGAACTCATCAAGTACAAAGGATTTCAAGTGGCTCCAGCTGAGCTAGA

**4CL2_Est-1** 1301 ACGTCGGATTTATCGATGATGACGACGAGCTTTTCATTGTGGATAGATTGAAAGAACTCATCAAGTACAAAGGATTTCAAGTGGCTCCAGCTGAGCTAGA

**4CL2_Tsu-1** 1301 ACGTCGGATTTATCGATGATGACGACGAGCTTTTCATTGTGGATAGATTGAAAGAACTCATCAAGTACAAAGGATTTCAAGTGGCTCCAGCTGAGCTAGA

1410 1420 1430 1440 1450 1460 1470 1480 1490 1500

....|....|....|....|....|....|....|....|....|....|....|....|....|....|....|....|....|....|....|....|

**4CL2_Col-0** 1401 GTCTCTCCTCATAGGTCATCCAGAAATCAATGATGTTGCTGTCGTCGCCATGAAGGAAGAAGATGCTGGTGAGGTTCCTGTTGCGTTTGTGGTGAGATCG

**4CL2_Est-1** 1401 GTCTCTCCTCATAGGTCATCCAGAAATCAATGATGTTGCTGTCGTCGCCATGAAGGAAGAAGATGCTGGTGAGGTTCCTGTTGCGTTTGTGGTGAGATCG

**4CL2_Tsu-1** 1401 GTCTCTCCTCATAGGTCATCCAGAAATCAATGATGTTGCTGTCGTCGCCATGAAGGAAGAAGATGCTGGTGAGGTTCCTGTTGCGTTTGTGGTGAGATCG

1510 1520 1530 1540 1550 1560 1570 1580 1590 1600

....|....|....|....|....|....|....|....|....|....|....|....|....|....|....|....|....|....|....|....|

**4CL2_Col-0** 1501 AAAGATTCAAATATATCCGAAGATGAAATCAAGCAATTCGTGTCAAAACAGGTTGTGTTTTATAAGAGAATCAACAAAGTGTTCTTCACTGACTCTATTC

**4CL2_Est-1** 1501 AAAGATTCAAATATATCCGAAGATGAAATCAAGCAATTCGTGTCAAAACAGGTTGTGTTTTATAAGAGAATCAACAAAGTGTTCTTCACTGACTCTATTC

**4CL2_Tsu-1** 1501 AAAGATTCAAATATATCCGAAGATGAAATCAAGCAATTCGTGTCAAAACAGGTTGTGTTTTATAAGAGAATCAACAAAGTGTTCTTCACTGACTCTATTC

1610 1620 1630 1640 1650 1660 1670

....|....|....|....|....|....|....|....|....|....|....|....|....|....|.

**4CL2_Col-0** 1601 CTAAAGCTCCATCAGGGAAGATATTGAGGAAGGATCTAAGAGCAAGACTAGCAAATGGATTAATGAACTAG

**4CL2_Est-1** 1601 CTAAAGCTCCATCAGGGAAGATATTGAGGAAGGATCTAAGAGCAAGACTAGCAAATGGATTAATGAACTAG

**4CL2_Tsu-1** 1601 CTAAAGCTCCATCAGGGAAGATATTGAGGAAGGATCTAAGAGCAAGACTAGCAAATGGATTAATGAACTAG

**4-coumarate CoA ligase 3 (4CL3, At1g65060)**

10 20 30 40 50 60 70 80 90 100

....|....|....|....|....|....|....|....|....|....|....|....|....|....|....|....|....|....|....|....|

**4CL3_Col-0** 1 ATGATCACTGCAGCTCTACACGAACCTCAGATTCACAAACCAACCGATACATCCGTCGTCTCCGATGATGTGCTTCCTCAT---------TCTCCTCCTA

**4CL3_Est-1** 1 ATGATCACTGCAGCTCTACACGAACCTCAGATTCACAAACCATCCGATACATCCGTCGTCTCCGGGGATGTGCTTCCTCCTCCTCCTCCTTCTCCTCCTA

**4Cl3_Tsu-1** 1 ATGATCACTGCAGCTCTACACGAACCTCAGATTCACAAACCAACCGATACATCCGTCGTCTCCGATGATGTGCTTCCTCAT---------TCTCCTCCTA

110 120 130 140 150 160 170 180 190 200

....|....|....|....|....|....|....|....|....|....|....|....|....|....|....|....|....|....|....|....|

**4CL3_Col-0** 92 CGCCTCGCATTTTCCGGTCAAAGCTTCCGGACATTGACATCCCTAACCACCTCCCTCTCCACACTTACTGCTTCGAAAAACTCTCATCTGTTTCCGACAA

**4CL3_Est-1** 101 CGCCTCGCATTTTCCGGTCAAAGCTTCCGGACATTAACATCCCTAACCACCTCCCTCTCCACACTTACTGCTTTGAAAAACTCTCATCTGTTTCCGACAA

**4Cl3_Tsu-1** 92 CGCCTCGCATTTTCCGGTCAAAGCTTCCGGACATTGACATCCCTAACCACCTCCCTCTCCACACTTACTGCTTCGAAAAACTCTCATCTGTTTCCGACAA

210 220 230 240 250 260 270 280 290 300

....|....|....|....|....|....|....|....|....|....|....|....|....|....|....|....|....|....|....|....|

**4CL3_Col-0** 192 GCCTTGTCTCATCGTTGGCTCCACCGGGAAAAGCTACACCTACGGCGAAACACACCTAATCTGTCGGAGAGTCGCTTCCGGGTTATACAAGTTAGGTATC

**4CL3_Est-1** 201 GCCTTGTCTCATCGTTGGCTCCACCGGGAAAAGCTACACCTACGGCGAAACACACCTAATCTGTCGGAGAGTCGCTTCCGGGTTATGCAAGTTAGGTATA

**4Cl3_Tsu-1** 192 GCCTTGTCTCATCGTTGGCTCCACCGGGAAAAGCTACACCTACGGCGAAACACACCTAATCTGTCGGAGAGTCGCTTCCGGGTTATACAAGTTAGGTATC

310 320 330 340 350 360 370 380 390 400

....|....|....|....|....|....|....|....|....|....|....|....|....|....|....|....|....|....|....|....|

**4CL3_Col-0** 292 AGAAAAGGTGACGTCATCATGATCCTTCTCCAAAACTCAGCCGAGTTCGTTTTCTCCTTCATGGGTGCTTCCATGATCGGAGCCGTCTCAACCACCGCAA

**4CL3_Est-1** 301 AGAAAAGGTGACGTCATCATGATCCTTCTCCAAAACTCAGCCGAGTTCGTTTTCTCCTTCATGGGTGCTTCCATGATCGGAGCCGTCTCAACCACCGCAA

**4Cl3_Tsu-1** 292 AGAAAAGGTGACGTCATCATGATCCTTCTCCAAAACTCAGCCGAGTTCGTTTTCTCCTTCATGGGTGCTTCCATGATCGGAGCCGTCTCAACCACCGCAA

410 420 430 440 450 460 470 480 490 500

....|....|....|....|....|....|....|....|....|....|....|....|....|....|....|....|....|....|....|....|

**4CL3_Col-0** 392 ACCCTTTCTACACTTCTCAAGAGCTTTATAAACAGCTTAAATCTTCCGGAGCCAAACTCATAATCACTCACTCTCAATACGTCGATAAACTTAAAAACCT

**4CL3_Est-1** 401 ACCCTTTCTACACTTCTCAAGAGCTTTATAAACAGCTTAAATCTTCCGGAGCCAAACTCATAATCACTCACTCTCAATACGTCGATAAACTAAAAAACCT

**4Cl3_Tsu-1** 392 ACCCTTTCTACACTTCTCAAGAGCTTTATAAACAGCTTAAATCTTCCGGAGCCAAACTCATAATCACTCACTCTCAATACGTCGATAAACTTAAAAACCT

510 520 530 540 550 560 570 580 590 600

....|....|....|....|....|....|....|....|....|....|....|....|....|....|....|....|....|....|....|....|

**4CL3_Col-0** 492 CGGAGAAAACCTCACGCTGATCACTACCGATGAACCAACACCGGAGAATTGTCTACCATTCTCGACACTCATCACCGACGACGAAACAAACCCATTTCAA

**4CL3_Est-1** 501 CGGAGAAAACCTCACAGTGATCACCACCGATGAACCAACACCGGAGAATTGTCTACCATTCTCGACACTCATCACCGACGACGAAACAAACCCATTTCAA

**4Cl3_Tsu-1** 492 CGGAGAAAACCTCACGCTGATCACTACCGATGAACCAACACCGGAGAATTGTCTACCATTCTCGACACTCATCACCGACGACGAAACAAACCCATTTCAA

610 620 630 640 650 660 670 680 690 700

....|....|....|....|....|....|....|....|....|....|....|....|....|....|....|....|....|....|....|....|

**4CL3_Col-0** 592 GAAACCGTCGATATCGGCGGTGACGATGCGGCGGCGTTACCATTCTCATCGGGAACAACAGGGTTACCAAAAGGAGTTGTTTTGACACACAAGAGCTTAA

**4CL3_Est-1** 601 GAAACCGTCGACATCGGCGGTGACGATGCGGCGGCGCTCCCATTCTCATCGGGAACAACAGGGTTACCAAAAGGAGTTGTTTTGACACACAAGAGCTTAA

**4Cl3_Tsu-1** 592 GAAACCGTCGATATCGGCGGTGACGATGCGGCGGCGTTACCATTCTCATCGGGAACAACAGGGTTACCAAAAGGAGTTGTTTTGACACACAAGAGCTTAA

710 720 730 740 750 760 770 780 790 800

....|....|....|....|....|....|....|....|....|....|....|....|....|....|....|....|....|....|....|....|

**4CL3_Col-0** 692 TCACAAGCGTTGCACAACAAGTAGATGGAGATAACCCAAATCTTTACCTCAAATCAAACGACGTCATACTCTGCGTTTTACCACTTTTCCATATCTACTC

**4CL3_Est-1** 701 TCACAAGCGTTGCACAACAAGTAGATGGAGATAACCCAAATCTTTACCTCAAATCAAACGACGTCATACTCTGCGTTTTACCACTTTTCCATATCTACTC

**4Cl3_Tsu-1** 692 TCACAAGCGTTGCACAACAAGTAGATGGAGATAACCCAAATCTTTACCTCAAATCAAACGACGTCATACTCTGCGTTTTACCACTTTTCCATATCTACTC

810 820 830 840 850 860 870 880 890 900

....|....|....|....|....|....|....|....|....|....|....|....|....|....|....|....|....|....|....|....|

**4CL3_Col-0** 792 TCTCAATAGCGTCCTCCTCAATTCACTCCGATCCGGCGCCACGGTTCTTTTAATGCATAAGTTTGAGATCGGTGCGTTATTGGATCTTATTCAAAGACAT

**4CL3_Est-1** 801 TCTCAATAGCGTCCTCCTCAATTCACTCCGATCCGGCGCCACGGTTCTTTTAATGCATAAGTTTGAGATCGGTGCGTTATTGGATCTTATTCAAAGACAT

**4Cl3_Tsu-1** 792 TCTCAATAGCGTCCTCCTCAATTCACTCCGATCCGGCGCCACGGTTCTTTTAATGCATAAGTTTGAGATCGGTGCGTTATTGGATCTTATTCAAAGACAT

910 920 930 940 950 960 970 980 990 1000

....|....|....|....|....|....|....|....|....|....|....|....|....|....|....|....|....|....|....|....|

**4CL3_Col-0** 892 AGAGTGACAATAGCGGCGCTTGTACCGCCGCTAGTGATCGCTCTAGCTAAAAACCCGACGGTTAATTCTTATGATCTCTCCTCCGTTAGATTCGTTCTTT

**4CL3_Est-1** 901 AGAGTGACAATAGCGGCGCTTGTACCGCCGCTAGTGATCGCTCTAGCTAAAAACCCGACGGTTAATTCTTATGATCTCTCCTCCGTTAGATTCGTTCTTT

**4Cl3_Tsu-1** 892 AGAGTGACAATAGCGGCGCTTGTACCGCCGCTAGTGATCGCTCTAGCTAAAAACCCGACGGTTAATTCTTATGATCTCTCCTCCGTTAGATTCGTTCTTT

1010 1020 1030 1040 1050 1060 1070 1080 1090 1100

....|....|....|....|....|....|....|....|....|....|....|....|....|....|....|....|....|....|....|....|

**4CL3_Col-0** 992 CCGGTGCAGCTCCTTTAGGTAAAGAACTTCAAGATAGTCTTCGTCGCCGTCTCCCTCAAGCCATCCTTGGCCAGGGATATGGTATGACGGAGGCAGGACC

**4CL3_Est-1** 1001 CCGGTGCAGCTCCTTTAGGTAAAGAACTTCAAGATAGTCTTCGTCGCCGTCTCCCTCAAGCCATCCTTGGCCAGGGATATGGTATGACGGAGGCAGGACC

**4Cl3_Tsu-1** 992 CCGGTGCAGCTCCTTTAGGTAAAGAACTTCAAGATAGTCTTCGTCGCCGTCTCCCTCAAGCCATCCTTGGCCAGGGATATGGTATGACGGAGGCAGGACC

1110 1120 1130 1140 1150 1160 1170 1180 1190 1200

....|....|....|....|....|....|....|....|....|....|....|....|....|....|....|....|....|....|....|....|

**4CL3_Col-0** 1092 AGTGTTGTCAATGAGCCTTGGGTTCGCTAAAGAACCGATACCGACAAAATCAGGTTCATGTGGGACTGTGGTCCGAAACGCAGAGCTTAAAGTGGTTCAC

**4CL3_Est-1** 1101 AGTGTTGTCAATGAGCCTTGGGTTCGCTAAAGAACCGATACCGACAAAATCAGGTTCATGTGGGACTGTGGTCCGAAACGCAGAGCTTAAAGTGGTTCAC

**4Cl3_Tsu-1** 1092 AGTGTTGTCAATGAGCCTTGGGTTCGCTAAAGAACCGATACCGACAAAATCAGGTTCATGTGGGACTGTGGTCCGAAACGCAGAGCTTAAAGTGGTTCAC

1210 1220 1230 1240 1250 1260 1270 1280 1290 1300

....|....|....|....|....|....|....|....|....|....|....|....|....|....|....|....|....|....|....|....|

**4CL3_Col-0** 1192 CTTGAGACACGTCTCTCTCTTGGATACAACCAACCTGGTGAGATTTGTATCCGCGGTCAACAGATCATGAAAGAGTACTTGAACGATCCAGAAGCCACTT

**4CL3_Est-1** 1201 CTTGAGACACGTCTCTCTCTTGGATACAACCAACCTGGTGAGATTTGTATCCGCGGTCAACAGATCATGAAAGAGTACTTGAACGATCCAGAAGCCACTT

**4Cl3_Tsu-1** 1192 CTTGAGACACGTCTCTCTCTTGGATACAACCAACCTGGTGAGATTTGTATCCGCGGTCAACAGATCATGAAAGAGTACTTGAACGATCCAGAAGCCACTT

1310 1320 1330 1340 1350 1360 1370 1380 1390 1400

....|....|....|....|....|....|....|....|....|....|....|....|....|....|....|....|....|....|....|....|

**4CL3_Col-0** 1292 CAGCAACAATAGACGAAGAAGGTTGGCTTCACACAGGAGACATTGGATATGTTGATGAAGATGATGAGATTTTCATTGTTGATCGACTTAAAGAAGTCAT

**4CL3_Est-1** 1301 CAGCAACAATAGACGAAGAAGGTTGGCTTCACACAGGAGACATTGGATATGTTGATGAAGATGATGAGATTTTCATTGTTGATCGACTTAAAGAAGTCAT

**4Cl3_Tsu-1** 1292 CAGCAACAATAGACGAAGAAGGTTGGCTTCACACAGGAGACATTGGATATGTTGATGAAGATGATGAGATTTTCATTGTTGATCGACTTAAAGAAGTCAT

1410 1420 1430 1440 1450 1460 1470 1480 1490 1500

....|....|....|....|....|....|....|....|....|....|....|....|....|....|....|....|....|....|....|....|

**4CL3_Col-0** 1392 CAAGTTCAAAGGCTTTCAGGTCCCTCCAGCTGAGCTAGAGAGTTTGCTAATCAATCACCATTCAATTGCCGATGCAGCTGTTGTTCCGCAAAATGATGAA

**4CL3_Est-1** 1401 CAAGTTCAAAGGCTTTCAGGTCCCTCCAGCTGAGCTAGAGAGTTTGCTAATCAATCACCATTCAATTGCCGATGCAGCTGTTGTTCCGCAAAATGATGAA

**4Cl3_Tsu-1** 1392 CAAGTTCAAAGGCTTTCAGGTCCCTCCAGCTGAGCTAGAGAGTTTGCTAATCAATCACCATTCAATTGCCGATGCAGCTGTTGTTCCGCAAAATGATGAA

1510 1520 1530 1540 1550 1560 1570 1580 1590 1600

....|....|....|....|....|....|....|....|....|....|....|....|....|....|....|....|....|....|....|....|

**4CL3_Col-0** 1492 GTCGCTGGGGAAGTTCCGGTGGCTTTCGTGGTGCGATCAAATGGAAATGATATCACTGAAGAAGATGTTAAAGAATATGTTGCCAAACAGGTGGTGTTCT

**4CL3_Est-1** 1501 GTCGCTGGGGAAGTTCCGGTGGCTTTCGTGGTGCGATCAAATGGAAATGATATCACTGAAGAAGATGTTAAAGAATATGTTGCCAAACAGGTGGTGTTCT

**4Cl3_Tsu-1** 1492 GTCGCTGGGGAAGTTCCGGTGGCTTTCGTGGTGCGATCAAATGGAAATGATATCACTGAAGAAGATGTTAAAGAATATGTTGCCAAACAGGTGGTGTTCT

1610 1620 1630 1640 1650 1660 1670 1680 1690

....|....|....|....|....|....|....|....|....|....|....|....|....|....|....|....|....|....|....|

**4CL3_Col-0** 1592 ATAAGAGATTGCACAAGGTCTTCTTTGTTGCTTCCATTCCTAAATCTCCTTCGGGAAAAATACTAAGAAAGGACCTCAAAGCTAAGCTTTGTTGA

**4CL3_Est-1** 1601 ATAAGAGATTGCACAAGGTCTTCTTTGTTGCTTCCATTCCTAAATCTCCTTCGGGAAAAATACTAAGAAAGGACCTCAAAGCTAAGCTTTGTTGA

**4Cl3_Tsu-1** 1592 ATAAGAGATTGCACAAGGTCTTCTTTGTTGCTTCCATTCCTAAATCTCCTTCGGGAAAAATACTAAGAAAGGACCTCAAAGCTAAGCTTTGTTGA

**Feruloyl-CoA 6-Hydroxylase 1 (F6H1, At3g13610)**

10 20 30 40 50 60 70 80 90 100

....|....|....|....|....|....|....|....|....|....|....|....|....|....|....|....|....|....|....|....|

**F6H1_Col-0** 1 ATGGCTCCAACACTCTTGACAACCCAATTCTCAAATCCAGCTGAAGTAACCGACTTTGTAGTCTACAAAGGAAATGGTGTTAAGGGTTTATCAGAAACAG

**F6H1_Est-1** 1 ATGGCTCCAACACTCTTGACAACCCAATTCTCAAATCCAGCTGAAGTAACCGACTTTGTAGTCTACAAAGGAAATGGTGTTAAGGGTTTATCAGAAACAG

**F6H1_Tsu-1** 1 ATGGCTCCAACACTCTTGACAACCCAATTCTCAAATCCAGCTGAAGTAACCGACTTTGTAGTCTACAAAGGGAATGGTGTTAAGGGTTTATCAGAAACAG

110 120 130 140 150 160 170 180 190 200

....|....|....|....|....|....|....|....|....|....|....|....|....|....|....|....|....|....|....|....|

**F6H1_Col-0** 101 GAATCAAAGCTCTTCCAGAACAATACATTCAGCCACTTGAAGAACGACTCATCAACAAATTCGTCAACGAAACAGATGAAGCCATTCCAGTTATCGATAT

**F6H1_Est-1** 101 GAATCAAAGCTCTTCCAGAACAATACATTCAGCCACTTGAAGAACGACTCATCAACAAATTCGTCAACGAAACAGATGAAGCCATTCCAGTTATCGATAT

**F6H1_Tsu-1** 101 GAATCAAAGCTCTTCCAGAACAATACATTCAGCCACTTGAAGAACGACTCATCAACAAATTCGTCAACGAAACAGATGAAGCCATTCCAGTTATCGATAT

210 220 230 240 250 260 270 280 290 300

....|....|....|....|....|....|....|....|....|....|....|....|....|....|....|....|....|....|....|....|

**F6H1_Col-0** 201 GTCGAACCCTGATGAGGACAGAGTCGCTGAAGCTGTTTGTGATGCTGCTGAGAAATGGGGGTTCTTTCAAGTGATCAATCATGGAGTTCCTTTGGAAGTT

**F6H1_Est-1** 201 GTCGAACCCTGATGAGGACAGAGTCGCTGAAGCTGTTTGTGATGCTGCTGAGAAATGGGGGTTCTTTCAAGTGATCAATCATGGAGTTCCTTTGGAAGTT

**F6H1_Tsu-1** 201 GTCGAACCTTGATGAGGACAGAGTCGCTGAAGCTGTTTGTGATGCTGCTGAGAAATGGGGGTTCTTTCAAGTGATCAATCATGGAGTTCCTTTGGAAGTT

310 320 330 340 350 360 370 380 390 400

....|....|....|....|....|....|....|....|....|....|....|....|....|....|....|....|....|....|....|....|

**F6H1_Col-0** 301 CTTGATGACGTCAAGGCTGCGACTCACAAGTTCTTCAATCTCCCTGTTGAAGAGAAGCGCAAGTTCACTAAAGAGAATTCGCTGTCGACGACTGTTAGGT

**F6H1_Est-1** 301 CTTGATGACGTCAAGGCTGCGACTCACAAGTTCTTCAATCTCCCTGTTGAAGAGAAGCGCAAGTTCACTAAAGAGAATTCGCTGTCGACGACTGTTAGGT

**F6H1_Tsu-1** 301 CTTGATGACGTCAAGGCTGCGACTCACAAGTTCTTCAATCTCCCTGTTGAAGAGAAGCGCAAGTTCACTAAAGAGAATTCGCTGTCGACGACTGTTAGGT

410 420 430 440 450 460 470 480 490 500

....|....|....|....|....|....|....|....|....|....|....|....|....|....|....|....|....|....|....|....|

**F6H1_Col-0** 401 TTGGGACGAGTTTTAGTCCTCTTGCAGAGCAAGCGCTTGAGTGGAAAGATTATCTCAGCCTCTTCTTTGTCTCTGAAGCTGAAGCTGAACAGTTCTGGCC

**F6H1_Est-1** 401 TTGGGACGAGTTTTAGTCCTCTTGCAGAGCAAGCGCTTGAGTGGAAAGATTATCTCAGCCTCTTCTTTGTCTCTGAAGCTGAAGCTGAACAGTTCTGGCC

**F6H1_Tsu-1** 401 TTGGGACGAGTTTTAGTCCTCTTGCAGAGCAAGCGCTTGAGTGGAAAGATTATCTCAGCCTCTTCTTTGTCTCTGAAGCTGAAGCTGAACAGTTCTGGCC

510 520 530 540 550 560 570 580 590 600

....|....|....|....|....|....|....|....|....|....|....|....|....|....|....|....|....|....|....|....|

**F6H1_Col-0** 501 TGATATCTGCAGGAATGAAACGTTAGAGTACATTAACAAGTCAAAGAAGATGGTGAGGAGGCTTCTAGAGTATTTGGGAAAGAATCTCAATGTTAAAGAG

**F6H1_Est-1** 501 TGATATCTGCAGGAATGAAACGTTAGAGTACATTAACAAGTCAAAGAAGATGGTGAGGAGGCTTCTAGAGTATTTGGGAAAGAATCTCAATGTTAAAGAG

**F6H1_Tsu-1** 501 TGATATCTGCAGGAATGAAACGTTAGAGTACATTAACAAGTCAAAGAAGATGGTGAGGAGGCTTCTAGAGTATTTGGGAAAGAATCTCAATGTTAAAGAG

610 620 630 640 650 660 670 680 690 700

....|....|....|....|....|....|....|....|....|....|....|....|....|....|....|....|....|....|....|....|

**F6H1_Col-0** 601 CTTGACGAGACGAAAGAATCACTCTTTATGGGCTCGATTCGAGTCAACCTTAACTACTACCCCATCTGCCCTAATCCGGACCTAACAGTTGGTGTTGGTC

**F6H1_Est-1** 601 CTTGACGAGACGAAAGAATCACTCTTTATGGGCTCGATTCGAGTCAACCTTAACTACTACCCCATCTGCCCTAATCCGGACCTAACAGTTGGTGTTGGTC

**F6H1_Tsu-1** 601 CTTGACGAGACGAAAGAATCACTCTTTATGGGCTCGATTCGAGTCAACCTTAACTACTACCCCATCTGCCCTAATCCGGACCTAACAGTTGGTGTTGGTC

710 720 730 740 750 760 770 780 790 800

....|....|....|....|....|....|....|....|....|....|....|....|....|....|....|....|....|....|....|....|

**F6H1_Col-0** 701 GCCACTCAGACGTCTCTTCTCTCACCATTCTCTTACAAGACCAGATCGGTGGTCTACACGTGCGTTCTCTGGCTTCAGGGAACTGGGTTCACGTGCCTCC

**F6H1_Est-1** 701 GCCACTCAGACGTCTCTTCTCTCACCATTCTCTTACAAGACCAGATCGGTGGTCTACACGTGCGTTCTCTGGCTTCAGGGAACTGGGTTCACGTGCCTCC

**F6H1_Tsu-1** 701 GCCACTCAGACGTCTCTTCTCTCACCATTCTCTTACAAGACCAGATCGGTGGTCTACACGTGCGTTCTCTGGCTTCAGGGAACTGGGTTCACGTGCCTCC

810 820 830 840 850 860 870 880 890 900

....|....|....|....|....|....|....|....|....|....|....|....|....|....|....|....|....|....|....|....|

**F6H1_Col-0** 801 GGTTGCTGGATCTTTTGTGATCAACATCGGAGATGCGATGCAGATCATGAGCAATGGTCTGTACAAGAGCGTGGAGCATCGTGTCTTAGCCAATGGTTAC

**F6H1_Est-1** 801 GGTTGCTGGATCTTTTGTGATCAACATCGGAGATGCGATGCAGATCATGAGCAATGGTCTGTACAAGAGCGTGGAGCATCGTGTCTTAGCCAATGGTTAC

**F6H1_Tsu-1** 801 GGTTGCTGGATCTTTTGTGATCAACATCGGAGATGCGATGCAGATCATGAGCAATGGTCTGTACAAGAGCGTGGAGCATCGTGTCTTAGCCAATGGTTAC

910 920 930 940 950 960 970 980 990 1000

....|....|....|....|....|....|....|....|....|....|....|....|....|....|....|....|....|....|....|....|

**F6H1_Col-0** 901 AATAATAGAATCTCTGTTCCTATCTTTGTGAACCCAAAACCAGAGTCAGTTATTGGTCCTCTACCTGAGGTGATTGCAAACGGAGAGGAACCGATTTACA

**F6H1_Est-1** 901 AATAATAGAATCTCTGTTCCTATCTTTGTGAACCCAAAACCAGAGTCAGTTATTGGTCCTCTACCTGAGGTGATTGCAAACGGAGAGGAACCGATTTACA

**F6H1_Tsu-1** 901 AATAATAGAATCTCTGTTCCTATCTTTGTGAACCCAAAACCAGAGTCAGTTATTGGTCCTCTACCTGAGGTGATTGCAAACGGAGAGGAACCGATTTACA

1010 1020 1030 1040 1050 1060 1070 1080

....|....|....|....|....|....|....|....|....|....|....|....|....|....|....|....|....|.

**F6H1_Col-0** 1001 GAGACGTCCTGTACTCTGATTACGTCAAGTATTTCTTCAGGAAGGCACACGATGGAAAGAAAACCGTCGATTACGCCAAGATCTGA

**F6H1_Est-1** 1001 GAGACGTCCTGTACTCTGATTACGTCAAGTATTTCTTCAGGAAGGCACACGATGGAAAGAAAACCGTCGATTACGCCAAGATCTGA

**F6H1_Tsu-1** 1001 GAGACGTCCTGTACTCTGATTACGTCAAGTATTTCTTCAGGAAGGCACACGATGGAAAGAAAACCGTCGATTACGCCAAGATCTGA

**Feruloyl-CoA 6-Hydroxylase 2 (F6H2, At1g55290)**

10 20 30 40 50 60 70 80 90 100

....|....|....|....|....|....|....|....|....|....|....|....|....|....|....|....|....|....|....|....|

**F6H2_Col-0** 1 ATGAATCAAACACTCGCTGCCCAATTCTTAACCCGAGACCAAGTCACCAACTTTGTTGTACACGAAGGTAACGGTGTTAAAGGCTTGTCTGAGACCGGAA

**F6H2_Est-1** 1 ATGAATCAAACACTCGCTGCCCAATTCTTAACCCCTGACCAAGTCACCAACTTTGTTGTACACGAAGGTAACGGTGTTAAAGGCTTGTCTGAGACCGGAA

**F6H2_Tsu-1** 1 ATGAATCAAACACTCGCTGCCCAATTCTTAACCCGAGACCAAGTCACCAACTTCGTTGTCCATGAAGGTAACGGCGTTAAAGGCTTGTCTGAAACCGGAA

110 120 130 140 150 160 170 180 190 200

....|....|....|....|....|....|....|....|....|....|....|....|....|....|....|....|....|....|....|....|

**F6H2_Col-0** 101 TCAAAGTTCTTCCTGACCAATACATTCAGCCATTCGAAGAGAGACTGATCAACTTCCACGTAAAAGAGGATTCAGACGAATACATTCAGCCATTCGAAGA

**F6H2_Est-1** 101 TCAAAGTTCTTCCTGACCAATACATTCAGCCATTCGAAGAGAGACTGATCAACTTCCACGTAAAAGAGGATTCAGACGAATACATTCAGCCATTCGAAGA

**F6H2_Tsu-1** 101 TCAAAGTTCTTCCTGACCAATACATTCAGCCATTCGAAGAGAGACTGATCAACTTCCACGTAAAAGAGGATTCAGACGAATACATTCAGCCATTCGAAGA

210 220 230 240 250 260 270 280 290 300

....|....|....|....|....|....|....|....|....|....|....|....|....|....|....|....|....|....|....|....|

**F6H2_Col-0** 201 GAGACTGATCAACTTCCACGTAAAAGAGGATTCAGACGAATCCATACCCGTGATCGACATATCAAATTTAGACGAGAAGAGTGTCTCCAAGGCCGTATGT

**F6H2_Est-1** 201 GAGACTGATCAACTTCCACGTAAAAGAGGATTCAGACGAATCCATACCCGTGATCGACATATCAAATTTAGACGAGAAGAGTGTCTCCAAGGCCGTATGT

**F6H2_Tsu-1** 201 GAGACTGATCAACTTCCACGTAAAAGAGGATTCAGACGAATCCATACCCGTGATCGACATGTCGAATTTAGACGAGAAGAGTGTCTCCAAGGCCGTATGT

310 320 330 340 350 360 370 380 390 400

....|....|....|....|....|....|....|....|....|....|....|....|....|....|....|....|....|....|....|....|

**F6H2_Col-0** 301 GATGCTGCAGAAGAATGGGGTTTCTTTCAGGTGATCAACCATGGCGTGTCAATGGAAGTTCTTGAGAATATGAAAACAGCTACTCACAGATTCTTCGGTT

**F6H2_Est-1** 301 GATGCTGCAGAAGAATGGGGTTTCTTTCAGGTGATCAACCATGGCGTGTCAATGGAAGTTCTTGAGAATATGAAAACAGCTACTCACAGATTCTTCGGTT

**F6H2_Tsu-1** 301 GATGCTGCAGAAGAATGGGGTTTCTTTCAGGTGATCAACCATGGCGTATCAATGGAAGTTCTTGAGAATATGAAAACAGCTACTCACAGATTCTTCGGTT

410 420 430 440 450 460 470 480 490 500

....|....|....|....|....|....|....|....|....|....|....|....|....|....|....|....|....|....|....|....|

**F6H2_Col-0** 401 TACCGGTAGAAGAGAAAAGAAAGTTCTCAAGAGAGAAGTCTTTGTCAACGAATGTGAGATTCGGGACGAGTTTTAGTCCTCATGCTGAGAAAGCTCTCGA

**F6H2_Est-1** 401 TACCGGTAGAAGAGAAAAGAAAGTTCTCAAGAGAGAAGTCTTTGTCAACGAATGTGAGATTCGGGACGAGTTTTAGTCCTCATGCTGAGAAAGCTCTCGA

**F6H2_Tsu-1** 401 TACCGGTAGAAGAGAAAAGAAAGTTCTCAAGAGAGAAGTCTTTGTCAACGAATGTGAGATTCGGGACGAGTTTTAGTCCTCATGCTGAGAAAGCTCTCGA

510 520 530 540 550 560 570 580 590 600

....|....|....|....|....|....|....|....|....|....|....|....|....|....|....|....|....|....|....|....|

**F6H2_Col-0** 501 GTGGAAAGATTATCTGAGCCTCTTCTTTGTCTCTGAAGCTGAAGCATCACAACTCTGGCCTGACTCTTGCAGGAGTGAAACGCTAGAATACATGAACGAG

**F6H2_Est-1** 501 GTGGAAAGATTATCTGAGCCTCTTCTTTGTCTCTGAAGCTGAAGCATCACAACTCTGGCCTGACTCTTGCAGGAGTGAAACGCTAGAATACATGAACGAG

**F6H2_Tsu-1** 501 GTGGAAAGATTATCTGAGCCTCTTCTTTGTCTCTGAAGCTGAAGCATCACAACTCTGGCCTGACTCTTGCAGGAGTGAAACGCTAGAATACATGAACGAG

610 620 630 640 650 660 670 680 690 700

....|....|....|....|....|....|....|....|....|....|....|....|....|....|....|....|....|....|....|....|

**F6H2_Col-0** 601 ACAAAACCTCTAGTGAAGAAACTCTTACGGTTTCTAGGCGAGAATCTGAACGTGAAAGAGCTAGACAAGACCAAAGAGTCATTCTTCATGGGTTCAACAC

**F6H2_Est-1** 601 ACAAAACCTCTAGTGAAGAAACTCTTACGGTTTCTAGGCGAGAATCTGAACGTGAAAGAGCTAGACAAGACCAAAGAGTCATTCTTCATGGGTTCAACAC

**F6H2_Tsu-1** 601 ACAAAACCTCTAGTGAAGAAACTCTTACGGTTTCTAGGCGAGAATCTGAACGTGACAGAGCTAGACAAGACCAAAGAGTCACTCTTCATGGGTTCAACAC

710 720 730 740 750 760 770 780 790 800

....|....|....|....|....|....|....|....|....|....|....|....|....|....|....|....|....|....|....|....|

**F6H2_Col-0** 701 GTATCAACCTCAACTATTACCCTATTTGTCCCAATCCAGAACTCACGGTTGGAGTCGGACGTCACTCTGATGTTTCCTCACTCACAATCCTCTTACAAGA

**F6H2_Est-1** 701 GTATCAACCTCAACTATTACCCTATTTGTCCCAATCCAGAACTCACGGTTGGAGTCGGACGTCACTCTGATGTTTCCTCACTCACAATCCTCTTACAAGA

**F6H2_Tsu-1** 701 GTATCAACCTCAACTATTACCCTATTTGTCCCAATCCAGAACTCACGGTTGGAGTCGGACGTCACTCTGATGTTTCCTCACTCACAATCCTCTTACAAGA

810 820 830 840 850 860 870 880 890 900

....|....|....|....|....|....|....|....|....|....|....|....|....|....|....|....|....|....|....|....|

**F6H2_Col-0** 801 CGAGATCGGTGGTCTCCACGTTCGTTCTCTCACCACGGGGAGATGGGTTCACGTGCCTCCAATCTCCGGATCTTTAGTCATTAACATTGGAGACGCTATG

**F6H2_Est-1** 801 CGAGATCGGTGGTCTCCACGTTCGTTCTCTCACCACGGGGAGATGGGTTCACGTGCCTCCAATCTCCGGATCTTTAGTCATTAACATTGGAGACGCTATG

**F6H2_Tsu-1** 801 CGAGATCGGTGGTCTCCACGTTCGTTCTCTCACCACGGGGAGATGGGTTCACGTGCCTCCAATCTCCGGATCTTTAGTCATTAACATTGGAGACGCTATG

910 920 930 940 950 960 970 980 990 1000

....|....|....|....|....|....|....|....|....|....|....|....|....|....|....|....|....|....|....|....|

**F6H2_Col-0** 901 CAAATCATGAGTAATGGTCGTTACAAGAGTGTTGAGCATCGTGTCTTAGCTAACGGTTCTTATAACAGAATCTCTGTTCCTATTTTCGTGAGCCCGAAAC

**F6H2_Est-1** 901 CAAATCATGAGTAATGGTCGTTACAAGAGTGTTGAGCATCGTGTCTTAGCTAACGGTTCTTATAACAGAATCTCTGTTCCTATTTTCGTGAGCCCGAAAC

**F6H2_Tsu-1** 901 CAAATCATGAGTAATGGTCGTTACAAGAGTGTTGAGCATCGTGTCTTAGCTAACAGTTCTTATAACAGAATCTCTGTTCCTATTTTCGTGAACCCGAAAC

1010 1020 1030 1040 1050 1060 1070 1080 1090 1100

....|....|....|....|....|....|....|....|....|....|....|....|....|....|....|....|....|....|....|....|

**F6H2_Col-0** 1001 CAGAGTCTGTGATCGGTCCTCTTCTTGAAGTGATCGAAAATGGAGAGAAACCGGTTTATAAAGATATTCTTTATACCGATTACGTGAAACATTTCTTCAG

**F6H2_Est-1** 1001 CAGAGTCTGTGATCGGTCCTCTTCTTGAAGTGATCGAAAATGGAGAGAAACCGGTTTATAAAGATATTCTTTATACCGATTACGTGAAACATTTCTTCAG

**F6H2_Tsu-1** 1001 CAGAGTCTGTGATCGGTCCTCTTCTTGAAGTGATCGAAAATGGAGAGAAACCGGTTTATAGAGATATACTTTATACCGATTACGTGAAACATTTCTTCAG

1110 1120 1130 1140

....|....|....|....|....|....|....|....|....|.

**F6H2_Col-0** 1101 AAAAGCTCATGATGGGAAGAAAACCATCGATTTTGCCAACATTTGA

**F6H2_Est-1** 1101 AAAAGCTCATGATGGGAAGAAAACCATCGATTTTGCCAACATTTGA

**F6H2_Tsu-1** 1101 AAAAGCCCATGATGGGAAGAAAACCATCGATTTTGCCAACATTTGA

**p-coumaroyl 3-hydroxylase (C3H, At2g40890)**

10 20 30 40 50 60 70 80 90 100

....|....|....|....|....|....|....|....|....|....|....|....|....|....|....|....|....|....|....|....|

**C3H_Col-0** 1 ATGTCGTGGTTTCTAATAGCGGTGGCGACAATCGCCGCCGTCGTATCCTACAAGCTAATCCAACGGCTAAGATACAAGTTCCCACCAGGCCCAAGCCCCA

**C3H_Est-1** 1 ATGTCGTGGTTTCTAATAGCGGTGGCGACAATCGCCGCCGTCGTATCCTACAAGCTAATCCAACGGCTAAGATACAAGTTCCCACCAGGCCCAAGCCCCA

**C3H_Tsu-1** 1 ATGTCGTGGTTTCTAATAGCGGTGGCGACAATCGCCGCCGTCGTATCCTACAAGCTAATCCAACGGCTAAGATACAAGTTCCCACCAGGCCCAAGCCCCA

110 120 130 140 150 160 170 180 190 200

....|....|....|....|....|....|....|....|....|....|....|....|....|....|....|....|....|....|....|....|

**C3H_Col-0** 101 AGCCGATCGTCGGTAACCTCTACGACATAAAACCGGTCCGGTTCAGATGTTACTACGAGTGGGCTCAATCTTATGGACCAATCATATCGGTCTGGATCGG

**C3H_Est-1** 101 AGCCGATCGTCGGTAACCTCTACGACATAAAACCGGTCCGGTTCAGATGTTACTACGAGTGGGCTCAATCTTATGGACCAATCATATCGGTCTGGATCGG

**C3H_Tsu-1** 101 AGCCGATCGTCGGTAACCTCTACGACATAAAACCGGTCCGGTTCAGATGTTACTACGAGTGGGCTCAATCTTATGGACCAATCATATCGGTCTGGATCGG

210 220 230 240 250 260 270 280 290 300

....|....|....|....|....|....|....|....|....|....|....|....|....|....|....|....|....|....|....|....|

**C3H_Col-0** 201 TTCAATTCTAAACGTGGTCGTATCTAGCGCCGAGCTAGCAAAAGAAGTTCTGAAAGAACACGACCAGAAACTCGCCGACCGGCACCGGAACAGATCGACG

**C3H_Est-1** 201 TTCAATTCTAAACGTGGTCGTATCTAGCGCCGAGCTAGCAAAAGAAGTTCTGAAAGAACACGACCAGAAACTCGCCGACCGGCACCGGAACAGATCGACG

**C3H_Tsu-1** 201 TTCAATTCTAAACGTGGTCGTATCTAGCGCCGAGCTAGCAAAAGAAGTTCTGAAAGAACACGACCAGAAACTCGCCGACCGGCACCGGAACAGATCGACG

310 320 330 340 350 360 370 380 390 400

....|....|....|....|....|....|....|....|....|....|....|....|....|....|....|....|....|....|....|....|

**C3H_Col-0** 301 GAAGCATTTAGCCGCAACGGTCAGGATCTTATATGGGCCGATTATGGGCCTCATTACGTGAAGGTGAGAAAAGTTTGCACGCTTGAGCTCTTCACACCGA

**C3H_Est-1** 301 GAAGCATTTAGCCGCAACGGTCAGGATCTTATATGGGCCGATTATGGGCCTCATTACGTGAAGGTGAGAAAAGTTTGCACGCTTGAGCTCTTCACACCGA

**C3H_Tsu-1** 301 GAAGCATTTAGCCGCAACGGTCAGGATCTTATATGGGCCGATTATGGGCCTCATTACGTGAAGGTGAGAAAAGTTTGCACGCTTGAGCTCTTCACACCGA

410 420 430 440 450 460 470 480 490 500

....|....|....|....|....|....|....|....|....|....|....|....|....|....|....|....|....|....|....|....|

**C3H_Col-0** 401 AACGACTCGAGTCTCTCAGACCTATCCGTGAAGATGAAGTCACCGCCATGGTTGAATCCGTCTTCAGAGACTGTAACCTTCCTGAAAACAGAGCAAAAGG

**C3H_Est-1** 401 AACGACTCGAGTCTCTCAGACCTATCCGTGAAGATGAAGTCACCGCCATGGTTGAATCCGTCTTCAGAGACTGTAACCTTCCTGAAAACAGAGCAAAAGG

**C3H_Tsu-1** 401 AACGACTCGAGTCTCTCAGACCTATCCGTGAAGATGAAGTCACCGCCATGGTTGAATCCGTCTTCAGAGACTGTAACCTTCCTGAAAACAGAGCAAAAGG

510 520 530 540 550 560 570 580 590 600

....|....|....|....|....|....|....|....|....|....|....|....|....|....|....|....|....|....|....|....|

**C3H_Col-0** 501 TTTACAACTGAGGAAGTACTTAGGAGCGGTTGCGTTCAACAACATAACGCGGCTAGCCTTTGGGAAGCGTTTTATGAACGCTGAAGGTGTTGTGGACGAG

**C3H_Est-1** 501 TTTACAACTGAGGAAGTACTTAGGAGCGGTTGCGTTCAACAACATAACGCGGCTAGCCTTTGGGAAGCGTTTTATGAACGCTGAAGGTGTTGTGGACGAG

**C3H_Tsu-1** 501 TTTACAACTGAGGAAGTACTTAGGAGCGGTTGCGTTCAACAACATAACGCGGCTAGCCTTTGGGAAGCGTTTTATGAACGCTGAAGGTGTTGTGGACGAG

610 620 630 640 650 660 670 680 690 700

....|....|....|....|....|....|....|....|....|....|....|....|....|....|....|....|....|....|....|....|

**C3H_Col-0** 601 CAAGGGCTTGAGTTCAAGGCCATAGTATCCAACGGTCTGAAGCTAGGTGCTTCACTGTCAATAGCTGAACACATCCCGTGGCTCAGGTGGATGTTTCCGG

**C3H_Est-1** 601 CAAGGGCTTGAGTTCAAGGCCATAGTATCCAACGGTCTGAAGCTAGGTGCTTCACTGTCAATAGCTGAACACATCCCGTGGCTCAGGTGGATGTTTCCGG

**C3H_Tsu-1** 601 CAAGGGCTTGAGTTCAAGGCCATAGTATCCAACGGTCTGAAGCTAGGTGCTTCACTGTCAATAGCTGAACACATCCCGTGGCTCAGGTGGATGTTTCCGG

710 720 730 740 750 760 770 780 790 800

....|....|....|....|....|....|....|....|....|....|....|....|....|....|....|....|....|....|....|....|

**C3H_Col-0** 701 CTGATGAGAAGGCGTTTGCTGAGCACGGGGCTCGTCGTGACCGCCTCACTCGAGCTATCATGGAGGAGCATACTTTGGCCCGTCAAAAGTCTAGTGGAGC

**C3H_Est-1** 701 CTGATGAGAAGGCGTTTGCTGAGCACGGGGCTCGTCGTGACCGCCTCACTCGAGCTATCATGGAGGAGCATACTTTGGCCCGTCAAAAGTCTAGTGGAGC

**C3H_Tsu-1** 701 CTGATGAGAAGGCGTTTGCTGAGCACGGGGCTCGTCGTGACCGCCTCACTCGAGCTATCATGGAGGAGCATACTTTGGCCCGTCAAAAGTCTAGTGGAGC

810 820 830 840 850 860 870 880 890 900

....|....|....|....|....|....|....|....|....|....|....|....|....|....|....|....|....|....|....|....|

**C3H_Col-0** 801 GAAACAGCATTTCGTTGATGCGTTGCTAACGTTGAAGGATCAGTATGATCTTAGTGAGGATACTATCATTGGTCTTCTATGGGATATGATCACGGCAGGG

**C3H_Est-1** 801 GAAACAGCATTTCGTTGATGCGTTGCTAACGTTGAAGGATCAGTATGATCTTAGTGAGGATACTATCATTGGTCTTCTATGGGATATGATCACGGCAGGG

**C3H_Tsu-1** 801 GAAACAGCATTTCGTTGATGCGTTGCTAACGTTGAAGGATCAGTATGATCTTAGTGAGGATACTATCATTGGTCTTCTATGGGATATGATCACGGCAGGG

910 920 930 940 950 960 970 980 990 1000

....|....|....|....|....|....|....|....|....|....|....|....|....|....|....|....|....|....|....|....|

**C3H_Col-0** 901 ATGGACACGACAGCGATAACAGCGGAATGGGCGATGGCGGAAATGATCAAGAATCCAAGAGTGCAACAAAAAGTGCAAGAAGAGTTCGACAGAGTGGTTG

**C3H_Est-1** 901 ATGGACACGACAGCGATAACAGCGGAATGGGCGATGGCGGAAATGATCAAGAATCCAAGAGTGCAACAAAAAGTGCAAGAAGAGTTCGACAGAGTGGTTG

**C3H_Tsu-1** 901 ATGGACACGACAGCGATAACAGCGGAATGGGCGATGGCGGAAATGATCAAGAATCCAAGAGTGCAACAAAAAGTGCAAGAAGAGTTCGACAGAGTGGTTG

1010 1020 1030 1040 1050 1060 1070 1080 1090 1100

....|....|....|....|....|....|....|....|....|....|....|....|....|....|....|....|....|....|....|....|

**C3H_Col-0** 1001 GACTTGACCGGATCTTAACCGAGGCAGATTTCTCCCGCTTACCTTACTTGCAATGCGTGGTGAAAGAGTCATTCAGGCTGCATCCTCCAACGCCTCTAAT

**C3H_Est-1** 1001 GACTTGACCGGATCTTAACCGAGGCAGATTTCTCCCGCTTACCTTACTTGCAATGCGTGGTGAAAGAGTCATTCAGGCTGCATCCTCCAACGCCTCTAAT

**C3H_Tsu-1** 1001 GACTTGACCGGATCTTAACCGAGGCAGATTTCTCCCGCTTACCTTACTTGCAATGCGTGGTGAAAGAGTCATTCAGGCTGCATCCTCCAACGCCTCTAAT

1110 1120 1130 1140 1150 1160 1170 1180 1190 1200

....|....|....|....|....|....|....|....|....|....|....|....|....|....|....|....|....|....|....|....|

**C3H_Col-0** 1101 GCTACCTCACCGAAGCAACGCAGATGTCAAGATCGGAGGCTATGATATTCCCAAAGGATCAAACGTTCATGTGAATGTGTGGGCTGTGGCTAGAGACCCG

**C3H_Est-1** 1101 GCTACCTCACCGAAGCAACGCAGATGTCAAGATCGGAGGCTATGATATTCCCAAAGGATCAAACGTTCATGTGAATGTGTGGGCTGTGGCTAGAGACCCG

**C3H_Tsu-1** 1101 GCTACCTCACCGAAGCAACGCAGATGTCAAGATCGGAGGCTATGATATTCCCAAAGGATCAAACGTTCATGTGAATGTGTGGGCTGTGGCTAGAGACCCG

1210 1220 1230 1240 1250 1260 1270 1280 1290 1300

....|....|....|....|....|....|....|....|....|....|....|....|....|....|....|....|....|....|....|....|

**C3H_Col-0** 1201 GCTGTATGGAAAAATCCATTTGAGTTTAGACCAGAGAGATTCTTGGAAGAAGATGTTGACATGAAGGGTCATGATTTTAGGCTGCTTCCGTTTGGAGCTG

**C3H_Est-1** 1201 GCTGTATGGAAAAATCCATTTGAGTTTAGACCAGAGAGATTCTTGGAAGAAGATGTTGACATGAAGGGTCATGATTTTAGGCTGCTTCCGTTTGGAGCTG

**C3H_Tsu-1** 1201 GCTGTATGGAAAAATCCATTTGAGTTTAGACCAGAGAGATTCTTGGAAGAAGATGTTGACATGAAGGGTCATGATTTTAGGCTGCTTCCGTTTGGAGCTG

1310 1320 1330 1340 1350 1360 1370 1380 1390 1400

....|....|....|....|....|....|....|....|....|....|....|....|....|....|....|....|....|....|....|....|

**C3H_Col-0** 1301 GAAGACGGGTTTGTCCCGGTGCACAACTTGGTATCAATTTGGTAACTTCGATGATGAGTCATTTGCTTCACCATTTTGTTTGGACACCTCCTCAAGGGAC

**C3H_Est-1** 1301 GAAGACGGGTTTGTCCCGGTGCACAACTTGGTATCAATTTGGTAACTTCGATGATGAGTCATTTGCTTCACCATTTTGTTTGGACACCTCCTCAAGGGAC

**C3H_Tsu-1** 1301 GAAGACGGGTTTGTCCCGGTGCACAACTTGGTATCAATTTGGTAACTTCGATGATGAGTCATTTGCTTCACCATTTTGTTTGGACACCTCCTCAAGGGAC

1410 1420 1430 1440 1450 1460 1470 1480 1490 1500

....|....|....|....|....|....|....|....|....|....|....|....|....|....|....|....|....|....|....|....|

**C3H_Col-0** 1401 TAAACCGGAGGAGATTGACATGTCTGAAAACCCTGGACTCGTTACTTACATGCGTACCCCTGTGCAAGCGGTTGCAACGCCTCGGTTGCCTTCGGATCTG

**C3H_Est-1** 1401 TAAACCGGAGGAGATTGACATGTCTGAAAACCCTGGACTCGTTACTTACATGCGTACCCCTGTGCAAGCAGTTGCAACGCCTCGGTTGCCTTCGGATCTG

**C3H_Tsu-1** 1401 TAAACCGGAGGAGATTGACATGTCTGAAAACCCTGGACTCGTTACTTACATGCGTACCCCTGTGCAAGCGGTTGCAACGCCTCGGTTGCCTTCGGATCTG

1510 1520

....|....|....|....|....|..

**C3H_Col-0** 1501 TACAAACGCGTGCCTTACGATATGTAA

**C3H_Est-1** 1501 TACAAACGCGTGCCTTACGATATGTAA

**C3H_Tsu-1** 1501 TACAAACGCGTGCCTTACGATATGTAA

**shikimate O-hydroxycinnamoyltransferase (HCT, At5g48930)**

10 20 30 40 50 60 70 80 90 100

....|....|....|....|....|....|....|....|....|....|....|....|....|....|....|....|....|....|....|....|

**HCT_Col-0** 1 ATGAAAATTAACATCAGAGATTCCACCATGGTCCGGCCTGCCACCGAGACACCAATCACTAATCTTTGGAACTCCAACGTCGACCTTGTCATCCCCAGAT

**HCT_Est-1** 1 ATGAAAATTAACATCAGAGATTCCACCATGGTCCGGCCTGCCACCGAGACACCAATCACTAATCTTTGGAACTCCAACGTCGACCTTGTCATCCCCAGAT

**HCT_Tsu-1** 1 ATGAAAATTAACATCAGAGATTCCACCATGGTCCGGCCTGCCACCGAGACACCAATCACTAATCTTTGGAACTCCAACGTCGACCTTGTCATCCCCAGAT

110 120 130 140 150 160 170 180 190 200

....|....|....|....|....|....|....|....|....|....|....|....|....|....|....|....|....|....|....|....|

**HCT_Col-0** 101 TCCATACCCCTAGTGTCTACTTCTACAGACCCACCGGCGCTTCCAATTTCTTTGACCCTCAGGTCATGAAGGAAGCTCTTTCCAAAGCCCTTGTCCCTTT

**HCT_Est-1** 101 TCCATACCCCTAGTGTCTACTTCTACAGACCCACCGGCGCTTCCAATTTCTTTGACCCTCAGGTCATGAAGGAAGCTCTTTCCAAAGCCCTTGTCCCTTT

**HCT_Tsu-1** 101 TCCATACCCCTAGTGTCTACTTCTACAGACCCACCGGCGCTTCCAATTTCTTTGACCCTCAGGTCATGAAGGAAGCTCTTTCCAAAGCCCTTGTCCCTTT

210 220 230 240 250 260 270 280 290 300

....|....|....|....|....|....|....|....|....|....|....|....|....|....|....|....|....|....|....|....|

**HCT_Col-0** 201 TTACCCTATGGCTGGTCGCTTGAAGAGAGACGATGATGGTCGTATTGAGATCGATTGTAACGGTGCTGGTGTTCTCTTCGTTGTGGCTGATACTCCTTCT

**HCT_Est-1** 201 TTACCCTATGGCTGGTCGCTTGAAGAGAGACGATGATGGTCGTATTGAGATCGATTGTAACGGTGCTGGTGTTCTCTTCGTTGTGGCTGATACTCCTTCT

**HCT_Tsu-1** 201 TTACCCTATGGCTGGTCGCTTGAAGAGAGACGATGATGGTCGTATTGAGATCGATTGTAACGGTGCCGGTGTTCTCTTCGTTGTTGCTGATACTCCTTCT

310 320 330 340 350 360 370 380 390 400

....|....|....|....|....|....|....|....|....|....|....|....|....|....|....|....|....|....|....|....|

**HCT_Col-0** 301 GTTATCGATGATTTTGGTGATTTTGCTCCTACCCTTAATCTCCGTCAGCTTATTCCCGAAGTTGATCACTCCGCTGGCATTCACTCTTTCCCGCTTCTCG

**HCT_Est-1** 301 GTTATCGATGATTTTGGTGATTTTGCTCCTACCCTTAATCTCCGTCAGCTTATTCCCGAAGTTGATCACTCCACCGGCATTCACTCTTTCCCGCTTCTCG

**HCT_Tsu-1** 301 GTTATCGATGATTTTGGTGATTTTGCTCCTACCCTTAATCTCCGTCAGCTTATTCCCGAAGTTGATCACTCCACCGGCATTCACTCTTTCCCGCTTCTCG

410 420 430 440 450 460 470 480 490 500

....|....|....|....|....|....|....|....|....|....|....|....|....|....|....|....|....|....|....|....|

**HCT_Col-0** 401 TTTTGCAGGTGACTTTCTTTAAATGTGGGGGAGCTTCACTTGGGGTTGGGATGCAACATCACGCGGCAGATGGTTTCTCTGGTCTTCATTTTATCAACAC

**HCT_Est-1** 401 TTTTGCAGGTGACTTTCTTTAAATGTGGGGGAGCTTCACTTGGGGTTGGGATGCAACATCACGCGGCAGATGGTTTCTCTGGTCTTCATTTTATCAACAC

**HCT_Tsu-1** 401 TTTTGCAGGTGACTTTCTTTAAATGTGGGGGAGCTTCACTTGGGGTTGGGATGCAACATCACGCGGCAGATGGTTTCTCTGGTCTTCATTTTATCAACAC

510 520 530 540 550 560 570 580 590 600

....|....|....|....|....|....|....|....|....|....|....|....|....|....|....|....|....|....|....|....|

**HCT_Col-0** 501 ATGGTCTGATATGGCTCGTGGTCTTGACCTAACCATTCCACCTTTCATTGATCGAACACTCCTCCGAGCTAGGGACCCGCCACAGCCTGCTTTTCATCAT

**HCT_Est-1** 501 ATGGTCTGATATGGCTCGTGGTCTTGACCTAACCATTCCACCTTTCATTGATCGAACACTCCTCCGAGCTAGGGACCCGCCACAGCCTGCTTTTCATCAT

**HCT_Tsu-1** 501 ATGGTCTGATATGGCTCGTGGTCTTGACCTAACCATTCCACCTTTCATTGATCGAACACTCCTCCGAGCTAGGGACCCGCCACAGCCTGCTTTTCATCAT

610 620 630 640 650 660 670 680 690 700

....|....|....|....|....|....|....|....|....|....|....|....|....|....|....|....|....|....|....|....|

**HCT_Col-0** 601 GTTGAATATCAGCCTGCACCAAGTATGAAGATACCTCTTGATCCGTCTAAATCAGGACCTGAGAATACCACTGTCTCTATATTCAAATTAACACGAGACC

**HCT_Est-1** 601 GTTGAATATCAGCCTGCACCAAGTATGAAGATACCTCTTGATCCGTCTAAATCAGGACCTGAGAATACCACTGTCTCTATATTCAAATTAACACGAGACC

**HCT_Tsu-1** 601 GTTGAATATCAGCCTGCACCAAGTATGAAGATACCTCTTGATCCGTCTAAATCAGGACCTGAGAATACCACTGTCTCTATATTCAAATTAACACGAGACC

710 720 730 740 750 760 770 780 790 800

....|....|....|....|....|....|....|....|....|....|....|....|....|....|....|....|....|....|....|....|

**HCT_Col-0** 701 AGCTTGTTGCTCTTAAGGCGAAATCCAAGGAGGATGGGAACACTGTCAGCTACAGCTCATACGAGATGTTGGCAGGGCATGTGTGGAGATCAGTGGGAAA

**HCT_Est-1** 701 AGCTTGTTGCTCTTAAGGCGAAATCCAAGGAGGATGGGAACACTGTCAGCTACAGCTCATACGAGATGTTGGCAGGGCATGTGTGGAGATCAGTGGGAAA

**HCT_Tsu-1** 701 AGCTTGTTGCTCTTAAGGCGAAATCCAAGGAGGATGGGAACACTGTCAGCTACAGCTCATACGAGATGTTGGCAGGGCATGTGTGGAGATCAGTGGGAAA

810 820 830 840 850 860 870 880 890 900

....|....|....|....|....|....|....|....|....|....|....|....|....|....|....|....|....|....|....|....|

**HCT_Col-0** 801 GGCGCGAGGGCTTCCAAACGACCAAGAGACGAAACTGTACATTGCAACTGATGGAAGGTCTAGACTACGTCCGCAGCTGCCTCCTGGTTACTTTGGGAAT

**HCT_Est-1** 801 GGCGCGAGGGCTTCCAAACGACCAAGAGACGAAACTGTACATTGCAACTGATGGAAGGTCTAGACTACGTCCGCAGCTGCCTCCTGGTTACTTTGGGAAT

**HCT_Tsu-1** 801 GGCGCGAGGGCTTCCAAACGACCAAGAGACGAAACTGTACATTGCAACTGATGGAAGGTCTAGACTACGTCCGCAGCTGCCTCCTGGTTACTTTGGGAAT

910 920 930 940 950 960 970 980 990 1000

....|....|....|....|....|....|....|....|....|....|....|....|....|....|....|....|....|....|....|....|

**HCT_Col-0** 901 GTGATATTCACTGCAACACCATTGGCTGTTGCAGGGGATTTGTTATCTAAGCCAACATGGTATGCTGCAGGACAGATTCATGATTTCTTGGTTCGTATGG

**HCT_Est-1** 901 GTGATATTCACTGCAACACCATTGGCTGTTGCAGGGGATTTGTTATCTAAGCCAACATGGTATGCTGCAGGACAGATTCATGATTTCTTGGTTCGTATGG

**HCT_Tsu-1** 901 GTGATATTCACTGCAACACCATTGGCTGTTGCAGGGGATTTGTTATCTAAGCCAACATGGTATGCTGCAGGACAGATTCATGATTTCTTGGTTCGTATGG

1010 1020 1030 1040 1050 1060 1070 1080 1090 1100

....|....|....|....|....|....|....|....|....|....|....|....|....|....|....|....|....|....|....|....|

**HCT_Col-0** 1001 ATGATAACTATCTGAGGTCAGCTCTTGACTACCTGGAGATGCAGCCTGATCTGTCAGCCCTTGTCCGCGGTGCACATACCTACAAGTGCCCAAATTTGGG

**HCT_Est-1** 1001 ATGATAACTATCTGAGGTCAGCTCTTGACTACCTGGAGATGCAGCCTGATCTGTCAGCCCTTGTCCGCGGTGCACATACCTACAAGTGCCCAAATTTGGG

**HCT_Tsu-1** 1001 ATGATAACTATCTGAGGTCAGCTCTTGACTACCTGGAGATGCAGCCTGATCTGTCAGCCCTTGTCCGCGGTGCACATACCTACAAGTGCCCAAATTTGGG

1110 1120 1130 1140 1150 1160 1170 1180 1190 1200

....|....|....|....|....|....|....|....|....|....|....|....|....|....|....|....|....|....|....|....|

**HCT_Col-0** 1101 AATCACAAGCTGGGTTAGATTACCTATTTATGATGCAGACTTTGGTTGGGGTCGTCCTATCTTTATGGGACCTGGTGGAATTCCATACGAGGGTTTGTCT

**HCT_Est-1** 1101 AATCACAAGCTGGGTTAGATTACCTATTTATGATGCAGACTTTGGTTGGGGTCGTCCTATATTTATGGGACCTGGTGGAATTCCATACGAGGGTTTGTCT

**HCT_Tsu-1** 1101 AATCACAAGCTGGGTTAGATTACCTATTTATGATGCAGACTTTGGTTGGGGTCGTCCTATATTTATGGGACCTGGTGGAATTCCATACGAGGGTTTGTCT

1210 1220 1230 1240 1250 1260 1270 1280 1290 1300

....|....|....|....|....|....|....|....|....|....|....|....|....|....|....|....|....|....|....|....|

**HCT_Col-0** 1201 TTTGTGCTACCAAGTCCTACTAATGATGGCAGCTTATCCGTTGCCATTGCCCTCCAATCTGAACACATGAAACTGTTTGAGAAGTTTTTGTTTGAGATAT

**HCT_Est-1** 1201 TTTGTGCTACCAAGTCCTACTAATGATGGCAGCTTATCCGTTGCCATTGCCCTCCAATCTGAACACATGAAACTGTTTGAGAAGTTTTTGTTTGAGATAT

**HCT_Tsu-1** 1201 TTTGTGCTACCAAGTCCTACTAATGATGGCAGCTTATCCGTTGCCATTGCCCTCCAATCTGAACACATGAAACTGTTTGAGAAGTTTTTGTTTGAGATAT

..

**HCT_Col-0** 1301 GA

**HCT_Est-1** 1301 GA

**HCT_Tsu-1** 1301 GA

Created with BioEdit version 5.0.9. Muliple sequence alignment: ClustalW

Additional Parameters for ClustalW:

***General settings:****

/QUICKTREE :use FAST algorithm for the alignment guide tree

/NEWTREE= :file for new guide tree

/USETREE= :file for old guide tree

/NEGATIVE :protein alignment with negative values in matrix

***Fast Pairwise Alignments:***

/KTUP=n :word size /TOPDIAGS=n :number of best diags.

/WINDOW=n :window around best diags. /PAIRGAP=n :gap penalty

/SCORE :PERCENT or ABSOLUTE

***Slow Pairwise Alignments:***

/PWMATRIX= :BLOSUM, PAM, ID or filename

/PWGAPOPEN=f :gap opening penalty /PWGAPEXT=f :gap opening penalty

***Multiple Alignments:***

/MATRIX= :BLOSUM, PAM, ID or filename

/GAPOPEN=f :gap opening penalty /GAPEXT=f :gap extension penalty

/ENDGAPS :no end gap separation pen. /GAPDIST=n :gap separation pen. range

/NORGAP :Residue specific gaps off /NOHGAP :hydrophilic gaps off

/HGAPRESIDUES= :list hydrophilic res. /MAXDIV=n :% ident. for delay

/TYPE= :PROTEIN or DNA /TRANSITIONS :transitions NOT weighted.

***Trees:*** /SEED=n :seed number for bootstraps.

/KIMURA :use Kimura's correction. /TOSSGAPS :ignore positions with gaps.
