# Supplementary material for: Genes to specialized metabolites: accumulation of scopoletin, umbelliferone and their glycosides in natural populations of Arabidopsis thaliana
Source: BMC Plant Biol. 2024 Aug 27;24:806. doi: 10.1186/s12870-024-05491-w (PMC11348552; doi:10.1186/s12870-024-05491-w)
Supplement: Supplementary file 7 — Additional file 7_Figure S4. Amino acid sequence variants shown as a Multiple sequence alignment (MSA) [file 12870_2024_5491_MOESM7_ESM.docx]

**Figure S4. Multiple Sequence Alignment (MSA) of tested genes’ protein sequences.** To illustrate conserved and variable regions in selected protein sequences from Col-0, Est-1, and Tsu-1 accessions, amino acid sequences were aligned using the ClustalW algorithm. Grey highlighting indicates similar amino acids across the aligned sequences. Black highlighting marks the positions of different amino acids. MSA was created and visualized using BioEdit v5.0.9 software.

**4-coumarate CoA ligase 1 (4CL1, At1g51680)**

10 20 30 40 50 60 70 80 90 100

....|....|....|....|....|....|....|....|....|....|....|....|....|....|....|....|....|....|....|....|

**4CL1_Col-0** 1 MAPQEQAVSQVMEKQSNNNNSDVIFRSKLPDIYIPNHLSLHDYIFQNISEFATKPCLINGPTGHVYTYSDVHVISRQIAANFHKLGVNQNDVVMLLLPNC

**4CL1_Est-1** 1 MAPQEQAVSQVMEKQSNNNNSDVIFRSKLPDIYIPNHLSLHDYIFQNISEFATKPCLINGPTGHVYTYSDVHVISRQIAANFHKLGVNQNDVVMLLLPNC

**4CL1_Tsu-1** 1 MAPQEQAVSQVMEKQSNNNNSDVIFRSKLPDIYIPNHLSLHDYIFQNISEFATKPCLINGPTGHVYTYSDVHVISRQIAANFHKLGVNQNDVVMLLLPNC

110 120 130 140 150 160 170 180 190 200

....|....|....|....|....|....|....|....|....|....|....|....|....|....|....|....|....|....|....|....|

**4CL1_Col-0** 101 PEFVLSFLAASFRGATATAANPFFTPAEIAKQAKASNTKLIITEARYVDKIKPLQNDDGVVIVCIDDNESVPIPEGCLRFTELTQSTTEASEVIDSVEIS

**4CL1_Est-1** 101 PEFVLSFLAASFRGATATAANPFFTPAEIAKQAKASNTKLIITEARYVDKIKPLQNDDGVVIVCIDDNESVPIPEGCLRFTELTQSTTEASEVIDSVEIS

**4CL1_Tsu-1** 101 PEFVLSFLAASFRGATATAANPFFTPAEIAKQAKASNTKLIITEARYVDKIKPLQNDDGVVIVCIDDNESVPIPEGCLRFTELTQSTTEASEVIDSVEIS

210 220 230 240 250 260 270 280 290 300

....|....|....|....|....|....|....|....|....|....|....|....|....|....|....|....|....|....|....|....|

**4CL1_Col-0** 201 PDDVVALPYSSGTTGLPKGVMLTHKGLVTSVAQQVDGENPNLYFHSDDVILCVLPMFHIYALNSIMLCGLRVGAAILIMPKFEINLLLELIQRCKVTVAP

**4CL1_Est-1** 201 PDDVVALPYSSGTTGLPKGVMLTHKGLVTSVAQQVDGENPNLYFHSDDVILCVLPMFHIYALNSIMLCGLRVGAAILIMPKFEINLLLELIQRCKVTVAP

**4CL1_Tsu-1** 201 PDDVVALPYSSGTTGLPKGVMLTHKGLVTSVAQQVDGENPNLYFHSDDVILCVLPMFHIYALNSIMLCGLRVGAAILIMPKFEINLLLELIQRCKVTVAP

310 320 330 340 350 360 370 380 390 400

....|....|....|....|....|....|....|....|....|....|....|....|....|....|....|....|....|....|....|....|

**4CL1_Col-0** 301 MVPPIVLAIAKSSETEKYDLSSIRVVKSGAAPLGKELEDAVNAKFPNAKLGQGYGMTEAGPVLAMSLGFAKEPFPVKSGACGTVVRNAEMKIVDPDTGDS

**4CL1_Est-1** 301 MVPPIVLAIAKSSETEKYDLSSIRVVKSGAAPLGKELEDAVNAKFPNAKLGQGYGMTEAGPVLAMSLGFAKEPFPVKSGACGTVVRNAEMKIVDPDTGDS

**4CL1_Tsu-1** 301 MVPPIVLAIAKSSETEKYDLSSIRVVKSGAAPLGKELEDAVNAKFPNAKLGQGYGMTEAGPVLAMSLGFAKEPFPVKSGACGTVVRNAEMKIVDPDTGDS

410 420 430 440 450 460 470 480 490 500

....|....|....|....|....|....|....|....|....|....|....|....|....|....|....|....|....|....|....|....|

**4CL1_Col-0** 401 LSRNQPGEICIRGHQIMKGYLNNPAATAETIDKDGWLHTGDIGLIDDDDELFIVDRLKELIKYKGFQVAPAELEALLIGHPDITDVAVVAMKEEAAGEVP

**4CL1_Est-1** 401 LSRNQPGEICIRGHQIMKGYLNNPAATAETIDKDGWLHTGDIGLIDDDDELFIVDRLKELIKYKGFQVAPAELEALLIGHPDITDVAVVAMKEEAAGEVP

**4CL1_Tsu-1** 401 LSRNQPGEICIRGHQIMKGYLNNPAATAETIDKDGWLHTGDIGLIDDDDELFIVDRLKELIKYKGFQVAPAELEALLIGHPDITDVAVVAMKEEAAGEVP

510 520 530 540 550 560

....|....|....|....|....|....|....|....|....|....|....|....|.

**4CL1_Col-0** 501 VAFVVKSKDSELSEDDVKQFVSKQVVFYKRINKVFFTESIPKAPSGKILRKDLRAKLANGL

**4CL1_Est-1** 501 VAFVVKSKDSELSEDDVKQFVSKQVVFYKRINKVFFTESIPKAPSGKILRKDLRAKLANGL

**4CL1_Tsu-1** 501 VAFVVKSKDSELSEDDVKQFVSKQVVFYKRINKVFFTESIPKAPSGKILRKDLRAKLANGL

**4-coumarate CoA ligase 2 (4CL2, At3g21240)**

10 20 30 40 50 60 70 80 90 100

....|....|....|....|....|....|....|....|....|....|....|....|....|....|....|....|....|....|....|....|

**4CL2_Col-0** 1 MTTQDVIVNDQNDQKQCSNDVIFRSRLPDIYIPNHLPLHDYIFENISEFAAKPCLINGPTGEVYTYADVHVTSRKLAAGLHNLGVKQHDVVMILLPNSPE

**4CL2_Est-1** 1 MTTQDVIVNDQNDQKQCSNDVIFRSKLPDIYIPNHLPLHDYIFENISEFAAKPCLINGPTGEVYTYADVHVTSRKLAAGLHNLGVKQHDVVMILLPNSPE

**4CL2_Tsu-1** 1 MTTQDVIVNDQNDQKQCSNDVIFRSRLPDIYIPNHLPLHDYIFENISEFAAKPCLINGPTGEVYTYADVHVTSRKLAAGLHNLGVKQHDVVMILLPNSPE

110 120 130 140 150 160 170 180 190 200

....|....|....|....|....|....|....|....|....|....|....|....|....|....|....|....|....|....|....|....|

**4CL2_Col-0** 101 VVLTFLAASFIGAITTSANPFFTPAEISKQAKASAAKLIVTQSRYVDKIKNLQNDGVLIVTTDSDAIPENCLRFSELTQSEEPRVDSIPEKISPEDVVAL

**4CL2_Est-1** 101 VVLTFLAASFIGAITTSANPFFTPAEISKQAKASAAKLIVTQSRYVDKIKNLQNDGVLIVTTDSDAIPENCLRFSELTQSEEPRVDSIPEKISPEDVVAL

**4CL2_Tsu-1** 101 VVLTFLAASFIGAITTSANPFFTPAEISKQAKASAAKLIVTQSRYVDKIKNLQNDGVLIVTTDSDAIPENCLRFSELTQSEEPRVDSIPEKISPEDVVAL

210 220 230 240 250 260 270 280 290 300

....|....|....|....|....|....|....|....|....|....|....|....|....|....|....|....|....|....|....|....|

**4CL2_Col-0** 201 PFSSGTTGLPKGVMLTHKGLVTSVAQQVDGENPNLYFNRDDVILCVLPMFHIYALNSIMLCSLRVGATILIMPKFEITLLLEQIQRCKVTVAMVVPPIVL

**4CL2_Est-1** 201 PFSSGTTGLPKGVMLTHKGLVTSVAQQVDGENPNLYFNRDDVILCVLPMFHIYALNSIMLCSLRVGATILIMPKFEITLLLEQIQRCKVTVAMVVPPIVL

**4CL2_Tsu-1** 201 PFSSGTTGLPKGVMLTHKGLVTSVAQQVDGENPNLYFNRDDVILCVLPMFHIYALNSIMLCSLRVGATILIMPKFEITLLLEQIQRCKVTVAMVVPPIVL

310 320 330 340 350 360 370 380 390 400

....|....|....|....|....|....|....|....|....|....|....|....|....|....|....|....|....|....|....|....|

**4CL2_Col-0** 301 AIAKSPETEKYDLSSVRMVKSGAAPLGKELEDAISAKFPNAKLGQGYGMTEAGPVLAMSLGFAKEPFPVKSGACGTVVRNAEMKILDPDTGDSLPRNKPG

**4CL2_Est-1** 301 AIAKSPETEKYDLSSVRMVKSGAAPLGKELEDAISAKFPNAKLGQGYGMTEAGPVLAMSLGFAKEPFPVKSGACGTVVRNAEMKILDPDTGDSLPRNKPG

**4CL2_Tsu-1** 301 AIAKSPETEKYDLSSVRMVKSGAAPLGKELEDAISAKFPNAKLGQGYGMTEAGPVLAMSLGFAKEPFPVKSGACGTVVRNAEMKILDPDTGDSLPRNKPG

410 420 430 440 450 460 470 480 490 500

....|....|....|....|....|....|....|....|....|....|....|....|....|....|....|....|....|....|....|....|

**4CL2_Col-0** 401 EICIRGNQIMKGYLNDPLATASTIDKDGWLHTGDVGFIDDDDELFIVDRLKELIKYKGFQVAPAELESLLIGHPEINDVAVVAMKEEDAGEVPVAFVVRS

**4CL2_Est-1** 401 EICIRGNQIMKGYLNDPLATASTIDKDGWLHTGDVGFIDDDDELFIVDRLKELIKYKGFQVAPAELESLLIGHPEINDVAVVAMKEEDAGEVPVAFVVRS

**4CL2_Tsu-1** 401 EICIRGNQIMKGYLNDPLATASTIDKDGWLHTGDVGFIDDDDELFIVDRLKELIKYKGFQVAPAELESLLIGHPEINDVAVVAMKEEDAGEVPVAFVVRS

510 520 530 540 550

....|....|....|....|....|....|....|....|....|....|....|.

**4CL2_Col-0** 501 KDSNISEDEIKQFVSKQVVFYKRINKVFFTDSIPKAPSGKILRKDLRARLANGLMN

**4CL2_Est-1** 501 KDSNISEDEIKQFVSKQVVFYKRINKVFFTDSIPKAPSGKILRKDLRARLANGLMN

**4CL2_Tsu-1** 501 KDSNISEDEIKQFVSKQVVFYKRINKVFFTDSIPKAPSGKILRKDLRARLANGLMN

**4-coumarate CoA ligase 3 (4CL3, At1g65060)**

10 20 30 40 50 60 70 80 90 100

....|....|....|....|....|....|....|....|....|....|....|....|....|....|....|....|....|....|....|....|

**4CL3_Col-0** 1 MITAALHEPQIHKPTDTSVVSDDVLP---HSPPTPRIFRSKLPDIDIPNHLPLHTYCFEKLSSVSDKPCLIVGSTGKSYTYGETHLICRRVASGLYKLGI

**4CL3_Est-1** 1 MITAALHEPQIHKPSDTSVVSGDVLPPPPPSPPTPRIFRSKLPDINIPNHLPLHTYCFEKLSSVSDKPCLIVGSTGKSYTYGETHLICRRVASGLCKLGI

**4CL3_Tsu-1** 1 MITAALHEPQIHKPTDTSVVSDDVLP---HSPPTPRIFRSKLPDIDIPNHLPLHTYCFEKLSSVSDKPCLIVGSTGKSYTYGETHLICRRVASGLYKLGI

110 120 130 140 150 160 170 180 190 200

....|....|....|....|....|....|....|....|....|....|....|....|....|....|....|....|....|....|....|....|

**4CL3_Col-0** 98 RKGDVIMILLQNSAEFVFSFMGASMIGAVSTTANPFYTSQELYKQLKSSGAKLIITHSQYVDKLKNLGENLTLITTDEPTPENCLPFSTLITDDETNPFQ

**4CL3_Est-1** 101 RKGDVIMILLQNSAEFVFSFMGASMIGAVSTTANPFYTSQELYKQLKSSGAKLIITHSQYVDKLKNLGENLTVITTDEPTPENCLPFSTLITDDETNPFQ

**4CL3_Tsu-1** 98 RKGDVIMILLQNSAEFVFSFMGASMIGAVSTTANPFYTSQELYKQLKSSGAKLIITHSQYVDKLKNLGENLTLITTDEPTPENCLPFSTLITDDETNPFQ

210 220 230 240 250 260 270 280 290 300

....|....|....|....|....|....|....|....|....|....|....|....|....|....|....|....|....|....|....|....|

**4CL3_Col-0** 198 ETVDIGGDDAAALPFSSGTTGLPKGVVLTHKSLITSVAQQVDGDNPNLYLKSNDVILCVLPLFHIYSLNSVLLNSLRSGATVLLMHKFEIGALLDLIQRH

**4CL3_Est-1** 201 ETVDIGGDDAAALPFSSGTTGLPKGVVLTHKSLITSVAQQVDGDNPNLYLKSNDVILCVLPLFHIYSLNSVLLNSLRSGATVLLMHKFEIGALLDLIQRH

**4CL3_Tsu-1** 198 ETVDIGGDDAAALPFSSGTTGLPKGVVLTHKSLITSVAQQVDGDNPNLYLKSNDVILCVLPLFHIYSLNSVLLNSLRSGATVLLMHKFEIGALLDLIQRH

310 320 330 340 350 360 370 380 390 400

....|....|....|....|....|....|....|....|....|....|....|....|....|....|....|....|....|....|....|....|

**4CL3_Col-0** 298 RVTIAALVPPLVIALAKNPTVNSYDLSSVRFVLSGAAPLGKELQDSLRRRLPQAILGQGYGMTEAGPVLSMSLGFAKEPIPTKSGSCGTVVRNAELKVVH

**4CL3_Est-1** 301 RVTIAALVPPLVIALAKNPTVNSYDLSSVRFVLSGAAPLGKELQDSLRRRLPQAILGQGYGMTEAGPVLSMSLGFAKEPIPTKSGSCGTVVRNAELKVVH

**4CL3_Tsu-1** 298 RVTIAALVPPLVIALAKNPTVNSYDLSSVRFVLSGAAPLGKELQDSLRRRLPQAILGQGYGMTEAGPVLSMSLGFAKEPIPTKSGSCGTVVRNAELKVVH

410 420 430 440 450 460 470 480 490 500

....|....|....|....|....|....|....|....|....|....|....|....|....|....|....|....|....|....|....|....|

**4CL3_Col-0** 398 LETRLSLGYNQPGEICIRGQQIMKEYLNDPEATSATIDEEGWLHTGDIGYVDEDDEIFIVDRLKEVIKFKGFQVPPAELESLLINHHSIADAAVVPQNDE

**4CL3_Est-1** 401 LETRLSLGYNQPGEICIRGQQIMKEYLNDPEATSATIDEEGWLHTGDIGYVDEDDEIFIVDRLKEVIKFKGFQVPPAELESLLINHHSIADAAVVPQNDE

**4CL3_Tsu-1** 398 LETRLSLGYNQPGEICIRGQQIMKEYLNDPEATSATIDEEGWLHTGDIGYVDEDDEIFIVDRLKEVIKFKGFQVPPAELESLLINHHSIADAAVVPQNDE

510 520 530 540 550 560

....|....|....|....|....|....|....|....|....|....|....|....|....

**4CL3_Col-0** 498 VAGEVPVAFVVRSNGNDITEEDVKEYVAKQVVFYKRLHKVFFVASIPKSPSGKILRKDLKAKLC

**4CL3_Est-1** 501 VAGEVPVAFVVRSNGNDITEEDVKEYVAKQVVFYKRLHKVFFVASIPKSPSGKILRKDLKAKLC

**4CL3_Tsu-1** 498 VAGEVPVAFVVRSNGNDITEEDVKEYVAKQVVFYKRLHKVFFVASIPKSPSGKILRKDLKAKLC

**Feruloyl-CoA 6-Hydroxylase 1 (F6H1, At3g13610)**

10 20 30 40 50 60 70 80 90 100

....|....|....|....|....|....|....|....|....|....|....|....|....|....|....|....|....|....|....|....|

**F6H1_Col-0** 1 MAPTLLTTQFSNPAEVTDFVVYKGNGVKGLSETGIKALPEQYIQPLEERLINKFVNETDEAIPVIDMSNPDEDRVAEAVCDAAEKWGFFQVINHGVPLEV

**F6H1_Est-1** 1 MAPTLLTTQFSNPAEVTDFVVYKGNGVKGLSETGIKALPEQYIQPLEERLINKFVNETDEAIPVIDMSNPDEDRVAEAVCDAAEKWGFFQVINHGVPLEV

**F6H1_Tsu-1** 1 MAPTLLTTQFSNPAEVTDFVVYKGNGVKGLSETGIKALPEQYIQPLEERLINKFVNETDEAIPVIDMSNLDEDRVAEAVCDAAEKWGFFQVINHGVPLEV

110 120 130 140 150 160 170 180 190 200

....|....|....|....|....|....|....|....|....|....|....|....|....|....|....|....|....|....|....|....|

**F6H1_Col-0** 101 LDDVKAATHKFFNLPVEEKRKFTKENSLSTTVRFGTSFSPLAEQALEWKDYLSLFFVSEAEAEQFWPDICRNETLEYINKSKKMVRRLLEYLGKNLNVKE

**F6H1_Est-1** 101 LDDVKAATHKFFNLPVEEKRKFTKENSLSTTVRFGTSFSPLAEQALEWKDYLSLFFVSEAEAEQFWPDICRNETLEYINKSKKMVRRLLEYLGKNLNVKE

**F6H1_Tsu-1** 101 LDDVKAATHKFFNLPVEEKRKFTKENSLSTTVRFGTSFSPLAEQALEWKDYLSLFFVSEAEAEQFWPDICRNETLEYINKSKKMVRRLLEYLGKNLNVKE

210 220 230 240 250 260 270 280 290 300

....|....|....|....|....|....|....|....|....|....|....|....|....|....|....|....|....|....|....|....|

**F6H1_Col-0** 201 LDETKESLFMGSIRVNLNYYPICPNPDLTVGVGRHSDVSSLTILLQDQIGGLHVRSLASGNWVHVPPVAGSFVINIGDAMQIMSNGLYKSVEHRVLANGY

**F6H1_Est-1** 201 LDETKESLFMGSIRVNLNYYPICPNPDLTVGVGRHSDVSSLTILLQDQIGGLHVRSLASGNWVHVPPVAGSFVINIGDAMQIMSNGLYKSVEHRVLANGY

**F6H1_Tsu-1** 201 LDETKESLFMGSIRVNLNYYPICPNPDLTVGVGRHSDVSSLTILLQDQIGGLHVRSLASGNWVHVPPVAGSFVINIGDAMQIMSNGLYKSVEHRVLANGY

310 320 330 340 350 360

....|....|....|....|....|....|....|....|....|....|....|....|.

**F6H1_Col-0** 301 NNRISVPIFVNPKPESVIGPLPEVIANGEEPIYRDVLYSDYVKYFFRKAHDGKKTVDYAKI

**F6H1_Est-1** 301 NNRISVPIFVNPKPESVIGPLPEVIANGEEPIYRDVLYSDYVKYFFRKAHDGKKTVDYAKI

**F6H1_Tsu-1** 301 NNRISVPIFVNPKPESVIGPLPEVIANGEEPIYRDVLYSDYVKYFFRKAHDGKKTVDYAKI

**Feruloyl-CoA 6-Hydroxylase 2(F6H2, At1g55290)**

10 20 30 40 50 60 70 80 90 100

....|....|....|....|....|....|....|....|....|....|....|....|....|....|....|....|....|....|....|....|

**F6’H2_Col-0** 1 MNQTLAAQFLTRDQVTNFVVHEGNGVKGLSETGIKVLPDQYIQPFEERLINFHVKEDSDEYIQPFEERLINFHVKEDSDESIPVIDISNLDEKSVSKAVC

**F6’H2_Est-1** 1 MNQTLAAQFLTPDQVTNFVVHEGNGVKGLSETGIKVLPDQYIQPFEERLINFHVKEDSDEYIQPFEERLINFHVKEDSDESIPVIDISNLDEKSVSKAVC

**F6’H2_Tsu-1** 1 MNQTLAAQFLTRDQVTNFVVHEGNGVKGLSETGIKVLPDQYIQPFEERLINFHVKEDSDEYIQPFEERLINFHVKEDSDESIPVIDMSNLDEKSVSKAVC

110 120 130 140 150 160 170 180 190 200

....|....|....|....|....|....|....|....|....|....|....|....|....|....|....|....|....|....|....|....|

**F6’H2_Col-0** 101 DAAEEWGFFQVINHGVSMEVLENMKTATHRFFGLPVEEKRKFSREKSLSTNVRFGTSFSPHAEKALEWKDYLSLFFVSEAEASQLWPDSCRSETLEYMNE

**F6’H2_Est-1** 101 DAAEEWGFFQVINHGVSMEVLENMKTATHRFFGLPVEEKRKFSREKSLSTNVRFGTSFSPHAEKALEWKDYLSLFFVSEAEASQLWPDSCRSETLEYMNE

**F6’H2_Tsu-1** 101 DAAEEWGFFQVINHGVSMEVLENMKTATHRFFGLPVEEKRKFSREKSLSTNVRFGTSFSPHAEKALEWKDYLSLFFVSEAEASQLWPDSCRSETLEYMNE

210 220 230 240 250 260 270 280 290 300

....|....|....|....|....|....|....|....|....|....|....|....|....|....|....|....|....|....|....|....|

**F6’H2_Col-0** 201 TKPLVKKLLRFLGENLNVKELDKTKESFFMGSTRINLNYYPICPNPELTVGVGRHSDVSSLTILLQDEIGGLHVRSLTTGRWVHVPPISGSLVINIGDAM

**F6’H2_Est-1** 201 TKPLVKKLLRFLGENLNVKELDKTKESFFMGSTRINLNYYPICPNPELTVGVGRHSDVSSLTILLQDEIGGLHVRSLTTGRWVHVPPISGSLVINIGDAM

**F6’H2_Tsu-1** 201 TKPLVKKLLRFLGENLNVTELDKTKESLFMGSTRINLNYYPICPNPELTVGVGRHSDVSSLTILLQDEIGGLHVRSLTTGRWVHVPPISGSLVINIGDAM

310 320 330 340 350 360 370 380

....|....|....|....|....|....|....|....|....|....|....|....|....|....|....|....|.

**F6’H2_Col-0** 301 QIMSNGRYKSVEHRVLANGSYNRISVPIFVSPKPESVIGPLLEVIENGEKPVYKDILYTDYVKHFFRKAHDGKKTIDFANI

**F6’H2_Est-1** 301 QIMSNGRYKSVEHRVLANGSYNRISVPIFVSPKPESVIGPLLEVIENGEKPVYKDILYTDYVKHFFRKAHDGKKTIDFANI

**F6’H2_Tsu-1** 301 QIMSNGRYKSVEHRVLANSSYNRISVPIFVNPKPESVIGPLLEVIENGEKPVYRDILYTDYVKHFFRKAHDGKKTIDFANI

**p-coumaroyl 3-hydroxylase (C3H, At2g40890)**

10 20 30 40 50 60 70 80 90 100

....|....|....|....|....|....|....|....|....|....|....|....|....|....|....|....|....|....|....|....|

**C3H_Col-0** 1 MSWFLIAVATIAAVVSYKLIQRLRYKFPPGPSPKPIVGNLYDIKPVRFRCYYEWAQSYGPIISVWIGSILNVVVSSAELAKEVLKEHDQKLADRHRNRST

**C3H_Est-1** 1 MSWFLIAVATIAAVVSYKLIQRLRYKFPPGPSPKPIVGNLYDIKPVRFRCYYEWAQSYGPIISVWIGSILNVVVSSAELAKEVLKEHDQKLADRHRNRST

**C3H_Tsu-1** 1 MSWFLIAVATIAAVVSYKLIQRLRYKFPPGPSPKPIVGNLYDIKPVRFRCYYEWAQSYGPIISVWIGSILNVVVSSAELAKEVLKEHDQKLADRHRNRST

110 120 130 140 150 160 170 180 190 200

....|....|....|....|....|....|....|....|....|....|....|....|....|....|....|....|....|....|....|....|

**C3H_Col-0** 101 EAFSRNGQDLIWADYGPHYVKVRKVCTLELFTPKRLESLRPIREDEVTAMVESVFRDCNLPENRAKGLQLRKYLGAVAFNNITRLAFGKRFMNAEGVVDE

**C3H_Est-1** 101 EAFSRNGQDLIWADYGPHYVKVRKVCTLELFTPKRLESLRPIREDEVTAMVESVFRDCNLPENRAKGLQLRKYLGAVAFNNITRLAFGKRFMNAEGVVDE

**C3H_Tsu-1** 101 EAFSRNGQDLIWADYGPHYVKVRKVCTLELFTPKRLESLRPIREDEVTAMVESVFRDCNLPENRAKGLQLRKYLGAVAFNNITRLAFGKRFMNAEGVVDE

210 220 230 240 250 260 270 280 290 300

....|....|....|....|....|....|....|....|....|....|....|....|....|....|....|....|....|....|....|....|

**C3H_Col-0** 201 QGLEFKAIVSNGLKLGASLSIAEHIPWLRWMFPADEKAFAEHGARRDRLTRAIMEEHTLARQKSSGAKQHFVDALLTLKDQYDLSEDTIIGLLWDMITAG

**C3H_Est-1** 201 QGLEFKAIVSNGLKLGASLSIAEHIPWLRWMFPADEKAFAEHGARRDRLTRAIMEEHTLARQKSSGAKQHFVDALLTLKDQYDLSEDTIIGLLWDMITAG

**C3H_Tsu-1** 201 QGLEFKAIVSNGLKLGASLSIAEHIPWLRWMFPADEKAFAEHGARRDRLTRAIMEEHTLARQKSSGAKQHFVDALLTLKDQYDLSEDTIIGLLWDMITAG

310 320 330 340 350 360 370 380 390 400

....|....|....|....|....|....|....|....|....|....|....|....|....|....|....|....|....|....|....|....|

**C3H_Col-0** 301 MDTTAITAEWAMAEMIKNPRVQQKVQEEFDRVVGLDRILTEADFSRLPYLQCVVKESFRLHPPTPLMLPHRSNADVKIGGYDIPKGSNVHVNVWAVARDP

**C3H_Est-1** 301 MDTTAITAEWAMAEMIKNPRVQQKVQEEFDRVVGLDRILTEADFSRLPYLQCVVKESFRLHPPTPLMLPHRSNADVKIGGYDIPKGSNVHVNVWAVARDP

**C3H_Tsu-1** 301 MDTTAITAEWAMAEMIKNPRVQQKVQEEFDRVVGLDRILTEADFSRLPYLQCVVKESFRLHPPTPLMLPHRSNADVKIGGYDIPKGSNVHVNVWAVARDP

410 420 430 440 450 460 470 480 490 500

....|....|....|....|....|....|....|....|....|....|....|....|....|....|....|....|....|....|....|....|

**C3H_Col-0** 401 AVWKNPFEFRPERFLEEDVDMKGHDFRLLPFGAGRRVCPGAQLGINLVTSMMSHLLHHFVWTPPQGTKPEEIDMSENPGLVTYMRTPVQAVATPRLPSDL

**C3H_Est-1** 401 AVWKNPFEFRPERFLEEDVDMKGHDFRLLPFGAGRRVCPGAQLGINLVTSMMSHLLHHFVWTPPQGTKPEEIDMSENPGLVTYMRTPVQAVATPRLPSDL

**C3H_Tsu-1** 401 AVWKNPFEFRPERFLEEDVDMKGHDFRLLPFGAGRRVCPGAQLGINLVTSMMSHLLHHFVWTPPQGTKPEEIDMSENPGLVTYMRTPVQAVATPRLPSDL

....|...

**C3H_Col-0** 501 YKRVPYDM

**C3H_Est-1** 501 YKRVPYDM

**C3H_Tsu-1** 501 YKRVPYDM

**shikimate O-hydroxycinnamoyltransferase (HCT, At5g48930)**

10 20 30 40 50 60 70 80 90 100

....|....|....|....|....|....|....|....|....|....|....|....|....|....|....|....|....|....|....|....|

**HCT_Col-0** 1 MKINIRDSTMVRPATETPITNLWNSNVDLVIPRFHTPSVYFYRPTGASNFFDPQVMKEALSKALVPFYPMAGRLKRDDDGRIEIDCNGAGVLFVVADTPS

**HCT_Est-1** 1 MKINIRDSTMVRPATETPITNLWNSNVDLVIPRFHTPSVYFYRPTGASNFFDPQVMKEALSKALVPFYPMAGRLKRDDDGRIEIDCNGAGVLFVVADTPS

**HCT_Tsu-1** 1 MKINIRDSTMVRPATETPITNLWNSNVDLVIPRFHTPSVYFYRPTGASNFFDPQVMKEALSKALVPFYPMAGRLKRDDDGRIEIDCNGAGVLFVVADTPS

110 120 130 140 150 160 170 180 190 200

....|....|....|....|....|....|....|....|....|....|....|....|....|....|....|....|....|....|....|....|

**HCT_Col-0** 101 VIDDFGDFAPTLNLRQLIPEVDHSAGIHSFPLLVLQVTFFKCGGASLGVGMQHHAADGFSGLHFINTWSDMARGLDLTIPPFIDRTLLRARDPPQPAFHH

**HCT_Est-1** 101 VIDDFGDFAPTLNLRQLIPEVDHSTGIHSFPLLVLQVTFFKCGGASLGVGMQHHAADGFSGLHFINTWSDMARGLDLTIPPFIDRTLLRARDPPQPAFHH

**HCT_Tsu-1** 101 VIDDFGDFAPTLNLRQLIPEVDHSTGIHSFPLLVLQVTFFKCGGASLGVGMQHHAADGFSGLHFINTWSDMARGLDLTIPPFIDRTLLRARDPPQPAFHH

210 220 230 240 250 260 270 280 290 300

....|....|....|....|....|....|....|....|....|....|....|....|....|....|....|....|....|....|....|....|

**HCT_Col-0** 201 VEYQPAPSMKIPLDPSKSGPENTTVSIFKLTRDQLVALKAKSKEDGNTVSYSSYEMLAGHVWRSVGKARGLPNDQETKLYIATDGRSRLRPQLPPGYFGN

**HCT_Est-1** 201 VEYQPAPSMKIPLDPSKSGPENTTVSIFKLTRDQLVALKAKSKEDGNTVSYSSYEMLAGHVWRSVGKARGLPNDQETKLYIATDGRSRLRPQLPPGYFGN

**HCT_Tsu-1** 201 VEYQPAPSMKIPLDPSKSGPENTTVSIFKLTRDQLVALKAKSKEDGNTVSYSSYEMLAGHVWRSVGKARGLPNDQETKLYIATDGRSRLRPQLPPGYFGN

310 320 330 340 350 360 370 380 390 400

....|....|....|....|....|....|....|....|....|....|....|....|....|....|....|....|....|....|....|....|

**HCT_Col-0** 301 VIFTATPLAVAGDLLSKPTWYAAGQIHDFLVRMDDNYLRSALDYLEMQPDLSALVRGAHTYKCPNLGITSWVRLPIYDADFGWGRPIFMGPGGIPYEGLS

**HCT_Est-1** 301 VIFTATPLAVAGDLLSKPTWYAAGQIHDFLVRMDDNYLRSALDYLEMQPDLSALVRGAHTYKCPNLGITSWVRLPIYDADFGWGRPIFMGPGGIPYEGLS

**HCT_Tsu-1** 301 VIFTATPLAVAGDLLSKPTWYAAGQIHDFLVRMDDNYLRSALDYLEMQPDLSALVRGAHTYKCPNLGITSWVRLPIYDADFGWGRPIFMGPGGIPYEGLS

410 420 430

....|....|....|....|....|....|...

**HCT_Col-0** 401 FVLPSPTNDGSLSVAIALQSEHMKLFEKFLFEI

**HCT_Est-1** 401 FVLPSPTNDGSLSVAIALQSEHMKLFEKFLFEI

**HCT_Tsu-1** 401 FVLPSPTNDGSLSVAIALQSEHMKLFEKFLFEI

Created with BioEdit version 5.0.9. Muliple sequence alignment: ClustalW

Additional Parameters for ClustalW:

***General settings:****

/QUICKTREE :use FAST algorithm for the alignment guide tree

/NEWTREE= :file for new guide tree

/USETREE= :file for old guide tree

/NEGATIVE :protein alignment with negative values in matrix

***Fast Pairwise Alignments:***

/KTUP=n :word size /TOPDIAGS=n :number of best diags.

/WINDOW=n :window around best diags. /PAIRGAP=n :gap penalty

/SCORE :PERCENT or ABSOLUTE

***Slow Pairwise Alignments:***

/PWMATRIX= :BLOSUM, PAM, ID or filename

/PWGAPOPEN=f :gap opening penalty /PWGAPEXT=f :gap opening penalty

***Multiple Alignments:***

/MATRIX= :BLOSUM, PAM, ID or filename

/GAPOPEN=f :gap opening penalty /GAPEXT=f :gap extension penalty

/ENDGAPS :no end gap separation pen. /GAPDIST=n :gap separation pen. range

/NORGAP :Residue specific gaps off /NOHGAP :hydrophilic gaps off

/HGAPRESIDUES= :list hydrophilic res. /MAXDIV=n :% ident. for delay

/TYPE= :PROTEIN or DNA /TRANSITIONS :transitions NOT weighted.

***Trees:*** /SEED=n :seed number for bootstraps.

/KIMURA :use Kimura's correction. /TOSSGAPS :ignore positions with gaps.
